# Genome Wide Analysis of Acute Myeloid Leukemia Reveal Leukemia Specific Methylome and Subtype Specific Hypomethylation of Repeats

**DOI:** 10.1371/journal.pone.0033213

**Published:** 2012-03-29

**Authors:** Marwa H. Saied, Jacek Marzec, Sabah Khalid, Paul Smith, Thomas A. Down, Vardhman K. Rakyan, Gael Molloy, Manoj Raghavan, Silvana Debernardi, Bryan D. Young

**Affiliations:** 1 Centre of Haemato-Oncology, Barts Cancer Institute, Barts and the London School of Medicine, Queen Mary University, London, United Kingdom; 2 Department of Genetics, Wellcome Trust Cancer Research UK Gurdon Institute, University of Cambridge, Cambridge, United Kingdom; 3 Institute of Cell and Molecular Science, School of Medicine, Barts, Queen Mary University of London, London, United Kingdom; 4 Faculty of Medicine, Department of Clinical Pathology, Alexandria University, Alexandria, Egypt; UCLA-DOE Institute for Genomics and Proteomics, United States of America

## Abstract

Methylated DNA immunoprecipitation followed by high-throughput sequencing (MeDIP-seq) has the potential to identify changes in DNA methylation important in cancer development. In order to understand the role of epigenetic modulation in the development of acute myeloid leukemia (AML) we have applied MeDIP-seq to the DNA of 12 AML patients and 4 normal bone marrows. This analysis revealed leukemia-associated differentially methylated regions that included gene promoters, gene bodies, CpG islands and CpG island shores. Two genes (*SPHKAP* and *DPP6*) with significantly methylated promoters were of interest and further analysis of their expression showed them to be repressed in AML. We also demonstrated considerable cytogenetic subtype specificity in the methylomes affecting different genomic features. Significantly distinct patterns of hypomethylation of certain interspersed repeat elements were associated with cytogenetic subtypes. The methylation patterns of members of the SINE family tightly clustered all leukemic patients with an enrichment of Alu repeats with a high CpG density (P<0.0001). We were able to demonstrate significant inverse correlation between intragenic interspersed repeat sequence methylation and gene expression with SINEs showing the strongest inverse correlation (R^2^ = 0.7). We conclude that the alterations in DNA methylation that accompany the development of AML affect not only the promoters, but also the non-promoter genomic features, with significant demethylation of certain interspersed repeat DNA elements being associated with AML cytogenetic subtypes. MeDIP-seq data were validated using bisulfite pyrosequencing and the Infinium array.

## Introduction

Acute myeloid leukemia (AML) is a heterogeneous form of cancer in which many molecular and cytogenetic somatically acquired events have been described [1]. Although these somatic changes have considerable influence over clinical outcome and constitute valuable biomarkers for disease classification [2] their role in the evolution of the stem cell to a fully transformed leukemic cell has yet to be completely understood. It is clear that some of the genes altered in AML play key roles in epigenetic regulation both at the DNA and chromatin levels. The *MLL* gene is involved in many chromosomal translocations in leukemia [3] and can also be altered by partial tandem duplication. This gene is now recognized as a histone methyltransferase. The *EZH2* gene, which encodes a histone methyltransferase, is subject to inactivating monoallelic and biallelic mutations in myelodysplastic/myeloproliferative neoplasms [4]. The potential significance of DNA methylation changes in AML has been given further emphasis with the recent discovery of somatic mutations in *DNMT3A*, which encodes a DNA methyltransferase [5]. There is therefore a considerable body of evidence that implicates epigenetic alterations as being important in the development of AML [6]. However, in order to fully understand the role of DNA methylation in AML, a global view of the AML methylome is required.

MeDIP (methylated DNA immunoprecipitation) is one of the main approaches for detection of DNA methylation [7]. The introduction of next generation sequencing extends the study of DNA to yield whole genome methylation analysis [8]. MeDIP-seq (MeDIP followed by high-throughput sequencing) can investigate the entire genome in an unbiased manner in contrast to array-based methods, which analyze pre-identified sequences [9], [10]. We therefore used this approach to develop whole-genome DNA methylation profiles with a view to the identification of epigenetic features relevant to the development of AML and some of its subtypes. We mapped complete methylomes from 12 AML samples including 4 different cytogenetic subtypes [1] and from 4 normal bone marrows (NBMs) using antibody-mediated enrichment of methylated DNA. Using a previously published method (Batman algorithm) [11], [12], we were able to construct 100 bp resolution methylation map for each leukemia and NBM. That enabled us to identify significant methylation differences in the promoters, the non-promoter genomic regions and also in repeat elements.

## Results

In order to perform a genome wide analysis of DNA methylation in AML, we applied the MeDIP-seq technique to DNA samples from 12 AML patients: 3 with t(8;21) translocation, 3 with the t(15;17) translocation, 3 with trisomy 8, and 3 with a normal karyotype (NK). We used 4 unrelated NBMs as control samples (Table S1). A total of 7.6×10^8^ reads were generated of which 53% could be mapped uniquely to the reference human genome (NCBI 36/hg18). The coverage of the 27 million CpG sites in the reference genome ranged from 63% to 87% for the 16 datasets (Table S2). Furthermore, the saturation analysis indicated that sufficient numbers of reads had been obtained to generate reliable methylome profiles for each DNA sample (Fig. S1) [13]. For interpretation of DNA methylation signals, we used the Batman algorithm [11], [12], which takes into account the underlying CpG density to obtain quantification of methylation (scores being given in the range 0–1). MeDIP-seq results were validated by both the Illumina Infinium array platform and bisulfite pyrosequencing of individual regions (see below). Three DNA samples (studies No. 11, 12 and 13) were analyzed on the Illumina HumanMethylation 27 BeadArray. The methylation levels at 27,578 CpG sites of the array were compared to equivalent values from MeDIP-seq results. Strong correlations between both methods were found (R^2^ = 0.89, 0.9 and 0.8 respectively) (Fig. S2). These correlations between MeDIP-seq and Infinium array are consistent with and slightly higher than previously reported correlations between both methods (R^2^ = 0.8) [12].

### Global DNA methylation assessment in AML and NBM

**Figure 1:**
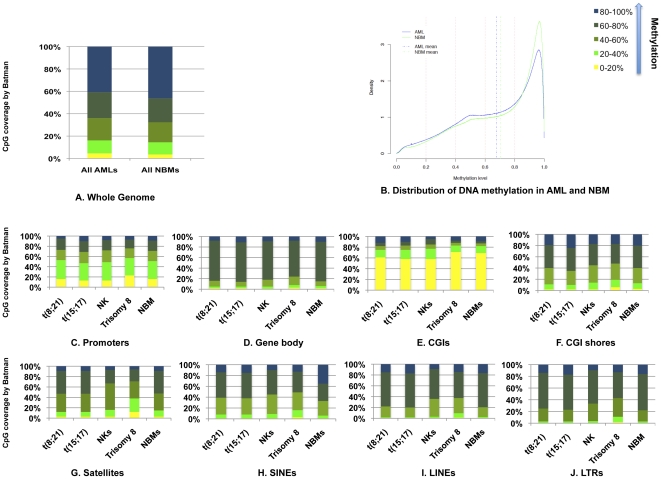
Global DNA methylation display in AML and NBM. (A) DNA methylation of all AML patients and all NBMs were categorized into 5 groups of methylation. There was no significant difference in the global DNA methylation between AML and NBM. (B) DNA methylation scores of all AMLs (blue line) and all NBMs (green line) were plotted against their density (frequency). AML has less frequency of DNA methylation scores>0.8 in the comparison with NBM. (C–J) Percentages of different groups of DNA methylation in the average of each triplicate of AML subtype and in the average of 4 NBMs. SINEs showed the highest difference in the DNA methylation scores>0.8 between NBM and AML.

Firstly, we analyzed AML and NBM whole genome data to assess the significance of differences in global DNA methylation. We categorized DNA methylation into 5 groups; <0.2, 0.2–0.4, 0.4–0.6, 0.6–0.8, 0.8–1.0 Batman scores [11] (Fig. 1A). Fisher's exact test did not demonstrate significant difference in global DNA methylation between all AMLs and all NBMs (P = 0.96). There was only 2.68% difference in the global DNA methylation; AML DNA methylation average was 67.68% while for NBM DNA methylation average was 70.36%. The frequency of Batman scores>0.8 was less in AML than in NBM as some regions of the genome i.e. gene bodies and repeated DNA sequences lose their DNA methylation in cancer (global hypomethylation) [14] (Fig. 1B). To investigate this further, the genome was subdivided into 4 features; promoters, gene bodies, CpG islands (CGIs), and CGI shores (description for each genomic region is summarized in Table S3) and 4 repeat classes (satellites, SINEs; short interspersed nuclear elements, LINEs; long interspersed nuclear elements, and LTRs; long terminal repeats) (Figs. 1C–J). Among these features, gene bodies (Fig. 1D) exhibited the highest level of DNA methylation (82% in AML and 85% in NBM having a Batman score>0.6). The difference in the percentages between AML and NBM is consistent with the global hypomethylation that is a feature of cancer [15]. By contrast, CGIs (Fig. 1E) showed the lowest levels of DNA methylation (16% in AML and 13% in NBM having a Batman score>0.6). CGIs are generally protected from being methylated in normal tissues, whereas in malignancy some CGIs are targets for DNA methylation [6]. Global hypomethylation in AML was mainly observed in SINEs since we noted that ∼20% fewer SINE repeats in AML with a Batman score>0.8 (Fig. 1H). SINEs, especially the Alu family, are rich in methylated CpGs and are common targets for DNA methylation in normal tissues [16].

### Methylation of localized genomic regions discriminates AML and its subtypes

**Figure 2:**
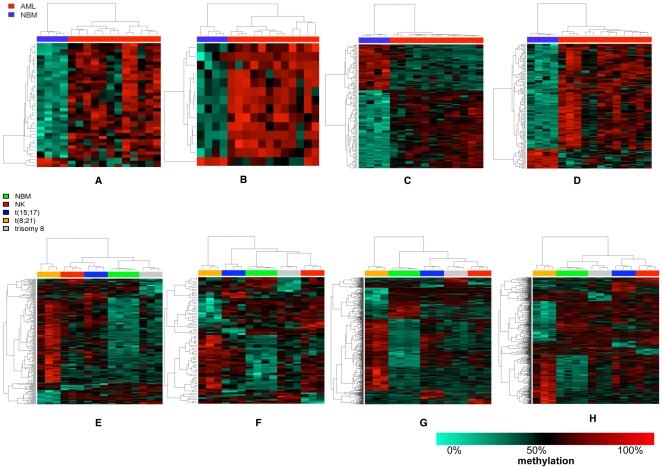
Hierarchical clustering of AML versus NBM in 4 genomic features. First row represents cluster analysis of all AMLs versus all NBMs and the second row represents cluster analysis of AML subtypes in promoters (A, E), gene bodies (B, F), CGIs (C, G) and CGI shores (D, H). In each figure, each column represents AML patient/NBM and each row represents a single DMR. AML patients were clustered more tightly in CGIs (first row). t(8;21) AML subtype was clustered separately from the other AML subtypes (second row).

Next, we investigated whether methylation of specific genomic features could discriminate between AML and NBM. Consequently, we searched for differentially methylated regions (DMRs) in 4 genomic categories; promoters, gene bodies, CGIs and CGI shores using empirical Bayes statistics (Bioconductor's Limma R package). This analysis indicated that 105 gene promoters and 704 CGIs showed significant differences in methylation between AML and NBM (Table S4). 80% of differentially methylated CGIs were located outside the promoters (within the gene bodies or intergenically located) with a 2-fold increase in the number of differentially methylated CGIs located within the promoters between NBM and AML (Dataset S1, S2, S3, S4). In order to identify which genomic feature is the strongest predictor, we performed a two-dimensional cluster analysis (Figs. 2A–D) and a pair-wise comparison (Fig. S3) that indicated, of the 4 genomic features, the CGIs could cluster all the AML samples most tightly and discriminate them from NBM. In order to estimate the strength of this clustering, we have applied a prediction strength algorithm [17], which indicated very high stability of AML and NBM clusters (Fig. S4). Moreover, the examination of the differentially methylated promoters (Dataset S1) identified some previously noted targets for epigenetic silencing or alteration in hematological disorders. For example, *ID4* has been shown to act as a putative tumour suppressor gene in AML [18], *DCC* is hypermethylated in follicular lymphoma [19] and mutation of *TERT* increases the risk of familial AML [20]. This gene list also included genes that have roles in a variety of other cancers e.g. *DPP6* is down regulated in melanoma [21], *SPHKAP* plays a role in the sphingosine phosphorylation pathway that induces tumor progression and invasion [22], [23]. Additionally, differentially methylated gene bodies, intragenic CGIs (within promoters/gene bodies) and intragenic CGI shores identified a number of genes belonging to potentially important transcription factor families e.g. *MYOD1, SOX14, FOXA2, FOXB2, RUNX1* and *PAX1*.

We included AML patients with 4 cytogenetic subtypes so that we could detect DMRs that would discriminate between each subtype. The highest number of DMRs separating an AML subtype from the rest of groups was found in t(8;21) AML. By contrast, trisomy 8 AML showed the lowest number of identified DMRs. Most of total DMRs in each AML subtype were hypermethylated (∼60% of total DMRs) except in trisomy 8 AML where only 40% of total identified DMRs were hypermethylated. For all AML subtypes, the hypermethylated DMRs was located mostly in CGIs where the preferential methylation was found in CGIs located outside the promoters (within the gene bodies or intergenically located) (Table S5). Additionally, there were very few DMRs that overlapped between AML subtypes with no common hypo or hypermethylated DMRs between the all 4 AML subtypes (Fig. S5). Most of those DMRs are unique for each AML subtype i.e. DMRs associated with *MEIS1/2*, *TOP3B*, *CDH13, ST6GAL2* in t(8;12) AML, *DOK6, NCOR2* in t(15;17) AML, *ELK1*, *VMO1* in NK AML, *SNX16*, *HHEX* in trisomy 8 AML. Based on a two-dimensional cluster analysis (Figs. 2E–H) and a pair-wise comparison (Fig. S6) AML subtypes could be readily distinguished using the identified genomic features with a notable clustering of t(8;21) subgroup distantly from the other AMLs.

### Distinctive pattern of repeat sequence methylation in AML

**Figure 3:**
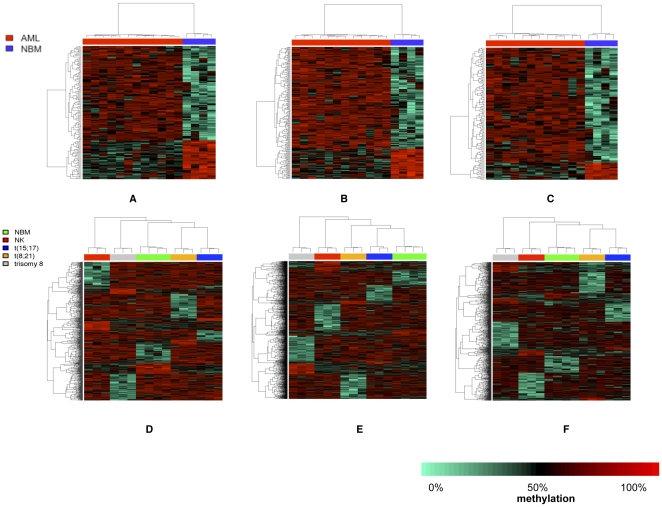
Hierarchical clustering of AML versus NBM in the interspersed repeats. In each figure, each column represents AML patient/NBM and each row represents a single DMR. First row represents cluster analysis of all AMLs versus all NBMs and the second row represents cluster analysis of AML subtypes in SINEs (A, D), LINEs (B, E) and LTRs (C, F). Distinctive hypomethylated SINEs, LINEs and LTRs clearly distinguished each AML subtype (second row).

A considerable advantage of high-throughput sequencing is the ability to investigate repeated elements, which would cross-hybridize on a microarray chip [24]. The uniquely mapped reads were used to determine the methylation patterns on repeated sequences [12], [25]. We identified numbers of interspersed elements associated DMRs between AML and NBM (Table S6, Dataset S5, S6, S7). The methylation pattern of the selected SINEs, LINEs and LTRs could readily discriminate AML from NBM (Figs. 3A–C, Fig. S7). The clearest distinction between AML and NBM was obtained with SINE methylation; 62% of those SINEs were of the Alu class (43% AluJb, 40% AluSx). These discriminating repeats had a significantly high CpG density compared with the rest of the Alu subfamilies (P = 0.002) and with the rest of SINEs (P<0.0001). An examination of the cytogenetic subtype specific repeat sequence methylation is shown in Figs. 3D–F & Fig. S8. It was evident that for LINEs, SINEs and LTRs the feature that discriminated AML subgroups was hypomethylation of particular groups of repeats. Most of those distinctive hypomethylated repeats were intergenic in LTRs and in LINEs (∼65% found in LINE1). However, most of the distinctive hypomethylated SINEs belonged to the Alu family and were intragenically located. For satellites, few repeats were found differentiating AML from NBM and discriminating between AML subtypes.

### MeDIP-seq data validation

MeDIP-seq data were validated by 2 approaches: genome-wide using HumanMethylation 27 BeadArray (Fig. S2) and also by locus-specific methods on selected DMRs. For locus-specific validation, we performed direct bisulfite sequencing of 4 DMRs on patient DNA samples; 3 DMRs were hypermethylated in AML versus NBM and one DMR was hypomethylated in AML versus NBM. The bisulfite results confirmed the MeDIP-seq data for each region (Fig. S9, Table S7). In addition, we carried out bisulfite pyrosequencing in 63 AML patients with different cytogenetic features, 7 AML cell lines [Kasumi (in duplicates), OCI-AML2, CTS, HL60, Kmoe2, P31/FUJ, THPI] and 5 NBMs. Pyrosequencing validation was performed on 2 DMRs discriminating between AML and NBM (*DPP6*, *SPHKAP*), 2 DMRs that were hypermethylated in 2 AML subtypes; *ST6GAL2* in t(8;21), *HHEX* in trisomy 8 AML and an AluJb repeat that was differentially hypomethylated in t(8;21) AML. The results revealed statistically significant differences in DNA methylation as was detected by MeDIP-seq (Kruskal-Wallis test followed by Dunn's multiple comparison P<0.05) (Fig. S10, Table S8).

### Regional DNA methylation and gene expression

**Figure 4:**
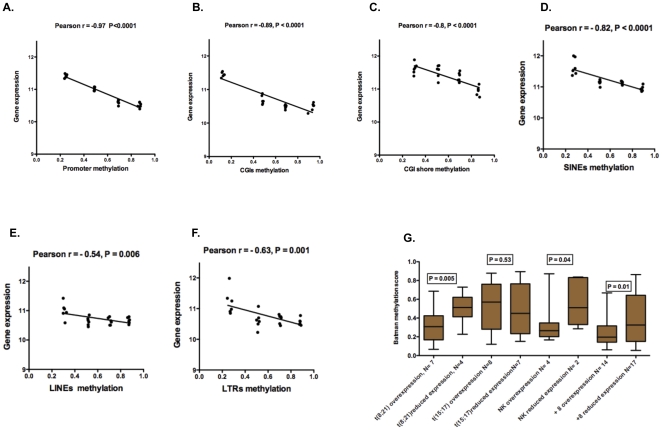
Correlation between DNA methylation and gene expression. (A–F) For a single AML patient we categorized the gene methylation into 4 groups (>0.4, 0.4–0.6, 0.6–0.8, >0.8 Batman scores). We correlated the average of each methylation group to corresponding average of gene expression. (G) Box plots of DNA methylation levels of over- and under-expressed genes in each triplicate of t(8;21), t(15;17), NK and trisomy 8 AML subtypes. N refers to the number of genes in each set. Mann Whitney test of the two sets of genes demonstrated a significant methylation difference between the medians in t(8;21), NK and trisomy 8 AML subtypes.

We sought to determine the relationship between the methylation profiles and gene expression in AML patients. Therefore, an array based gene expression profiling for 6 of the AML samples that had available RNA was performed. Correlating the gene expression to corresponding DNA methylation on an average scale revealed strong significant inverse correlation in promoters (13,690 genes) (Pearson r = −0.97, P<0.0001), CGIs (inside the promoters of 8,745 genes) (Pearson r = −0.89, P<0.0001) and their parallel CGI shores (2 Kb upstream to the transcriptional start site; TSS) [26] (Pearson r = −0.8, P<0.0001) (Figs. 4A–C). The intragenic interspersed repeats (within the promoters/gene bodies) showed also significant inverse correlation with gene expression. SINEs (1,285 intragenic repeats) showed the strongest negative correlation (Pearson r = −0.82, P<0.0001) followed by LTRs (541 intragenic repeats) (Pearson r = −0.63, P = 0.001) and finally LINEs (11,242 intragenic repeats) (Pearson r = −0.54, P = 0.006) (Figs. 4D–F). This analysis revealed that the gene expression was strongly correlated with DNA methylation of the promoters, which is consistent with the accepted role of DNA methylation around TSS on related gene expression [27], [28], [29], [30]. However, this does not exclude the importance of some individual CGIs located outside the promoters in altering nearby gene expression [31], [32].

Furthermore, we investigated the relationship between differential gene expression and DNA methylation by integrating our promoter MeDIP-seq data with published gene expression data [33], [34], [35]. We questioned whether promoter DNA methylation was associated with the distinctive over and under expressed genes in each AML subtype. There was significant promoter DNA methylation difference between the distinctive expressed genes in 3 AML subtypes; t(8;21) AML, NK AML and trisomy 8 AML (Mann Whitney test, P = 0.005, P = 0.04, P = 0.01 respectively) (Fig. 4G, Tables S9, S10). This indicates that differential gene expression was correlated with differential promoter DNA methylation in most AML subtypes. It was notable in this analysis that the t(15;17) subgroup did not exhibit this correlation suggesting additional modifying factors may affect gene expression in this subgroup.

### Down regulation of candidate methylated genes in AML and AML subtypes

**Figure 5:**
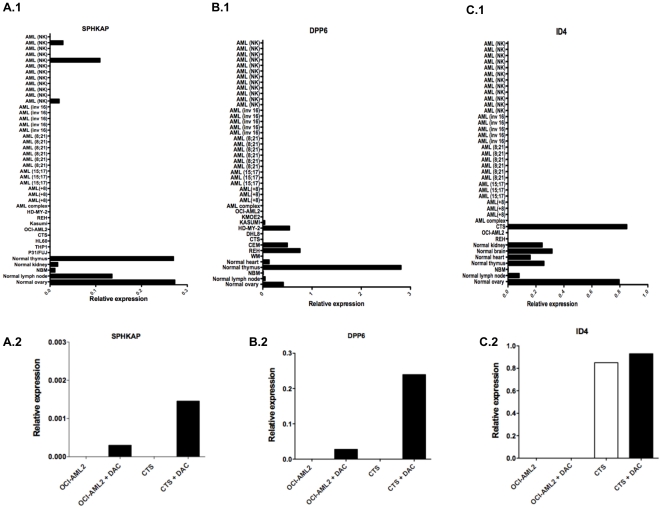
*SPHKAP*, *DPP6* and *ID4* gene expression in AML. (A.1, B.1, C.1) Relative expression of *SPHKAP*, *DPP6*, *ID4* (respectively) in AML and normal tissues. The genes were down regulated in AML patients and in cancer cell lines, while the genes were up regulated in normal tissues. (A.2, B.2, C.2) Relative expression of *SPHKAP*, *DPP6* and *ID4* (respectively) in OCI-AML2 and CTS cell lines before and after treatment by DAC. Gene expression was restored in most of cell lines treated by DAC.

To investigate the consequence of promoter methylation, the expression of 3 of the genes with the most consistent methylated promoters in AML was performed i.e. *DPP6* (absolute methylation difference = 0.46, P = 0.0007), *SPHKAP* (absolute methylation difference = 0.45, P = 0.0002) and *ID4* (absolute methylation difference = 0.28, P = 0.003). The analysis was performed in 30 AML patients (including 4 AML patients who were previously involved in MeDIP-seq experiment), cancer cell lines and normal tissues using real time RT-PCR. The results showed that *DPP6, SPHKAP* and *ID4* were down regulated in AML patients (Figs. 5A.1, B.1, C.1). By contrast, *DPP6, SPHKAP* and *ID4* were expressed in normal tissues. In addition, *SPHKAP* was down regulated in cancer cell lines investigated, while the expression of *DPP6* and *ID4* was variable among cancer cell lines. Next, we tested the effect of a demethylating drug [DAC (5-aza-2′-deoxycytidine)] on the expression of both *SPHKAP* and *DPP6* genes in 2 AML cell lines (OCI-AML2 and CTS). This confirmed that the demethylating treatment was able to restore the expression of both genes (Figs. 5A.2, B.2).

Since, the *HHEX* gene (also known as *PRH* gene) showed a significant methylated CGI located in its gene body (between exon 2 and exon 3) in trisomy 8 AML against the rest of the groups (absolute methylation difference = 0.74, P = 9×10^−6^) (Dataset S3), we validated the DNA methylation of that island by pyrosequencing (Fig. S10d). This demonstrated a significant methylation difference between trisomy 8 AML and t(8;21) AML (Kruskal-Wallis test followed Dunn's multiple comparison tests P<0.5) (Fig. S11a). Next, we investigated *HHEX* gene expression by RT-PCR. *HHEX* gene showed a significant expression difference among AML subtypes with a significant expression difference being detected between trisomy 8 AML and t(8;21) AML (Kruskal-Wallis test followed Dunn's multiple comparison tests P<0.5) (Fig. S11b). Correlating the pyrosequencing results with *HHEX* gene expression showed a moderate but significant inverse correlation between the identified CGI methylation and related gene expression (Spearman r = −0.5, P = 0.004). Treating CTS and OCI-AML2 cell lines with DAC showed an increase in *HHEX* gene expression (Fig. S11c). Notably, from MeDIP-seq results, there was no DMR identified among AML subtypes in the promoter of *HHEX* gene; *HHEX* promoter methylation was less than 0.2 Batman score in all MeDIP-seq samples including trisomy 8 AML.

## Discussion

We have used MeDIP-seq to establish the global methylome for AML. The comprehensive nature of this study (12 independent primary tumors) has allowed us to investigate the potential epigenomic role of not only gene promoters but also genomic elements, including CGIs and repeated elements. It is possible to draw some general conclusions from this study. Firstly, comparison of the whole genome methylation of the leukemia with the controls showed that leukemic DNA was only 2.7% less methylated. This contrasts with the generally held view that cancer is characterized by global hypomethylation amounting to a 10%–20% difference [14], [36]. It may be that this limited global hypomethylation is a particular feature of AML. However, a recent MeDIP-seq study of pooled DNA from peripheral nerve sheath tumors also indicated a global DNA methylation change of only 0.7% in malignancy compared to normal [12]. A second general conclusion is that leukemia specific methylation extends into regions that are not specifically associated with gene promoters. We similarly found that AML subtype specific methylation extended beyond gene promoters and encompassed other genomic features.

Previous genome wide epigenetic studies in leukemia have, for technical reasons, focused on gene promoters and/or CGIs [37], [38]. Our identification of AML subtype methylation patterns effectively confirms previous observations [38] that convincingly demonstrated AML subtype specific methylation of gene promoters. It was of interest to compare our MeDIP-seq results for the t(8;21) and t(15;17) AMLs to those obtained in that study [38]. We found 48 genes with the same methylation status observed by Figueroa and colleagues who used a different technique; (HELP technique: HpaII tiny fragment Enrichment by Ligation-mediated PCR) [39]) (Tables S11, S12). Despite the methodological differences between the two studies it was significant that a common list of genes could be readily identified.

Although our study did not include all possible subtypes of AML it did robustly identify specific methylation targets that were confirmed in a larger series of AML samples using pyrosequencing technology. Amongst the novel targets identified in this study the frequent methylation of the promoter of the gene (*SPHKAP*) encoding sphingosine kinase anchoring protein in AML was of particular interest since *SPHKAP* was down regulated in both AML patients and cancer cell lines. The *SPHKAP* protein was identified through its interaction with and regulation of *SPHK1* activity [22]. Additionally, *SPHKAP* (or *SKIP*; *SPHK1*-interactor protein) was recently identified as a member of A-kinase-anchoring proteins (AKAPs) [40]. *SPHK1* catalyses the phosphorylation of sphingosine to sphingosine-1-phosphate (S1P) which promotes cell survival and proliferation [23]. *SPHK1* is overexpressed in a range of cancers and has been proposed as a novel target for cancer therapeutics [41]. The possible leukemogenic role of loss of expression of *SPHKAP* and resultant effects on the lipid signaling pathways remains unclear. However, it is of interest to note that expression of the *RUNX1* gene, which is involved in the t(8;21) translocation in AML, has recently been linked to regulation of key enzymes involved in sphingolipid metabolism [42].

The second novel promoter associated DMR, which appeared in our study to be down regulated in AML patients, was the *DPP6* gene. The *DPP6* gene was reported as a hypomethylated gene in colon cancer [26] and at the same time is considered as a biomarker for melanoma [21]. *DPP6* gene became hypermethylated in some tumors and hypomethylated in other types of tumors as the promoter hypomethylation is important in activation some oncogenes and in provoking loss of imprinting (LOI) [43].

Since up to 45% of the human genome consists of repetitive sequences which are not analyzed by array-based methods, it was desirable to extend the analysis to include such sequences. Our study suggests that both the hyper- and hypo-methylation of individual members of the LINE, SINE and LTR families can readily discriminate AML from NBM. Although we found some members of the satellite repeat family exhibiting differential methylation, the discrimination was much less clear. Differences between satellite methylation and interspersed repeat methylation were previously reported in leukemia and in bladder cancer [44]. It was also possible to identify repeat family members that exhibited differential methylation between AML subtypes. As indicated, very distinct patterns of hypomethylation of members of the LINE, SINE and LTR appear to be associated with each AML subtype. Many studies have identified hypomethylation of repeat sequence elements as important in cancer [36], [43]. For example, hypomethylation of LINEs has been observed in several cancer types and appears to increase with the degree of malignancy [45]. Hypomethylation of LINEs has also been associated with the phases of chronic myeloid leukemia and shown to have prognostic value [46]. This novel observation of AML subtype specific hypomethylation highlights the potential role of SINEs, LINEs and LTRs in the transcription activation [47], [48] of genes important in cancer progression and speciation [46], [49]. In addition, these regions could represent discriminating biomarkers valuable in AML diagnostics.

The establishment of such high resolution AML methylomes not only reveals the subtle epigenetic changes involved in leukemogenesis but also has potentially important clinical implications. It has been shown that detection of methylated sequences in clinical remission for AML has the power to predict relapse risk for those patients [50]. This study has therefore identified a large number of potential biomarkers that, in principle, could be used to predict relapse in AML with even greater statistical power.

## Materials and Methods

### Clinical samples

We applied MeDIP-seq to 12 AML patients. Diagnosis of leukemia was based on clinical and morphological features [51]. Median age of AML patients was 38.5 and median blast percentage was 81% (full details Table S1). Patients' samples (peripheral blood or bone marrow) were stored in the tissue bank of the St Bartholomew's Hospital after informed written consent was obtained. The human Kasumi leukemic cell line (ACC: 220), OCI-AML2 leukemic cell line (ACC: 99), HL60 leukemic cell line (ACC: 3), Kmoe2 leukemic cell line (ACC: 37), REH leukemic cell line (ACC: 22) were obtained from the Deutsche Sammlung von Mikroorganismen und Zellkulturen - DSMZ - (German Collection of Microorganisms and Cell Cultures). Human CTS leukemic cell line [52], THPI leukemic cell line [53] and P31/FUJ leukemic cell line [54], CEM leukemic cell line [55], HD-MY-Z lymphoblastic cell line [56], DHL lymphoma cell line [57] and WM melanoma cell line [58] were kindly provided by Dr. Simone Jueliger (Queen Mary University of London, London, UK). DNA was extracted using Qiagen DNeasy Blood & Tissue kit according to manufacturer's instructions. The ethical approval to access the stored material and to carry out the study was obtained from East London and City Research Ethics Committee (ref 10/H0704/65).

### MeDIP-seq

Only high quality genomic DNA was subjected to MeDIP-seq protocol (Text S1) [25], [59]. This is based on using a monoclonal antibody against 5-methylcytosine of previously sonicated DNA. MeDIP DNA libraries were quantified using RT-PCR and the Agilent Bioanalyser 2100 to get 10 nanomolar concentration libraries. The MeDIP libraries were subjected to high-throughput 45 base paired-end sequencing using Illumina Genome Analyzer (GA-II) [60]. Two different algorithms were used for the alignment of the generated reads against the reference human genome (NCBI 36/hg18); Maq (Mapping and Assembly with Qualities) [61] (http://maq.sf.net/ and Li et al.) and Bowtie [62]. Following the alignment, the repeated sequences (including PCR duplicates and reads that mapped to more than one location on the genome) were filtered from the data. Reads with Maq score of <10 were excluded from subsequent analysis. Bowtie rounded the quality values to the nearest 10. For methylation analysis of the uniquely aligned reads, the Batman algorithm has been used [11]. Batman (A Bayesian Tool for Methylation Analysis) algorithm infers the absolute methylation state for 100 bp windows by estimating local sequencing read enrichment for methylation taking into account the varying densities of methylated CpGs across the genome [11]. Batman output is in the form of GFF format, each GFF file represents a score that is equals the median of methylation states in a 100 bp window. The score ranges between 0–1 according to the level of methylation. The sequencing data are available in GEO, accession number: GSE28314

### MeDIP-seq data statistical workflow

Quantile normalization was performed to reduce the possible variations among the laboratory assays and to facilitate the comparison of the genes across all the samples. This method is based upon the concept of quantile-quantile plot extended to n dimensions (where n is the number of samples) [63]. In order to reduce the complexity of the normalized data, we excluded the genes in which the difference between the maximum and the minimum methylation values is lower than the mean of the standard deviation values of this genomic feature across all the samples. We next used empirical Bayes statistics provided by Bioconductor's limma R package to select the top discriminating genes for each feature [64]. This was performed for every AML cytogenetic group against other groups including NBM, and for all AML versus all NBM. The empirical Bayes model was used to compute moderated t-statistics and F-statistic. The Benjamini and Hochberg false discovery rate (FDR) was used as a multiple testing correction generating adjusted P value for each gene. However, due to small sample size, not many genes remained statistically significant when multiple testing corrections were applied. The frequency of uncorrected P values<0.05 for differential methylation was higher than expected assuming a random distribution. This uncorrected P value distribution indicates a differential methylation pattern between specified groups (Fig. S12) [65].

### DMR

DMR was defined as a differential methylated region with uncorrected P<0.05 and an absolute methylation difference >0.25 Batman score (equal to at least 25% difference in DNA methylation). This threshold was defined using the distribution of absolute differences in methylation (Batman) scores of all covered genomic features (618,556) between normal and leukemic samples (Fig. S13). Since the 99th percentile of the differences in methylation scores corresponds to 0.23, the minimal difference in methylation between groups required for calling DMRs was rounded to 25%. In order to estimate FDR in the DMR calling, a mixture model approach was used [66]. DMR identification based on P value<0.05 and absolute methylation difference >25% gave an FDR of 2.4% across investigated genomic features and repeats, that was comparable to results obtained by a recent MeDIP-seq study [12].

### Cluster analysis

Hierarchical clustering and pair-wise comparison were formed using Pearson correlation coefficient to construct the distance matrix among samples together with ‘Ward’ linkage clustering method [67]. In order to reduce the data for clustering purposes, we selected DMRs with P value<0.001 for which we noticed particular enrichment (Fig. S13).

### Whole genome analysis

For the comparison of the whole genome methylation between AML and NBM, we have categorized DNA methylation into 5 groups and used Fisher's exact test provided with R to investigate if there was a statistically significant difference in the global DNA methylation [64].

### Repeats

Repeated regions were obtained from nested RepeatsRM327 table downloaded from UCSC genome annotation database (http://hgdownload.cse.ucsc.edu/goldenPath/hg18/database/).

### Direct Bisulfite sequencing

500 ng genomic DNA was bisulfite converted using EZ DNA methylation ™ kit (Zymo research). PCR amplification of the bisulfite converted DNA was performed through 42 cycles at 55°C annealing temperature, the primers used for the PCR are provided in Table S13. Amplified products were cloned using the TOPO TA cloning (Invitrogen, TOPO TA cloning kit) and 5–10 clones were picked for template-amplification of DNA and further sequencing. Only >95% bisulfite converted clones were analyzed using QUMA (quantitative method for methylation analysis, http://qmua.cdb.riken.jp).

### Bisulfite pyrosequencing

500 ng genomic DNA was bisulfite converted using EZ-96 DNA kit TM (Zymo research) specified by the manufacturer. The converted DNA was PCR amplified using primers for each set of genes; the primers were designed using PyroMark Assay design 2.0 (primers provided in Table S14). The pyrosequencing was performed according to a published protocol [68] using PSQ 96 MA (Qiagen) to get a percent methylation at a single CpG site. The percent methylation at each CpG was calculated using PyroQ-CpG 1.0.9.

### Illumina Infinium array

500 ng genomic DNA was bisulfite converted and hybridized onto Illumina Infinium HumanMethylation 27 BeadArrays according to the manufacturer's protocol [69]. The GenomeStudio Software was used for methylation analysis. As Illumina Infinium array confined single CpG in ∼124 bp, we determined from MeDIP-seq a 100 bp window that includes this CpG in its centre. Correlating the average Batman score with each 1% Infinium array bin revealed a strong correlation between both methods (Fig. S2).

### Array based gene expression profiling

Good quality RNA was available for 6 AML patients involved in MeDIP-seq experiment. Total RNA of AML patient samples was extracted from a total of 10–20×10^6^ thawed cells using Trizol (Gibco-BRL) purification method. The Applied Biosystems Human Genome Survey Microarray (P/N 4337467) was used for the expression profile [70]. The microarrays contain 31,700 60-mer oligonucleotide probes representing 29,098 individual human genes, and uses chemiluminescence (CL). cRNA target preparation and array hybridisation were performed according to the manufacturer's protocol (P/N 4339629). Signal intensity, S/N ratio (signal to noise ratio), and flags values for each array were extracted. If S/N was greater than 3, it was concluded that the signal detected was (confidence of 99.9%) a function of the gene expression levels detected by the probe. For those probes that have flags value above 5,000, the signals are considered missing values. The signal values were log2 transformed and normalized across arrays with quantile normalizaiton method after control probes were removed. The S/N ratio was used as filtering criteria. All the statistical analysis was performed with the statistical language R (http://www.R-project.org). The array expression data has been deposited in a MIAME compliant database GEO accession number: GSE34722, (MeDIP-seq study No. 1; GSM853941, MeDIP-seq study No. 3; GSM854020, MeDIP-seq study No. 4; GSM854034, MeDIP-seq study No. 5; GSM854005, MeDIP-seq study No. 8; GSM853976, MeDIP-seq study No. 9; GSM854019).

### RT-PCR

We carried out RT-PCR starting with total RNA (1 µg) for cDNA synthesis using random primer with Superscript III reverse transcriptase performed as per the manufacturer's instructions (Invitrogen). Maximum cDNA concentration was used in a volume of 10 µl per run. Real time was carried out using Taqman universal master mix II as per the manufacturer's instructions [71]. The maximum amount of RNA was used as per the manufacturer's instructions. Reactions were run on the ABI 7900HT fast Real time PCR using 96-well plate and the standard thermal cycler protocol with 40 cycles. Control normal adult RNA was obtained from Agilent genomics. Normal breast RNA was used to compare the results in *SPHKAP*, *DPP6* and *ID4*. Normal lymph node was run on every plate to ensure the consistency across the runs. 18s RNA acquired from ABI was used as the endogenous control. All primers were designed and generated by ABI.

### Treatment of cells with 5-aza-2′deoxycytidine (DAC)

Twenty-four hours before adding 5-aza-2′ deoxycytidine (DAC) (Sigma), 1×10^6^ AML cell line cells were maintained in culture containing RPMI-1640 with 10% FBS. Cells were treated by DAC through adding freshly prepared 5 µM DAC in dimethylsulfoxide (DMSO) to the standard media. DAC treatment was repeated at 48 hours. Control cell lines were treated identically except that they were treated with standard media to which DMSO only had been added. We assayed the cells for gene expression 72 hours after the treatment.

## Supporting Information

Text S1 **Supporting method MeDIP-seq protocol.**
(DOC)

Figure S1 **Saturation analysis of MeDIP-seq samples** (a, b, c) The saturation analysis investigates whether the number of unique reads is sufficient to generate a saturated and reproducible methylation profile of the reference genome. The higher Pearson correlation r the greater assurance of the reproducibility of the methylation profiles. Sample study number is identified in Table S1.
(DOC)

Figure S2 **Correlation between MeDIP-seq and Illumina Infinium array.** A significant positive correlation was found between MeDIP-seq and Illumina Infinium array in the three MeDIP-seq samples.
(DOC)

Figure S3 **Pair-wise comparison between AML and NBMs in 4 genomic regions.** Red colored spots indicate high similarity and white colored spots low similarity. CGIs (C) showed the highest similarities between AML subtypes and a clear discrimination from NBMs. (A) Promoters, (B) Gene bodies, (D) CGI shores.
(DOC)

Figure S4 **Estimating the number of clusters in data set consisting of differentially methylated CGIs using prediction strength method** The consecutive number of clusters is given on the x-axis. The vertical bars illustrate the standard error of the prediction strength over 5 cross-validation folds. Prediction strength above 0.8 indicates well-separated clusters. Dividing the CGI associated DMRs data set into two clusters, referring to AML and NBM groups, gives the highest stability.
(DOC)

Figure S5 **Characters of DMRs in AML subtypes.** Venn diagrams showed few overlapped DMRs between AML subtypes with no common DMRs detected between the all 4 AML subtypes.
(DOC)

Figure S6 **Pair-wise comparison between AML subtypes in 4 genomic regions.** t(8;21) AML subtype is discriminated distantly from all the other AML subtypes. (A) Promoter, (B) gene bodies, (C) CGIs, (D) CGI shores.
(DOC)

Figure S7 **Pair-wise comparison between AML and NBMs in repeat sequences.** (A) SINEs showed the highest similarities between AML subtypes among other repeats; (B) LINEs and (C) LTRs.
(DOC)

Figure S8 **Pair-wise comparison between AML subtypes in repeat sequences.** There was clear discrimination between AML subtypes in (A) SINEs, (B) LINEs and (C) LTRs.
(DOC)

Figure S9 **Direct bisulfite sequencing of significant differentially methylated genes/genomic regions in MeDIP-seq samples.** (a, b, c, d, e) For all figures, the horizontal line represents the position of each CpG investigated and the vertical line is the percentage of the methylation at particular CpG site from 0–100%. The analysis was performed using QUMA.
(DOC)

Figure S10 **Pyrosequencing results of candidate genomic regions in AML patients, AML cell lines and NBMs.** (a) *SPHKAP*, (b) *DPP6*, (c) *ST6GAL2*, (d) *HHEX* and (e) Alu repeat. N refers to the number of samples tested for each investigated genomic region. Kruskal-Wallis test showed significant methylation difference among the groups (P<0.0001) for all tested genes and repeat. Dunn's multiple comparison tests showed that there was significant methylation difference between AML patients and NBMs in *SPHKAP* and *DPP6* (P<0.05). Also, there was significant methylation difference between AML samples and AML cell lines in all investigated genes (P<0.05) except in the repeat.
(DOC)

Figure S11 ***HHEX*** **gene methylation and expression assay.** (a) *HHEX* gene methylation (a significant methylated CGI located within the body of *HHEX* gene) among different AML patients. (b) *HHEX* gene expression among different AML patients. (c) Effect of DAC on *HHEX* expression in AML cell lines. *HHEX* gene expression was measured relative to NBM, PB = peripheral blood from healthy donors.
(DOC)

Figure S12 **Histograms of uncorrected P values after testing the equality of the methylation means between groups.** (a) in 4 genomic regions and (b) in repeats. When investigating the data with equal means between groups, the P values were expected to be uniformly distributed across the unit interval (blue line). Comparing the distribution of uncorrected P values to the uniform distribution expected for random data revealed enrichment of P value<0.05 (red line) indicating differential methylation pattern. Satellites did not show a specific distribution of uncorrected P values across the samples. High frequencies of P values<0.05 across the samples were observed in the other tested repeats; SINEs, LINEs and LTRs.
(DOC)

Figure S13 **Histogram illustrating the distribution of uncorrected P values after testing equality of methylation between normal and leukemic samples for all genomic features and repeats.** For random data the distribution is expected to be uniformly distributed across the unit interval (blue horizontal line). The frequency of P values<0.05 (red line) is higher than expected with particular enrichment of P values<0.001.
(DOC)

Table S1 **Patients and control samples.**
(DOC)

Table S2 **Criteria of reads generated from Illumina GAII,**
(DOC)

Table S3 **Description of the genomic regions from MeDIP-seq results.**
(DOC)

Table S4 **DMRs identified in AML versus NBM in 4 genomic regions.**
(DOC)

Table S5 **DMRs identified in AML subtypes in 4 genomic regions.**
(DOC)

Table S6 **DMRs associated with repeats**. (a, b) AML versus NBM, (c) between AML subtypes.
(DOC)

Table S7 **Direct bisulfite sequencing validation of selected genomic regions.**
(DOC)

Table S8 **Pyrosequencing validation of selected genomic regions. (a) AML versus NBM, (b) in AML subtypes.**
(DOC)

Table S9 **DNA methylation of over- and under expressed genes in t(8;21), t(15;17) & NK AML subgroups that were included in MeDIP-seq experiment.**
(DOC)

Table S10 **DNA methylation of over- and under expressed genes in trisomy 8 AML subgroup that was included in MeDIP-seq experiment.**
(DOC)

Table S11 **The overlapped genes between the results of MeDIP-seq study and array-based study for t(15;17) AML.**
(DOC)

Table S12 **The overlapped genes between the results of MeDIP-seq study and array-based study for t(8;21) AML.**
(DOC)

Table S13 **Direct bisulfite sequencing primers.**
(DOC)

Table S14 **Pyrosequencing primers.**
(DOC)

Dataset S1 **Differentially methylated promoters.**
(XLS)

Dataset S2 **Differentially methylated gene bodies.**
(XLS)

Dataset S3 **Differentially methylated CGIs.**
(XLS)

Dataset S4 **Differentially methylated CGI shores.**
(XLS)

Dataset S5 **Differentially methylated SINEs.**
(XLS)

Dataset S6 **Differentially methylated LINEs.**
(XLS)

Dataset S7 **Differentially methylated LTRs.**
(XLS)
