# Supplementary material for: Genome Wide Analysis of Acute Myeloid Leukemia Reveal Leukemia Specific Methylome and Subtype Specific Hypomethylation of Repeats
Source: PLoS One. 2012 Mar 29;7(3):e33213. doi: 10.1371/journal.pone.0033213 (PMC3315563; doi:10.1371/journal.pone.0033213)
Supplement: Text S1 — Supporting method MeDIP-seq protocol. (DOC) [file pone.0033213.s001.doc]

**Supporting method**

**MeDIP-seq protocol**

5μg of sheared DNA are blunt-ended by incubation for 30 minutes at 20°C in 100µl reaction volume containing 10µl T4DNA polymerase to fill in 5´overhangs, 1µl Klenow polymerase to remove 3´ overhangs, 5µl T4 polynucleotide kinase to phosphorylate the 5´-OH, 10µl T4 DNA ligase buffer with 10mM ATP and 4µl of dNTPs. The reaction volume is purified using QIAquick PCR purification kit and eluted in 32µl elution buffer (EB). In order to allow ligation to the adaptors, 10µl adenine is added to the 3´ end of DNA fragment by 3µl Klenow exo-minus in the presence of 3µl of Klenow buffer. The reaction mix is incubated at 37°C for 30 minutes and then purified by MinElute PCR purification kit in 10µl EB. 10µl adaptors are added to the purified DNA in the presence of 5µl DNA ligase and 25µl DNA ligase buffer. The reaction volume is incubated for 15 minutes at room temperature and cleaned by QIAquick PCR purification kit in 50 µl EB. 50 ng of ligated purified DNA is set aside as an input fraction and at least 4mg of the same DNA is required for immunoprecipitation (IP). 4μg of ligated DNA will be diluted with water to reach a final volume 250µl. For proper IP reaction, genomic DNA is denaturated by heating at 100°C for 10 minutes and cooled immediately on ice for 10 minutes. The reason for DNA denaturation is that the IP is more efficient on single stranded DNA (ssDNA) than double stranded DNA (dsDNA). 250ml 2X IP buffer is added to the denaturated DNA sample together with 7.5µl anti-5methyl cytosine antibody (Diagenode) and incubated for 2 hours at 4 °C with slow rotation. 40 µl of magnetic Dynabeads (Dynabeads M-280 sheep anti-Mouse IgG, Invitrogen) are prewashed with 1ml 1XIP buffer and collected with a magnetic rack. Dynabeads are added to the DNA sample and incubated for 2 hours at 4 °C with slow rotation. Dynabeads are collected using a magnet and washed with 750µl 1X IP buffer for three times. To release the immunoprecipitated DNA, Dynabeads are resuspended in 200µl digestion buffer and 5 µl of Proteinase Kinase (10mg/ml). The suspension is incubated for 2 hours at 55°C with shaking to avoid the sedimentation of the beads. After the incubation, the sample is divided in 2 separate tubes and purified by Zymo DNA Clean and Concentrator-5 kit (using 700 µl binding buffer) in 20 µl EB. 10 µl MeDIP and 10 ng of input DNA are subjected to limited PCR (LM PCR) using primers provided from Illumina for paired end library preparation. LM PCR are done duplicate for MeDIP and single for the input. After the amplification, the MeDIP PCR tubes are combined together and purified in 20µl EB by Zymo DNA Clean and Concentrator-5 kit. Real time PCR was done to validate the enrichment level of DNA after IP by calculating the ΔΔ CT of methylated and unmethylated genes in MeDIP and the input after being normalized against a reference gene. For size selection, 15 µl of MeDIP is run over 2% low melting temperature gel. MeDIP is excised from gel in the range of 250-300 bp and eluted in 30 µl EB using Qiagen Gel Extraction kit.
