# Supplementary material for: Genome Wide Analysis of Acute Myeloid Leukemia Reveal Leukemia Specific Methylome and Subtype Specific Hypomethylation of Repeats
Source: PLoS One. 2012 Mar 29;7(3):e33213. doi: 10.1371/journal.pone.0033213 (PMC3315563; doi:10.1371/journal.pone.0033213)

**Figure S1.** **Saturation analysis of MeDIP-seq samples**

(a, b, c) The saturation analysis investigates whether the number of unique reads is sufficient to generate a saturated and reproducible methylation profile of the reference genome. The higher Pearson correlation r the greater assurance of the reproducibility of the methylation proﬁles. Sample study number is identified in Table S1.

a.


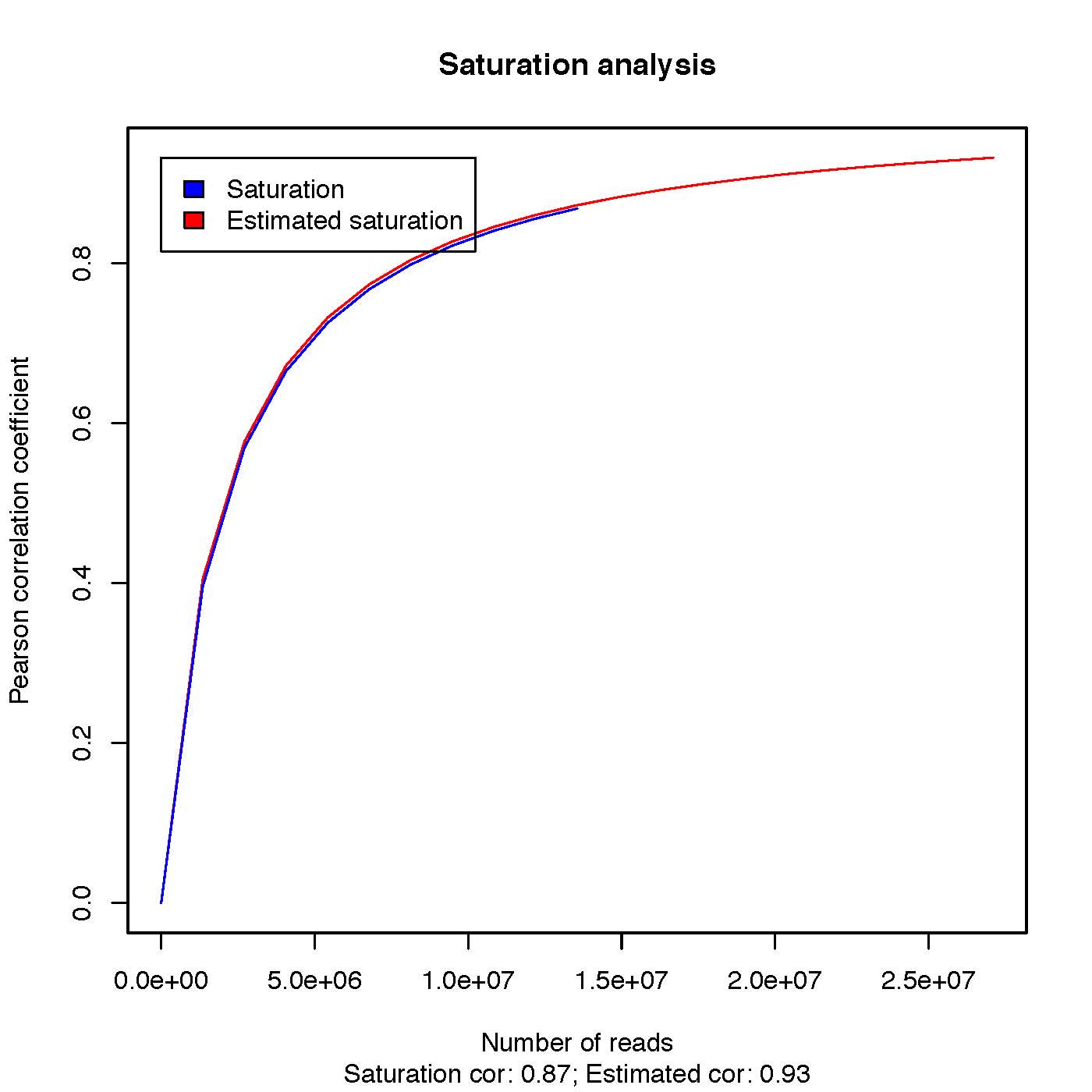

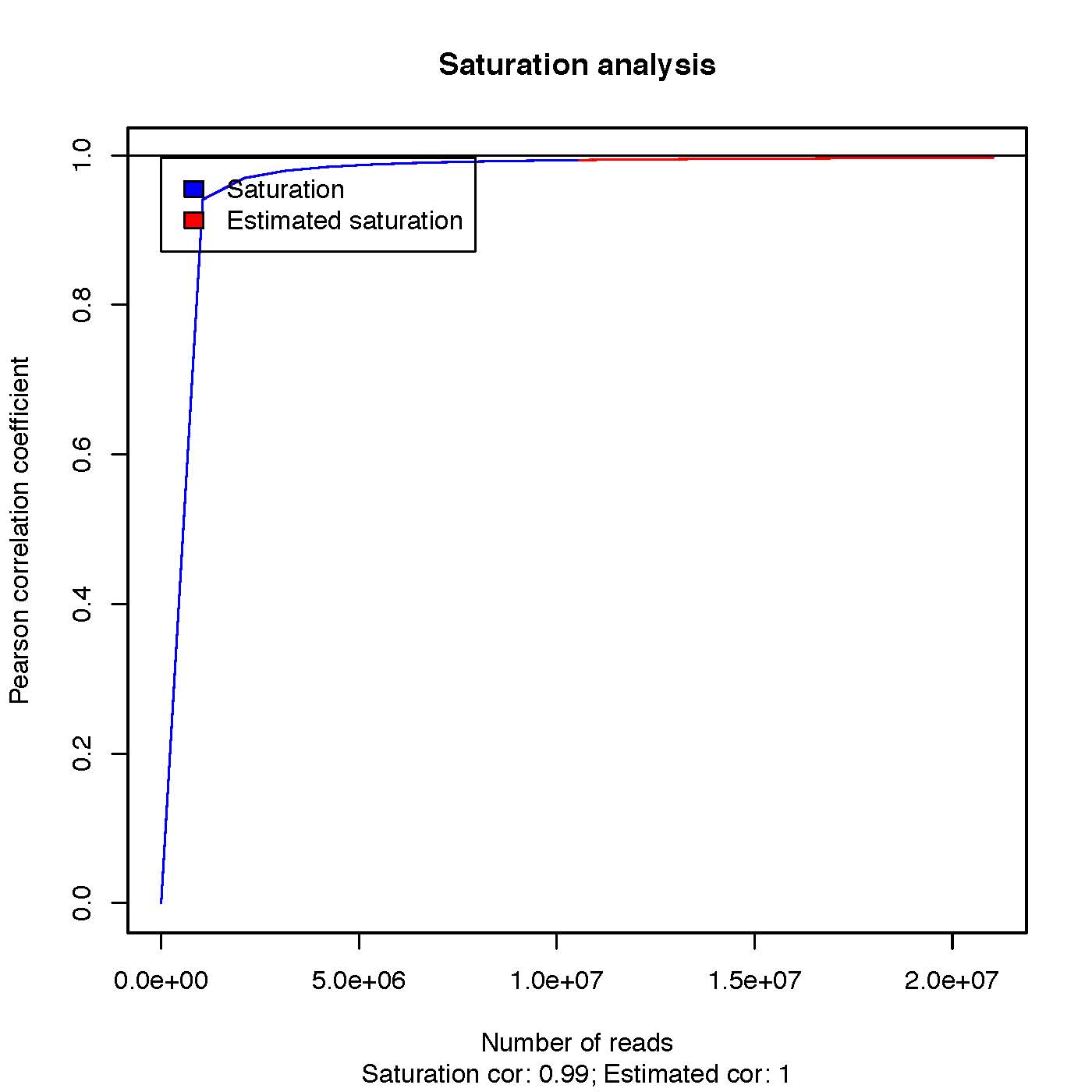


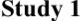

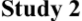


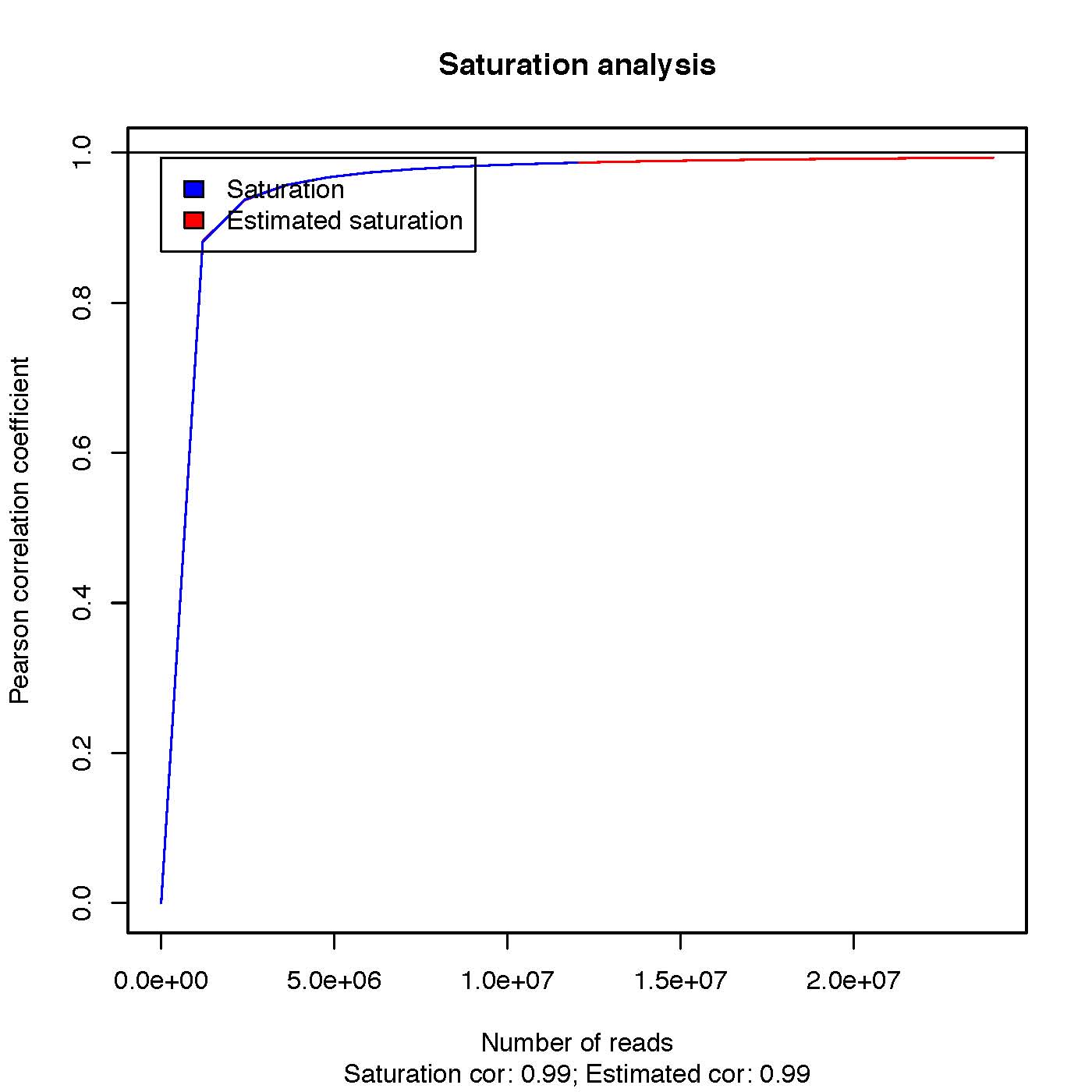

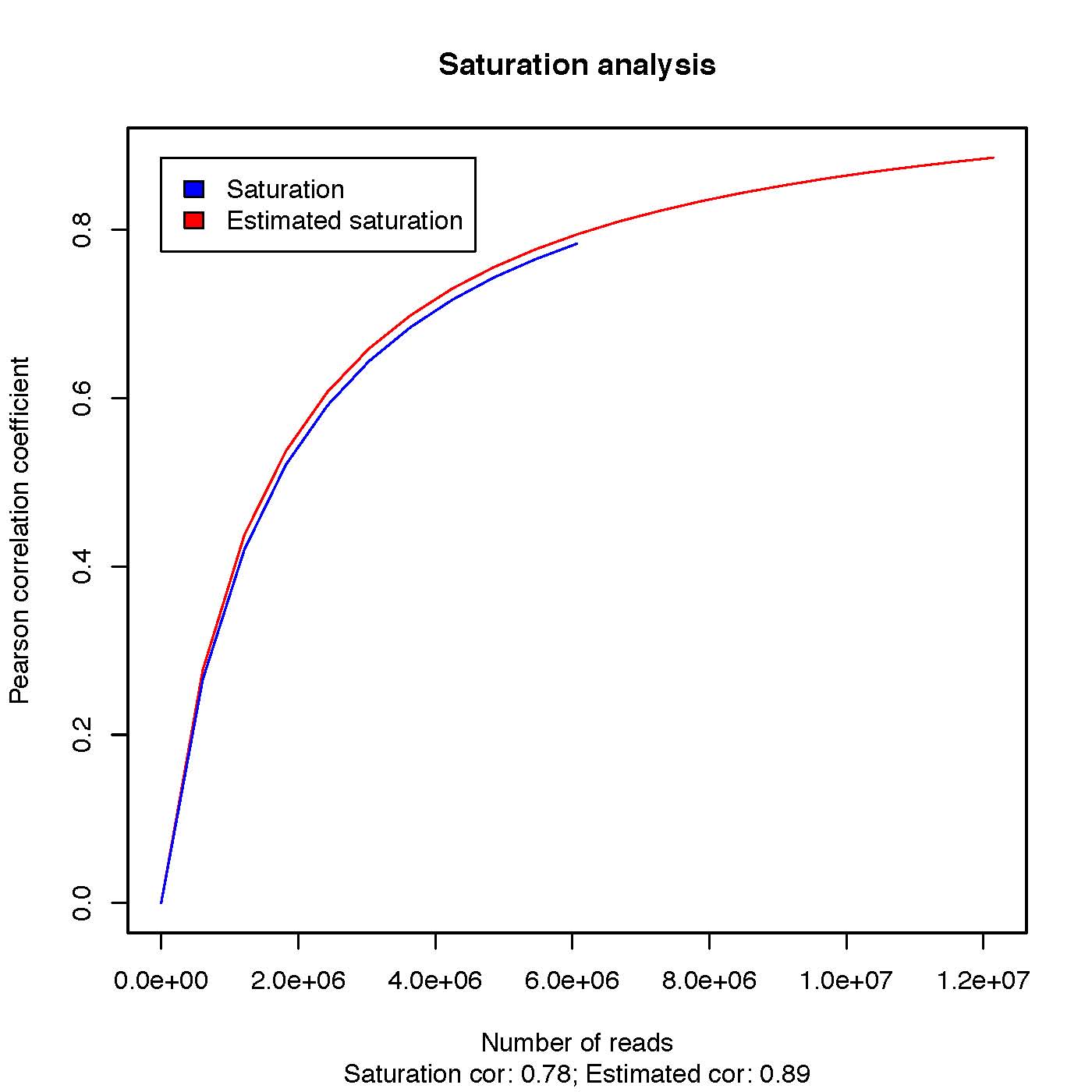


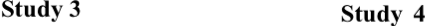


b.


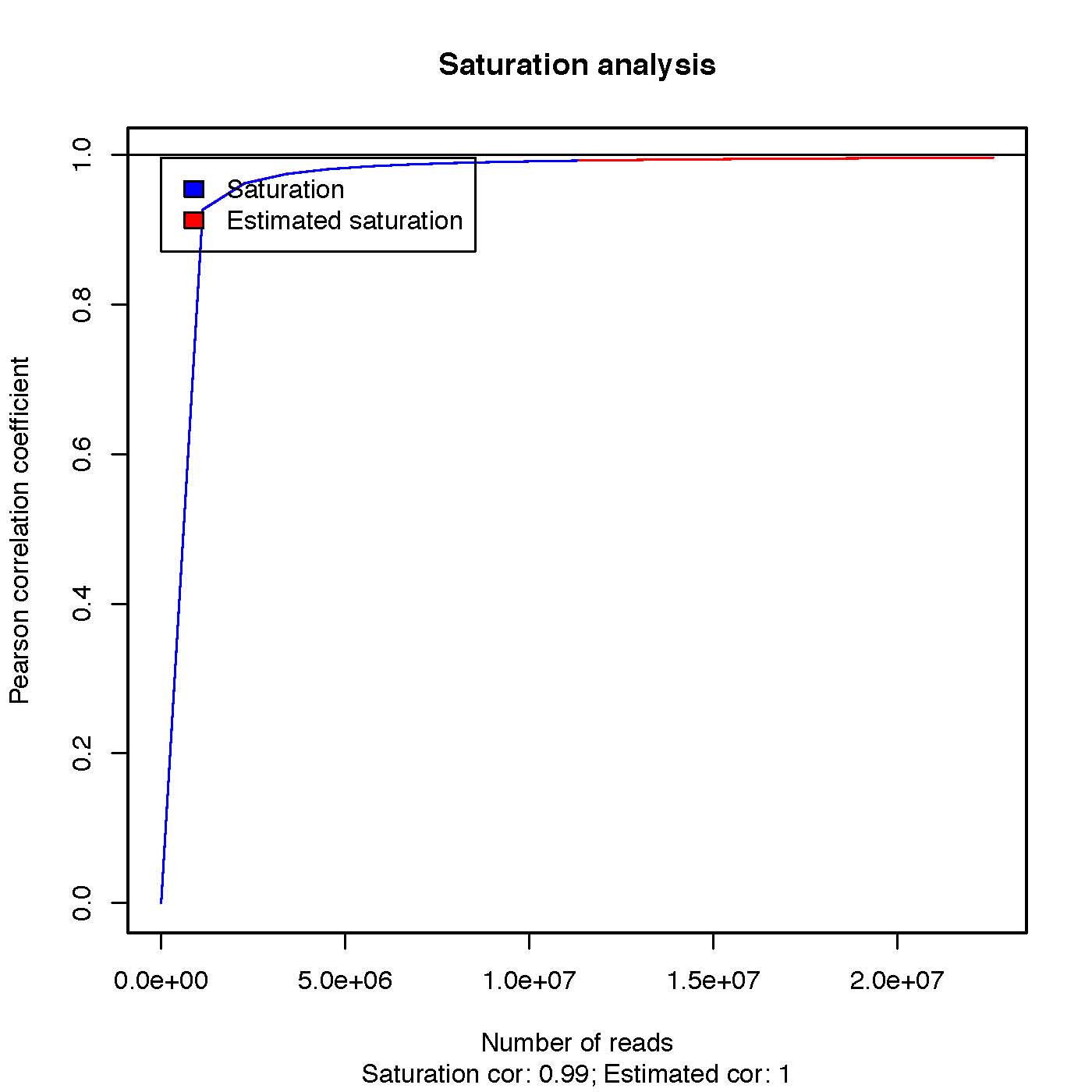

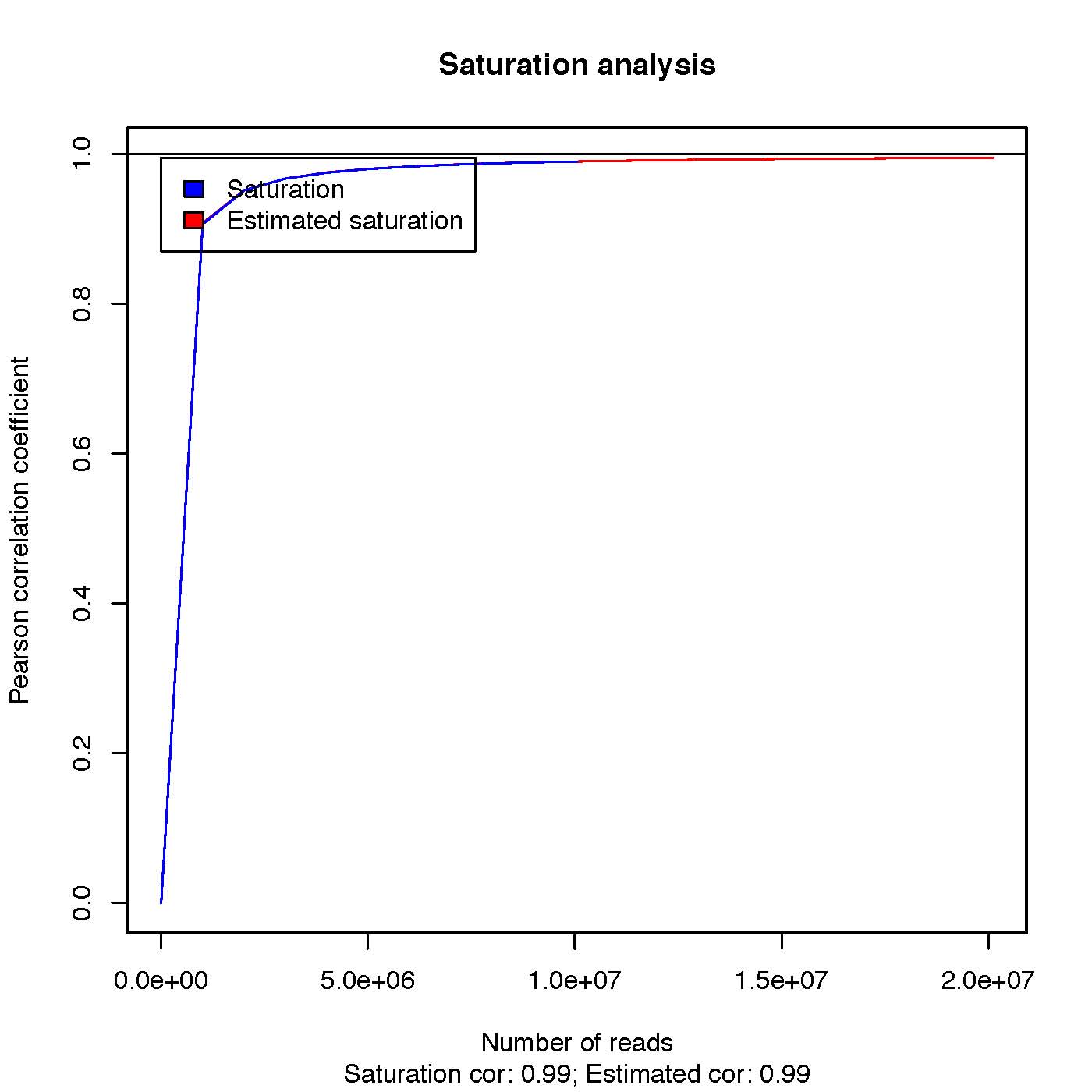


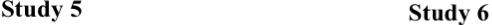


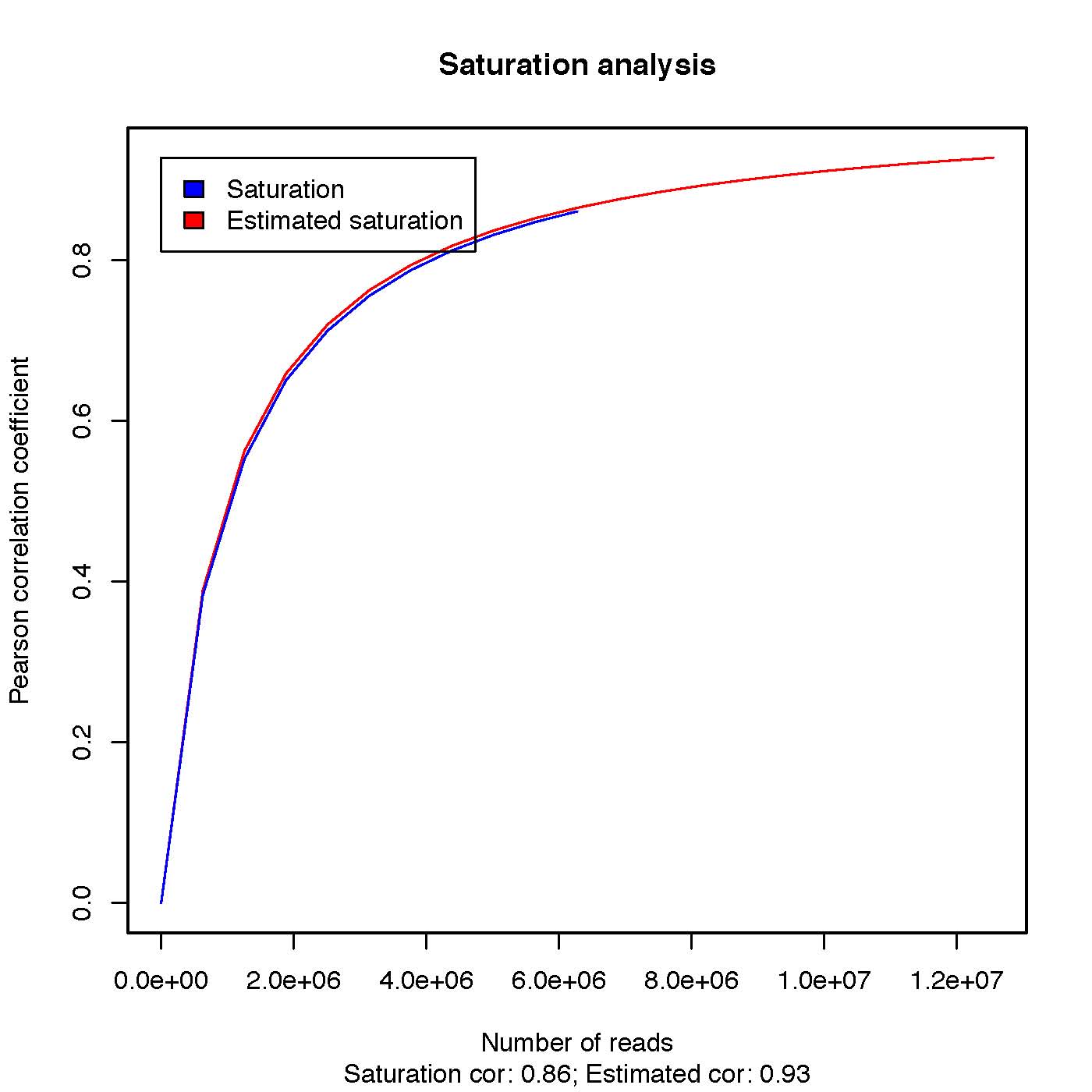

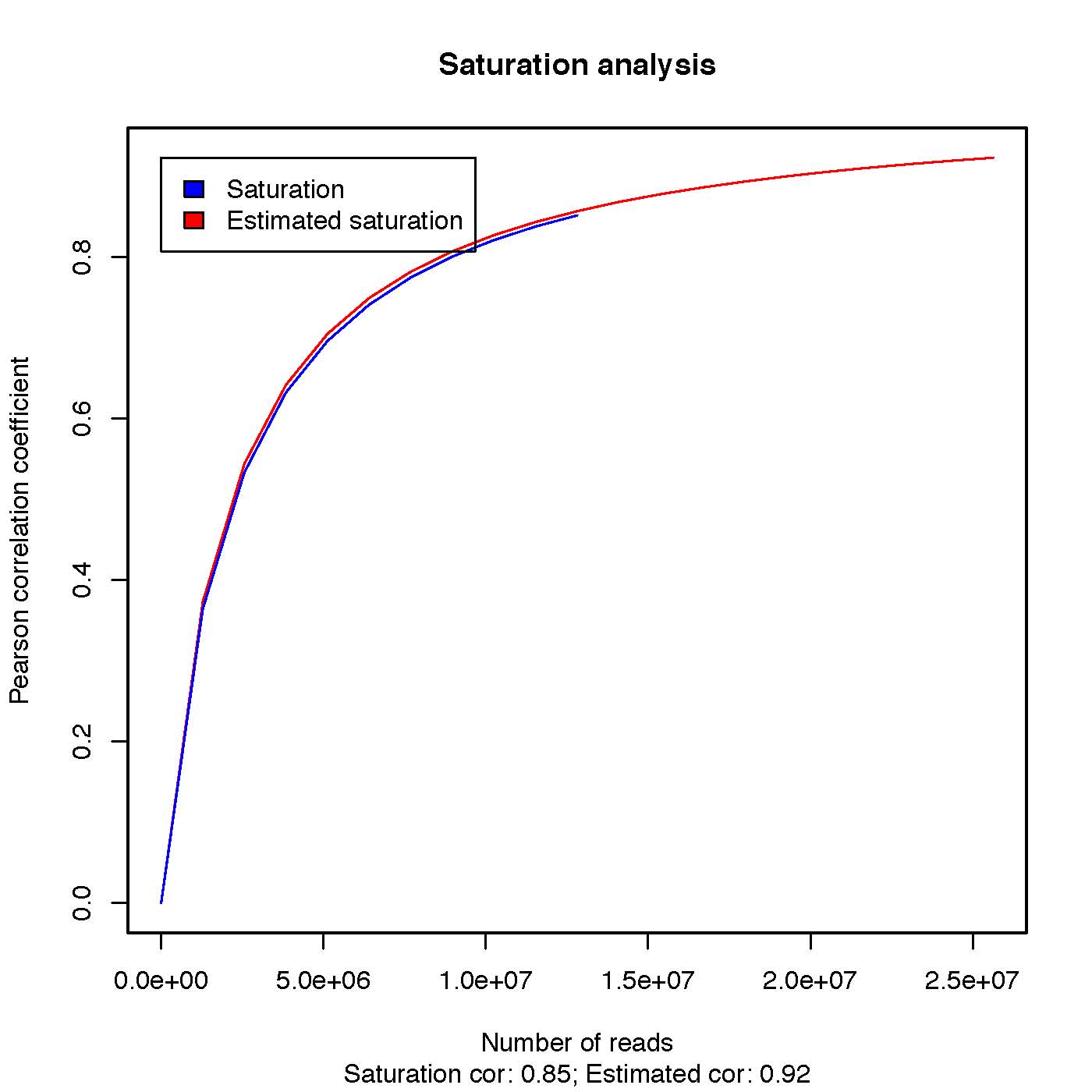


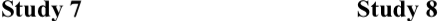


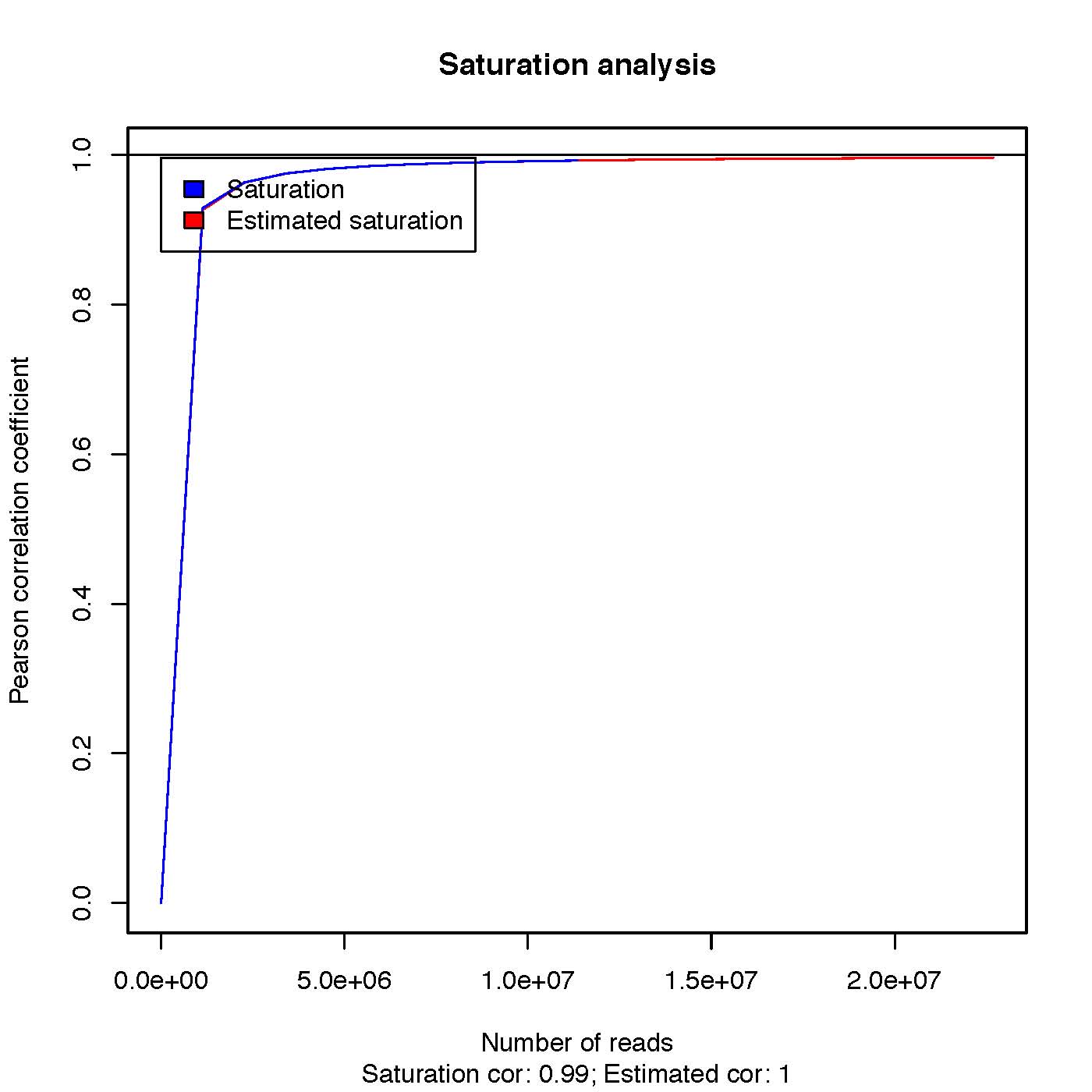

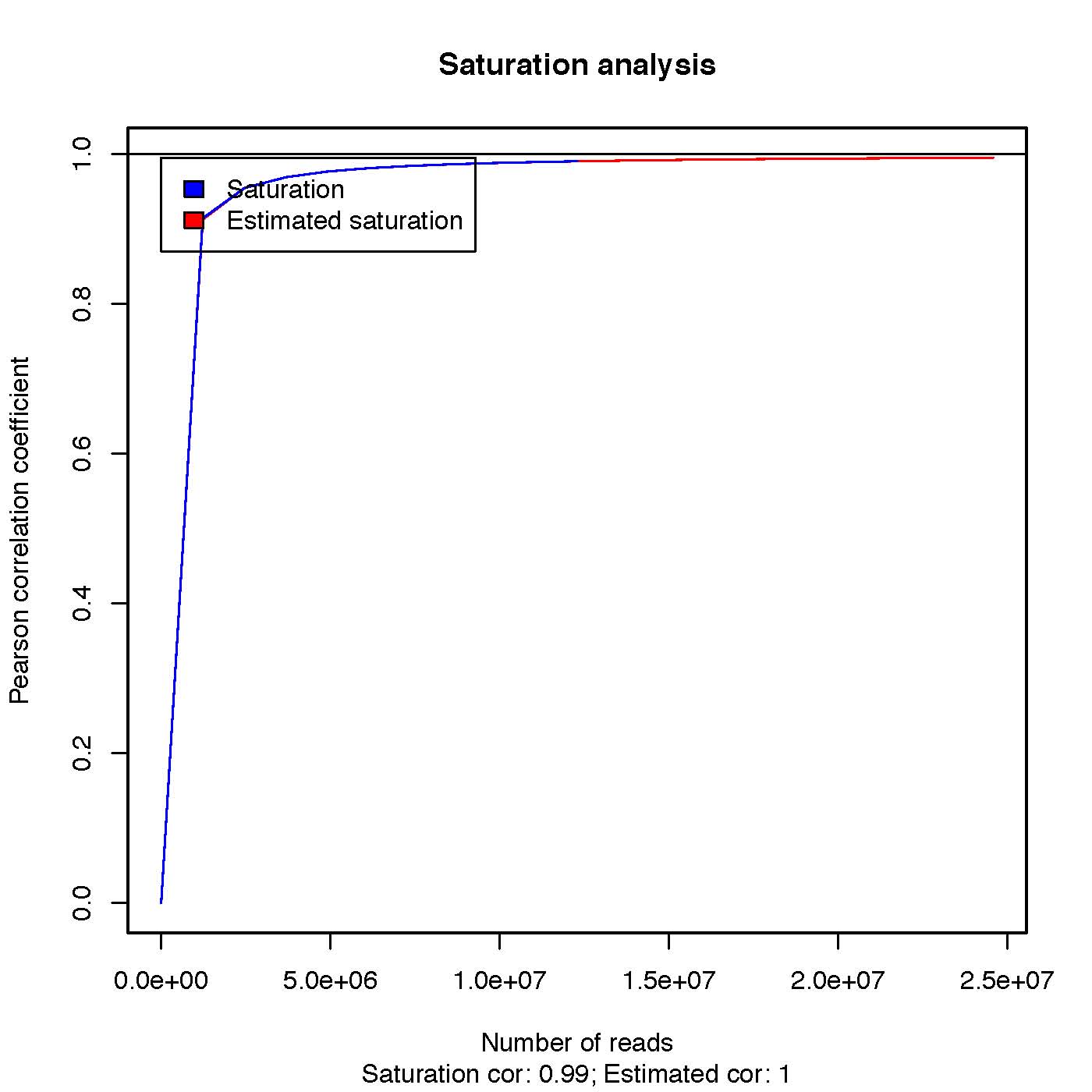


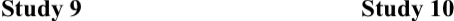


**c.**


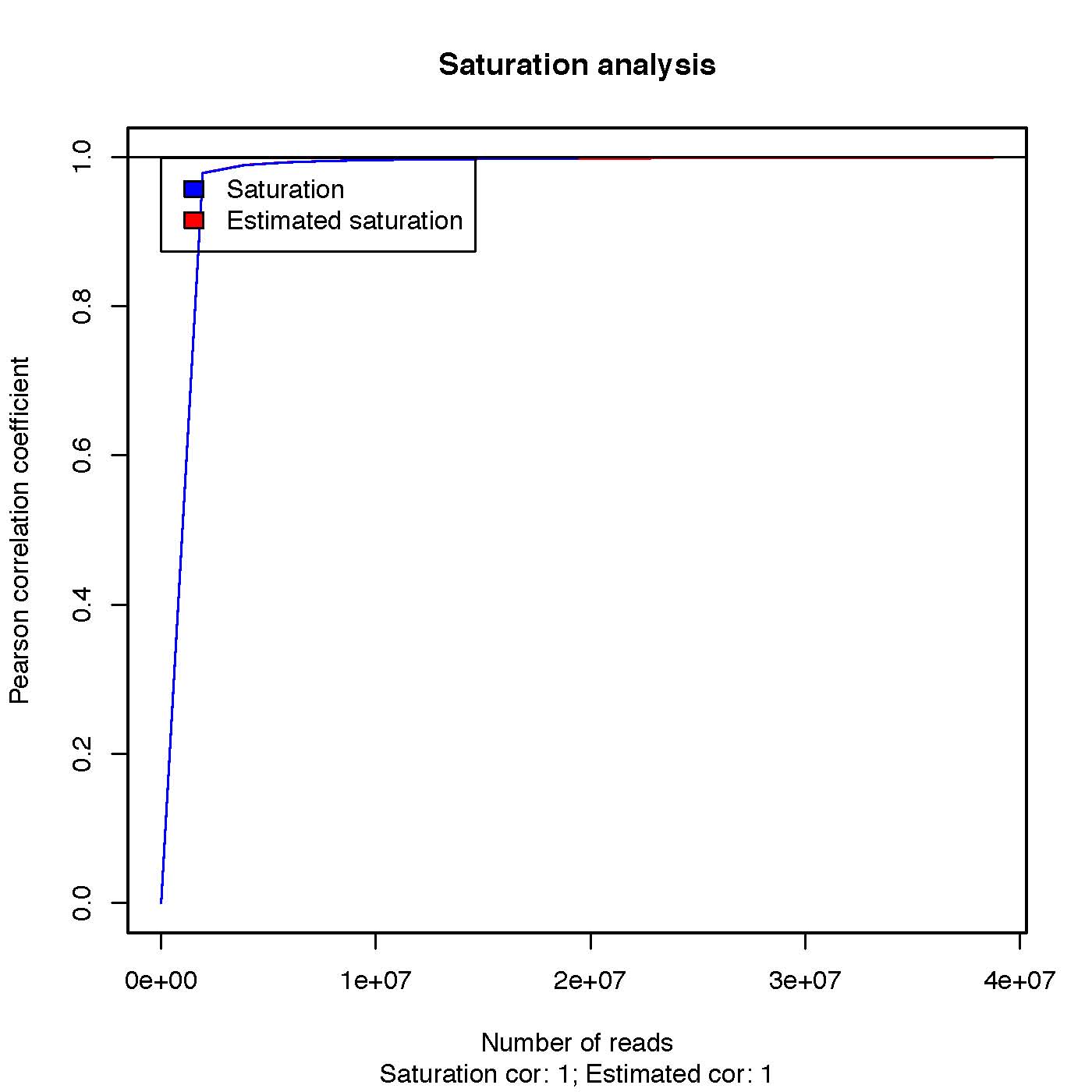

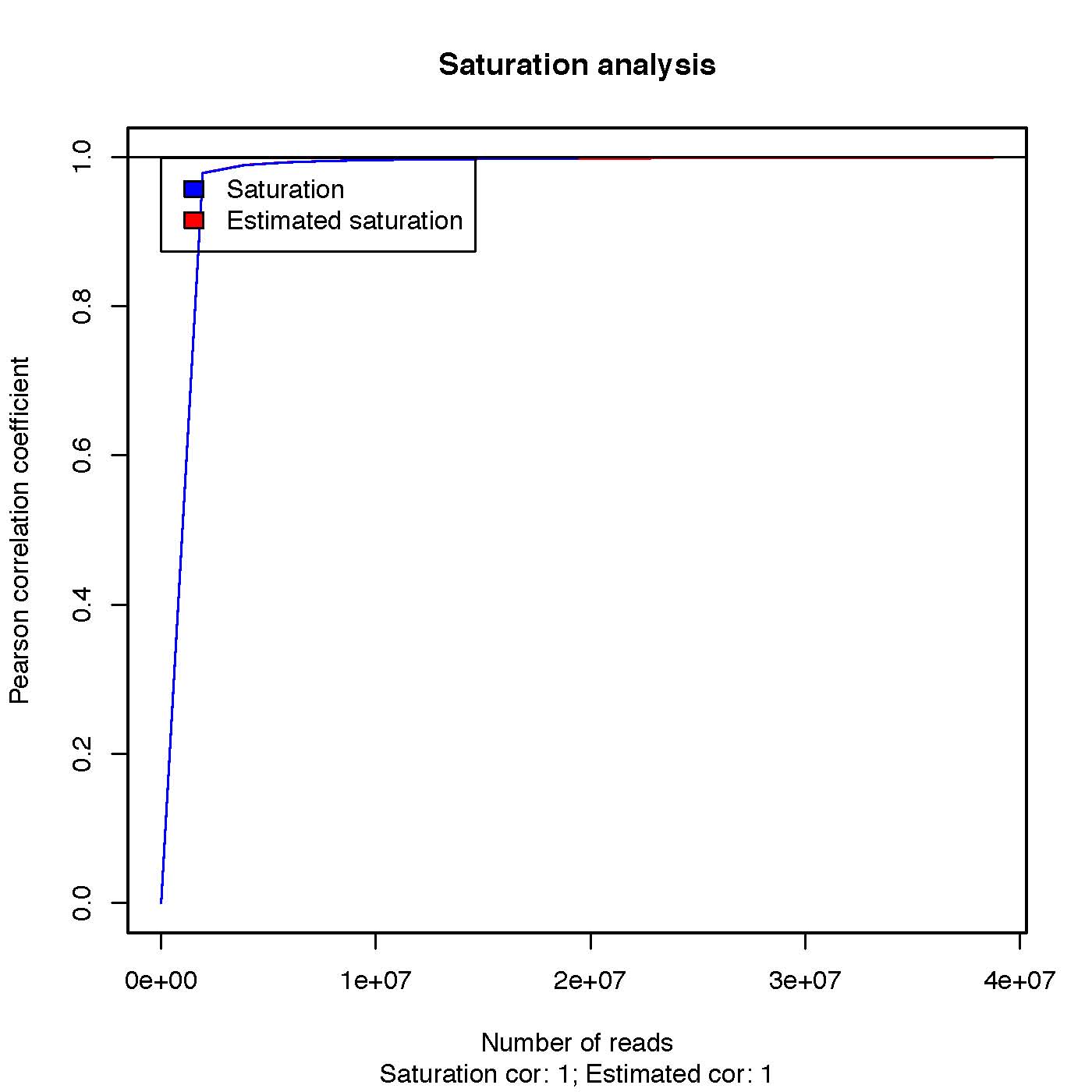


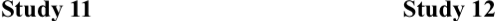


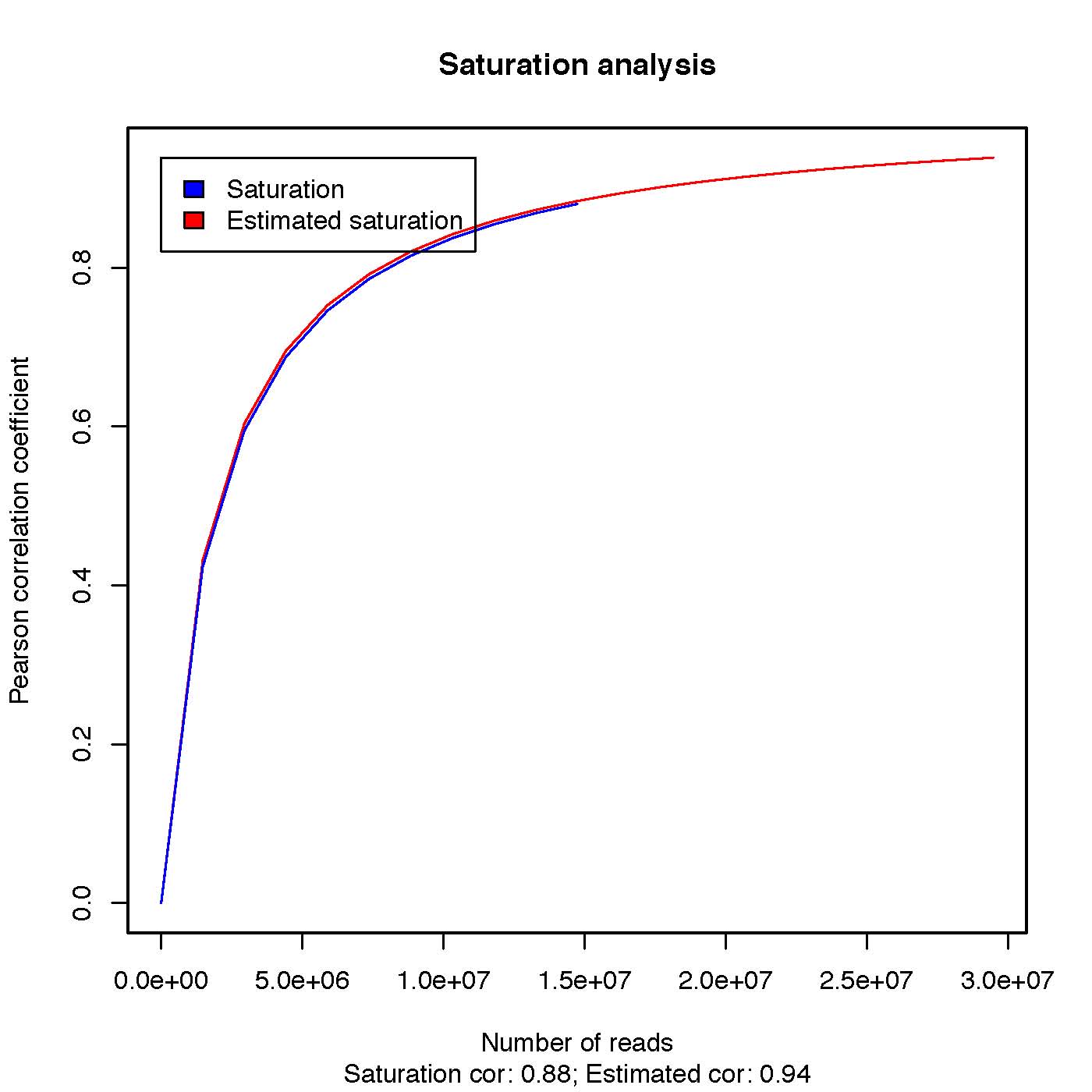

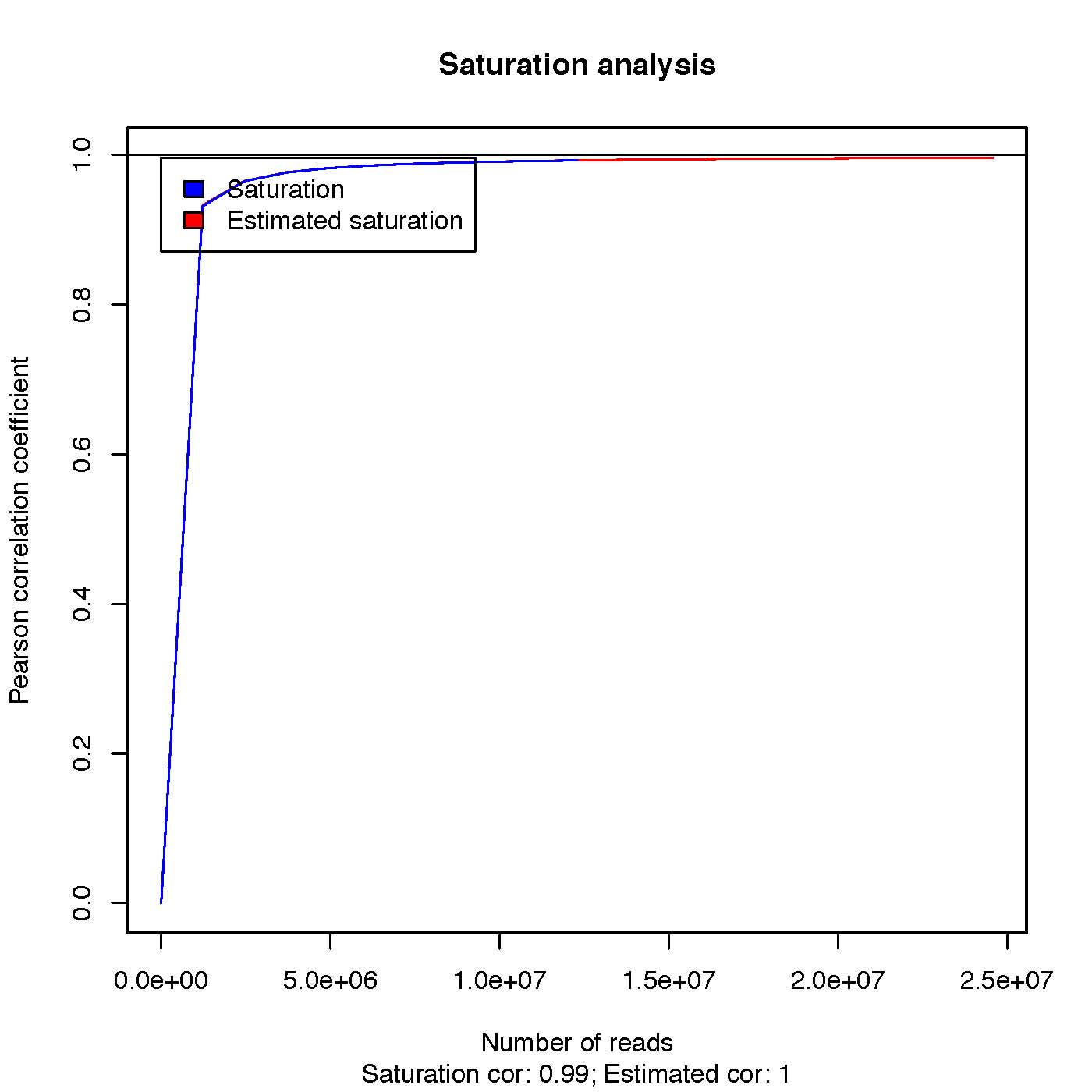


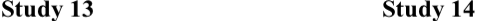


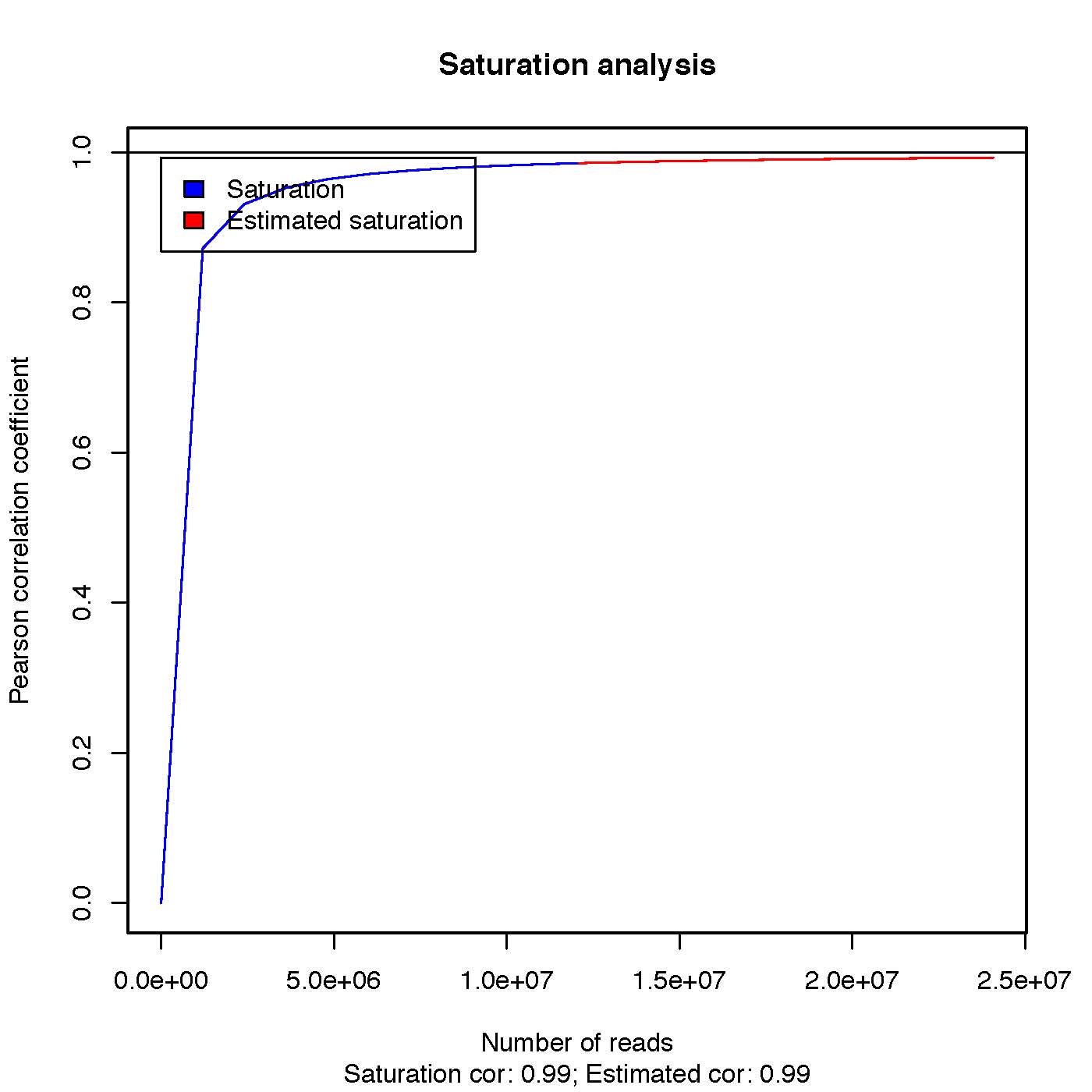

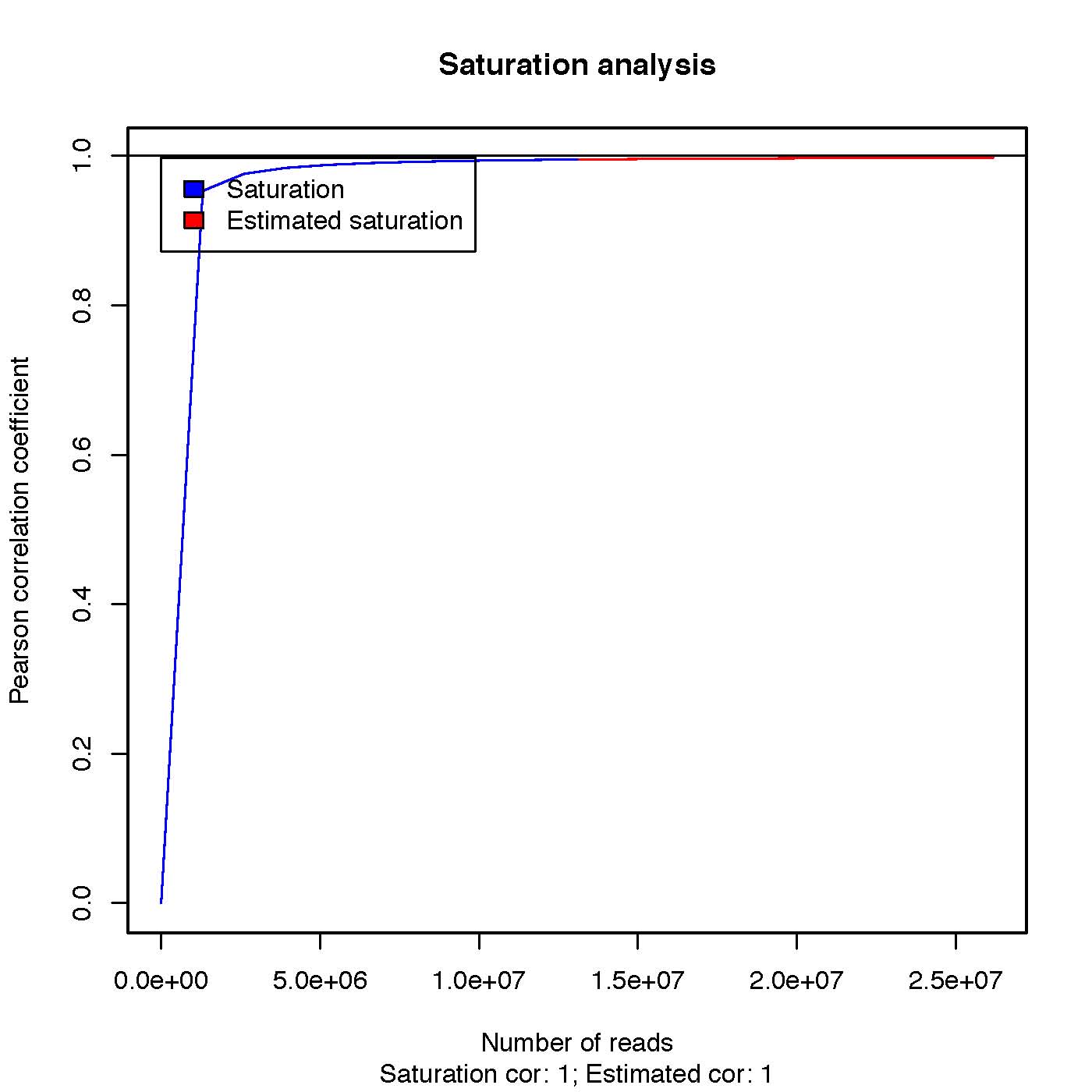


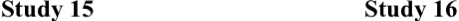

Supplement: Figure S1 — Saturation analysis of MeDIP-seq samples (a, b, c) The saturation analysis investigates whether the number of unique reads is sufficient to generate a saturated and reproducible methylation profile of the reference genome. The higher Pearson correlation r the greater assurance of the reproducibility of the methylation profiles. Sample study number is identified in Table S1. (DOC) [file pone.0033213.s002.doc]
