# Supplementary material for: Genome Wide Analysis of Acute Myeloid Leukemia Reveal Leukemia Specific Methylome and Subtype Specific Hypomethylation of Repeats
Source: PLoS One. 2012 Mar 29;7(3):e33213. doi: 10.1371/journal.pone.0033213 (PMC3315563; doi:10.1371/journal.pone.0033213)
Supplement: Figure S2 — Correlation between MeDIP-seq and Illumina Infinium array. A significant positive correlation was found between MeDIP-seq and Illumina Infinium array in the three MeDIP-seq samples. (DOC) [file pone.0033213.s003.doc]

**Figure S2. Correlation between MeDIP-seq and Illumina Infinium array.** A significant positive correlation was found between MeDIP-seq and Illumina Infinium array in the three MeDIP-seq samples

**
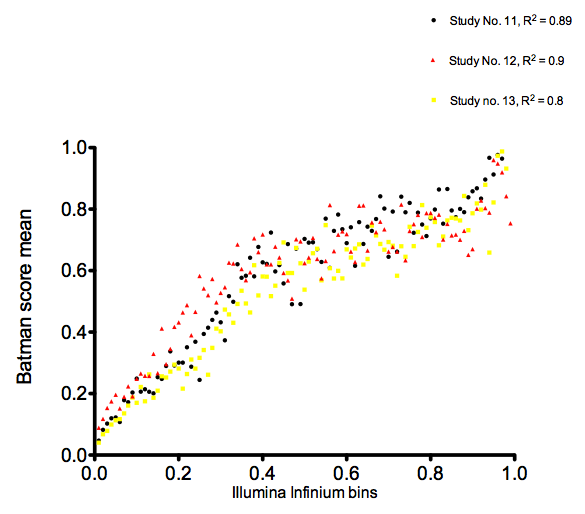
**
