# Supplementary material for: Genome Wide Analysis of Acute Myeloid Leukemia Reveal Leukemia Specific Methylome and Subtype Specific Hypomethylation of Repeats
Source: PLoS One. 2012 Mar 29;7(3):e33213. doi: 10.1371/journal.pone.0033213 (PMC3315563; doi:10.1371/journal.pone.0033213)
Supplement: Figure S3 — Pair-wise comparison between AML and NBMs in 4 genomic regions. Red colored spots indicate high similarity and white colored spots low similarity. CGIs (C) showed the highest similarities between AML subtypes and a clear discrimination from NBMs. (A) Promoters, (B) Gene bodies, (D) CGI shores. (DOC) [file pone.0033213.s004.doc]

**Figure S3 Pair-wise comparison between AML and NBMs in 4 genomic regions.** Red colored spots indicate high similarity and white colored spots low similarity. CGIs (C) showed the highest similarities between AML subtypes and a clear discrimination from NBMs. (A) Promoters, (B) Gene bodies, (D) CGI shores.


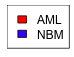


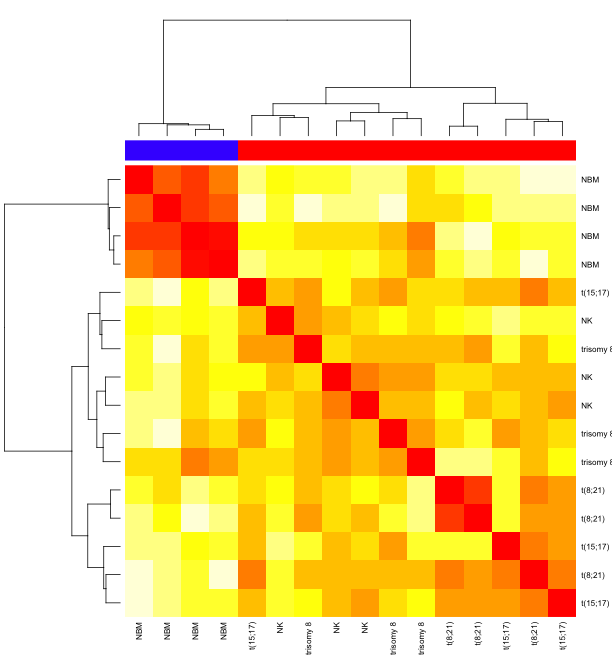

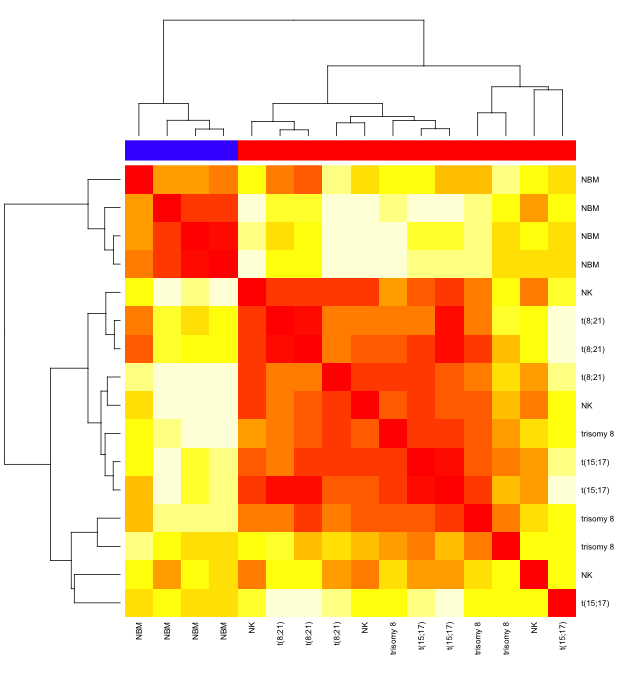


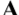

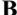


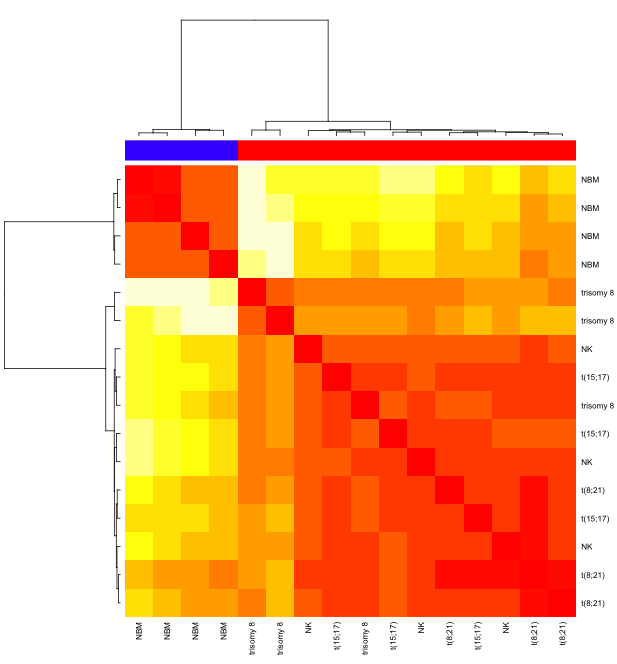

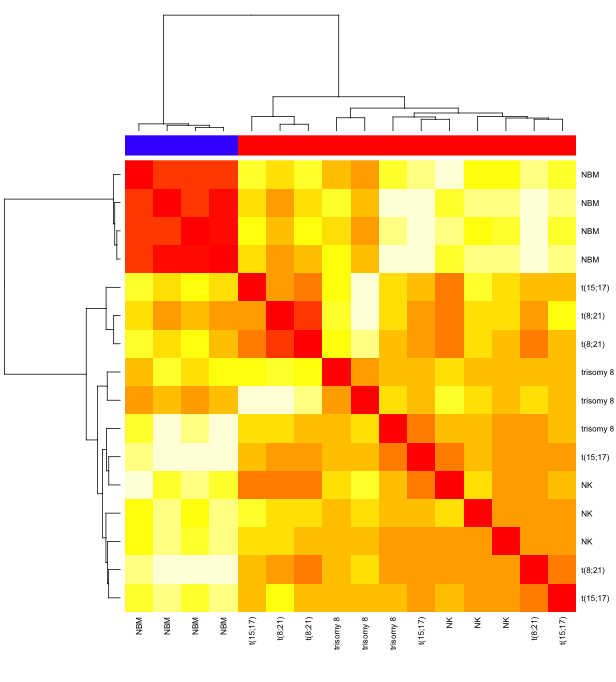


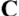

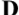


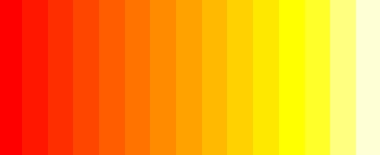


high low

Similarity
