# Supplementary material for: Genome Wide Analysis of Acute Myeloid Leukemia Reveal Leukemia Specific Methylome and Subtype Specific Hypomethylation of Repeats
Source: PLoS One. 2012 Mar 29;7(3):e33213. doi: 10.1371/journal.pone.0033213 (PMC3315563; doi:10.1371/journal.pone.0033213)

**Figure S4. Estimating the number of clusters in data set consisting of differentially methylated CGIs using prediction strength method**

The consecutive number of clusters is given on the x-axis. The vertical bars illustrate the standard error of the prediction strength over 5 cross-validation folds. Prediction strength above 0.8 indicates well-separated clusters. Dividing the CGI associated DMRs data set into two clusters, referring to AML and NBM groups, gives the highest stability.


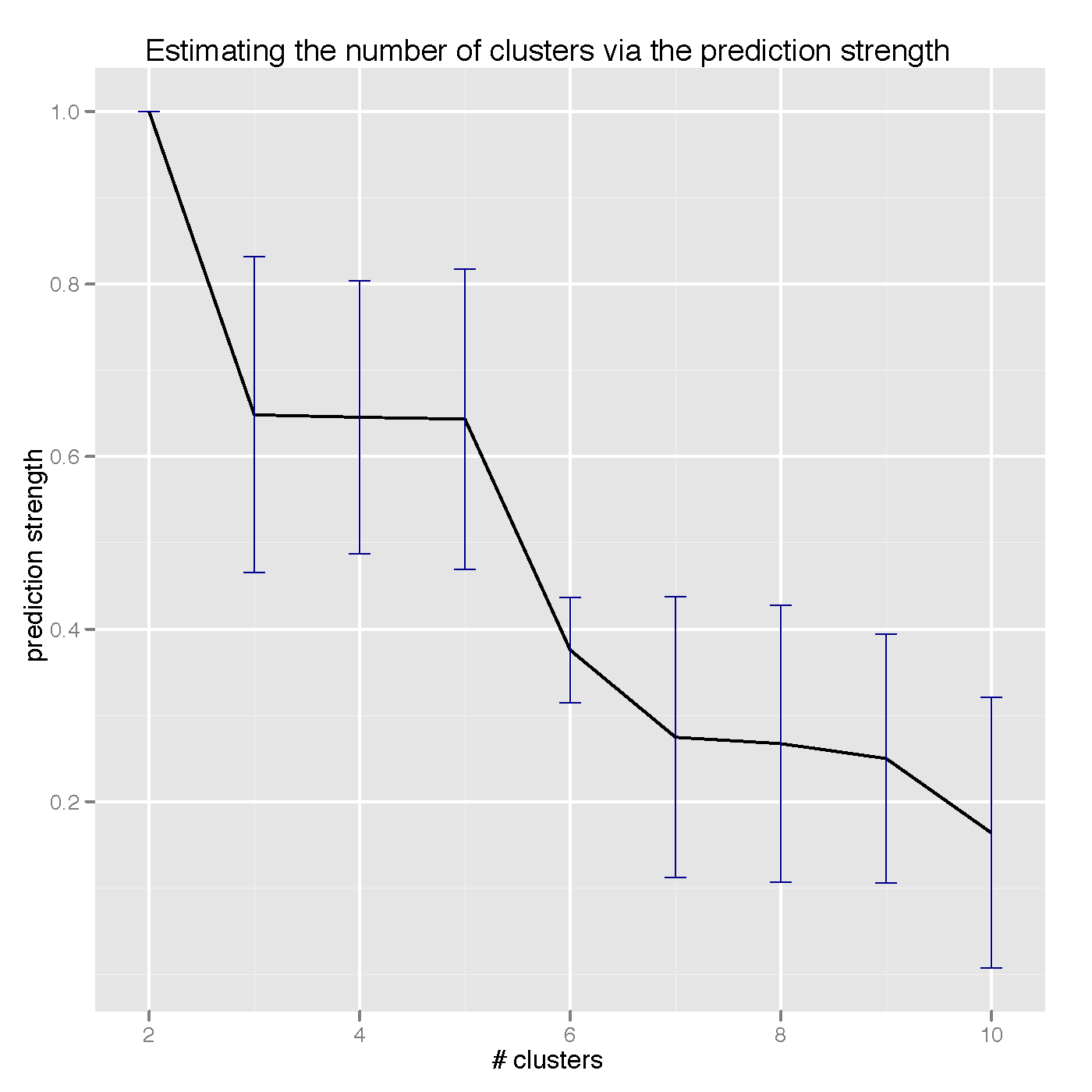

Supplement: Figure S4 — Estimating the number of clusters in data set consisting of differentially methylated CGIs using prediction strength method The consecutive number of clusters is given on the x-axis. The vertical bars illustrate the standard error of the prediction strength over 5 cross-validation folds. Prediction strength above 0.8 indicates well-separated clusters. Dividing the CGI associated DMRs data set into two clusters, referring to AML and NBM groups, gives the highest stability. (DOC) [file pone.0033213.s005.doc]
