# Supplementary material for: Genome Wide Analysis of Acute Myeloid Leukemia Reveal Leukemia Specific Methylome and Subtype Specific Hypomethylation of Repeats
Source: PLoS One. 2012 Mar 29;7(3):e33213. doi: 10.1371/journal.pone.0033213 (PMC3315563; doi:10.1371/journal.pone.0033213)
Supplement: Figure S5 — Characters of DMRs in AML subtypes. Venn diagrams showed few overlapped DMRs between AML subtypes with no common DMRs detected between the all 4 AML subtypes. (DOC) [file pone.0033213.s006.doc]

**Figure S5. Characters of DMRs in AML subtypes.**

Venn diagrams showed few overlapped DMRs between AML subtypes with no common DMRs detected between the all 4 AML subtypes.

|  | Hypermethylated DMRs | Hypomethylated DMRs |
| --- | --- | --- |
| Promoters | 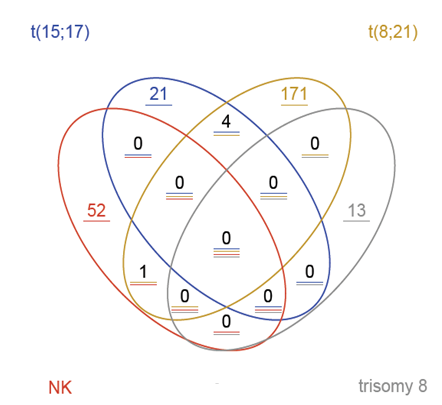 | 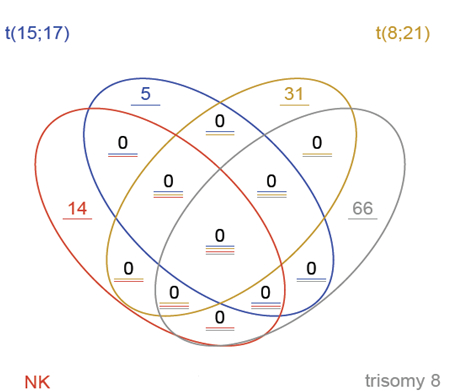 |
| Gene bodies | 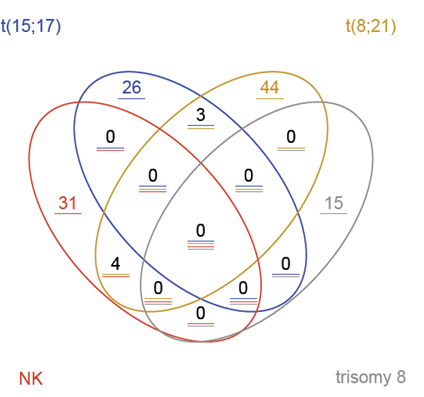 | 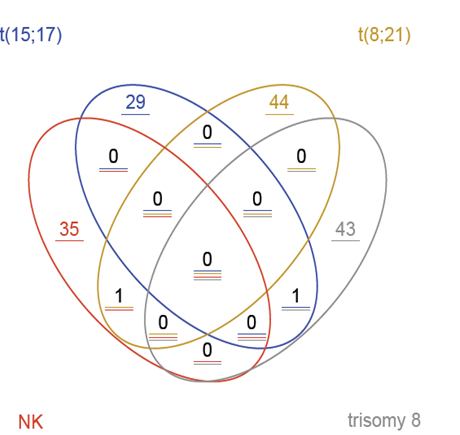 |
| CGIs | 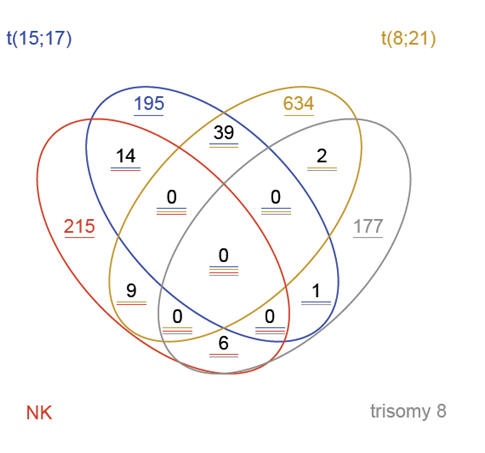 | 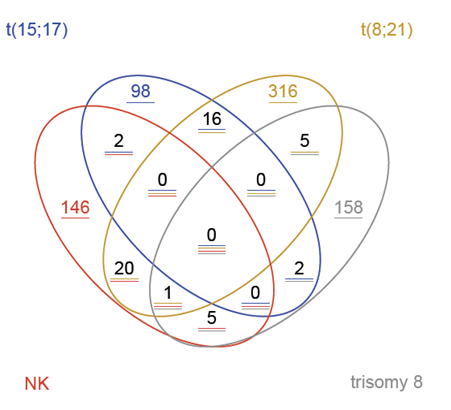 |
| CGI shores | 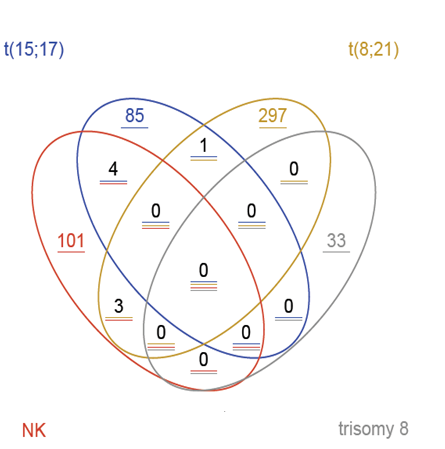 | 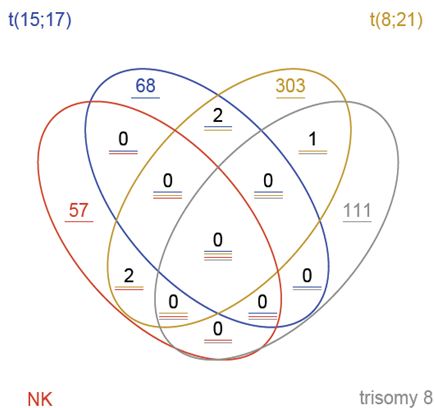 |
