# Supplementary material for: Genome Wide Analysis of Acute Myeloid Leukemia Reveal Leukemia Specific Methylome and Subtype Specific Hypomethylation of Repeats
Source: PLoS One. 2012 Mar 29;7(3):e33213. doi: 10.1371/journal.pone.0033213 (PMC3315563; doi:10.1371/journal.pone.0033213)
Supplement: Figure S6 — Pair-wise comparison between AML subtypes in 4 genomic regions. t(8;21) AML subtype is discriminated distantly from all the other AML subtypes. (A) Promoter, (B) gene bodies, (C) CGIs, (D) CGI shores. (DOC) [file pone.0033213.s007.doc]

**Figure S6. Pair-wise comparison between AML subtypes in 4 genomic regions.**

t(8;21) AML subtype is discriminated distantly from all the other AML subtypes. (A) Promoter, (B) gene bodies, (C) CGIs, (D) CGI shores.


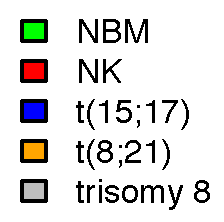


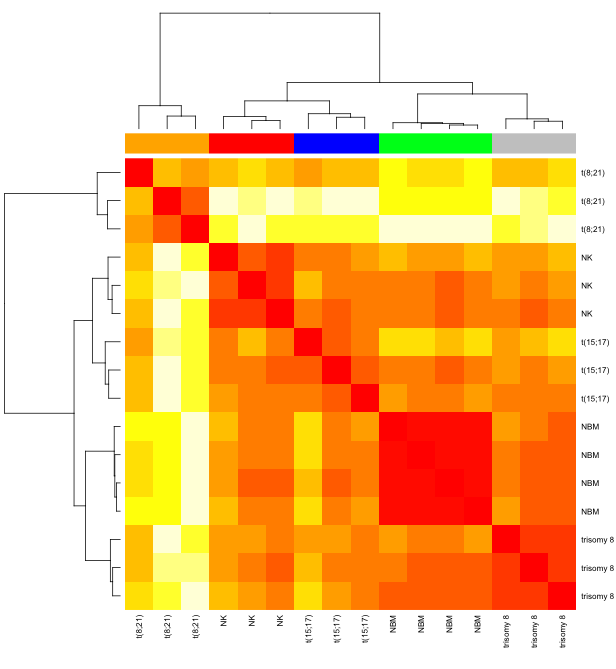

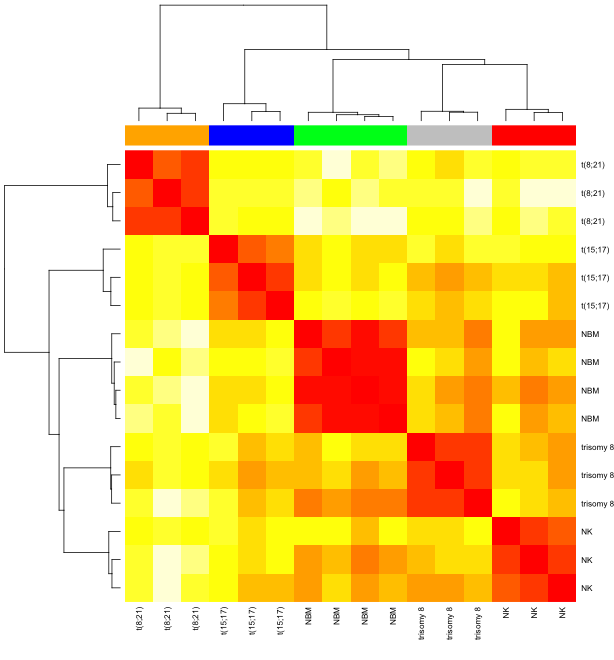


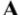

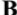


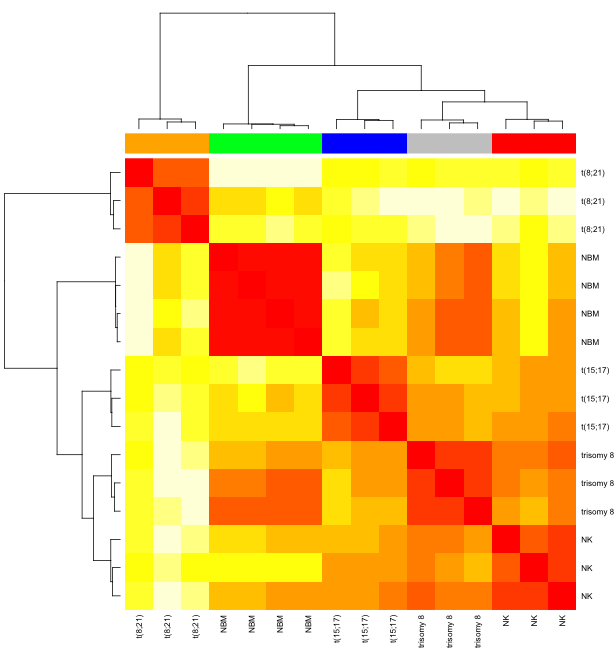

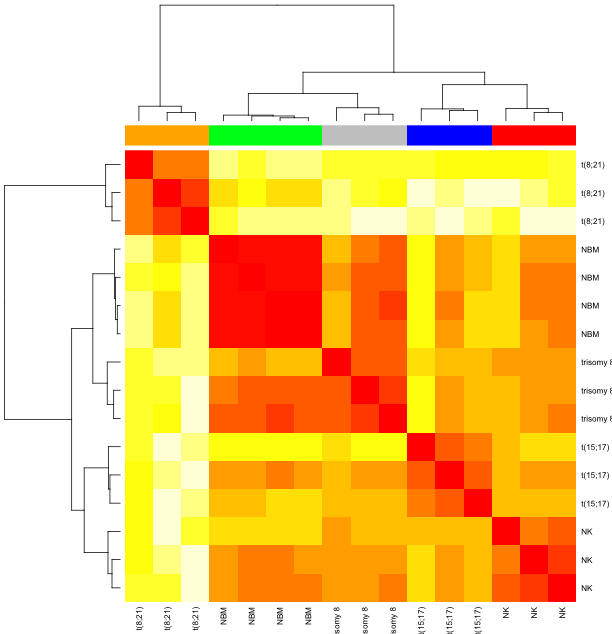


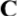

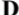


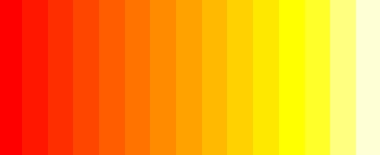


high low

Similarity
