# Supplementary material for: Genome Wide Analysis of Acute Myeloid Leukemia Reveal Leukemia Specific Methylome and Subtype Specific Hypomethylation of Repeats
Source: PLoS One. 2012 Mar 29;7(3):e33213. doi: 10.1371/journal.pone.0033213 (PMC3315563; doi:10.1371/journal.pone.0033213)
Supplement: Figure S7 — Pair-wise comparison between AML and NBMs in repeat sequences. (A) SINEs showed the highest similarities between AML subtypes among other repeats; (B) LINEs and (C) LTRs. (DOC) [file pone.0033213.s008.doc]

**Figure S7 Pair-wise comparison between AML and NBMs in repeat sequences.** (A) SINEs showed the highest similarities between AML subtypes among other repeats; (B) LINEs and (C) LTRs.


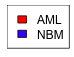


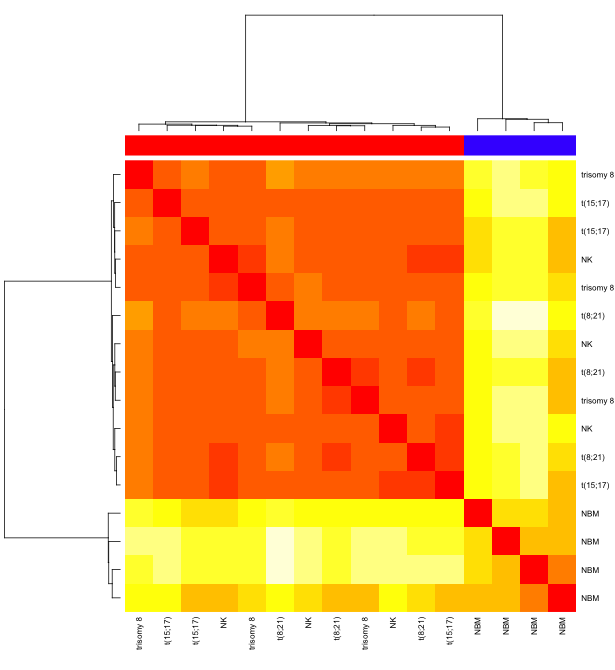

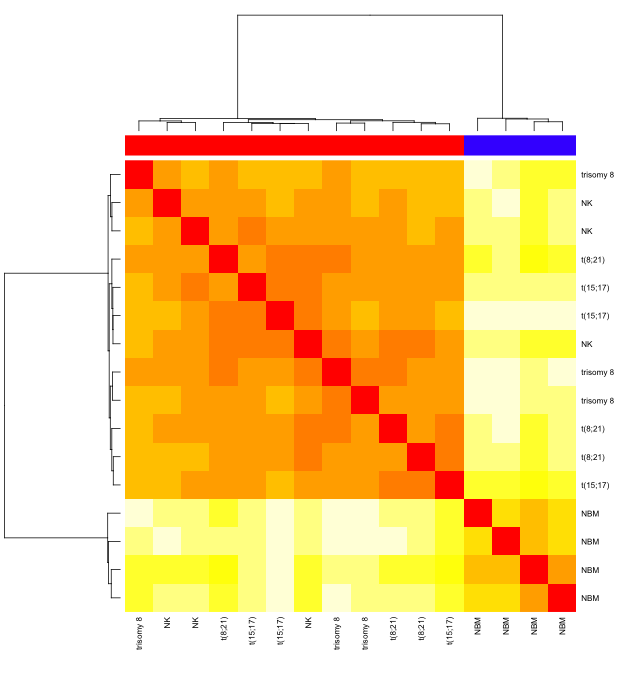


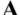

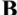


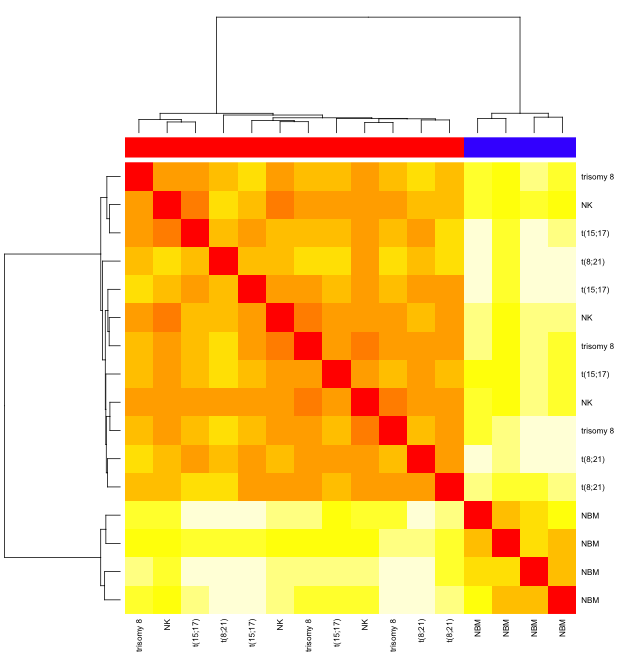


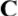


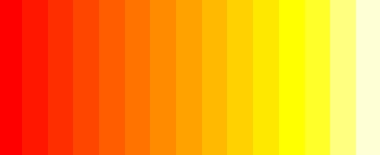


high low
