# Supplementary material for: Genome Wide Analysis of Acute Myeloid Leukemia Reveal Leukemia Specific Methylome and Subtype Specific Hypomethylation of Repeats
Source: PLoS One. 2012 Mar 29;7(3):e33213. doi: 10.1371/journal.pone.0033213 (PMC3315563; doi:10.1371/journal.pone.0033213)
Supplement: Figure S8 — Pair-wise comparison between AML subtypes in repeat sequences. There was clear discrimination between AML subtypes in (A) SINEs, (B) LINEs and (C) LTRs. (DOC) [file pone.0033213.s009.doc]

**Figure S8. Pair-wise comparison between AML subtypes in repeat sequences.** There was clear discrimination between AML subtypes in (A) SINEs, (B) LINEs and (C) LTRs.


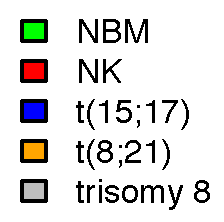


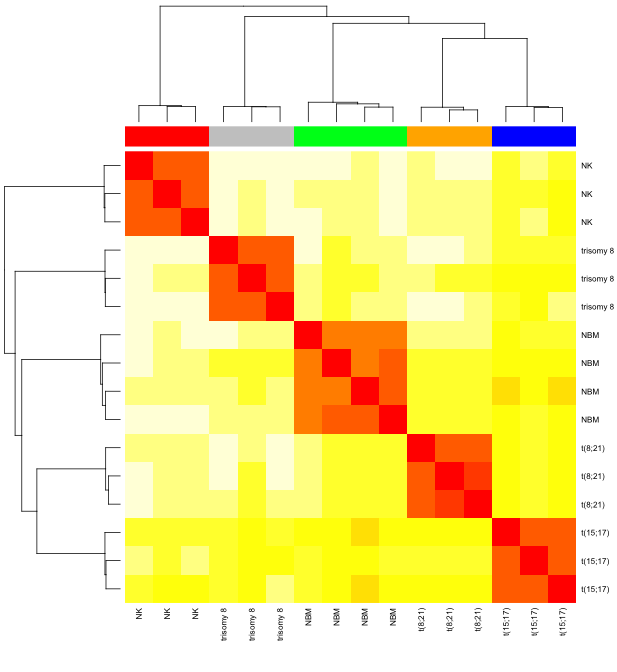

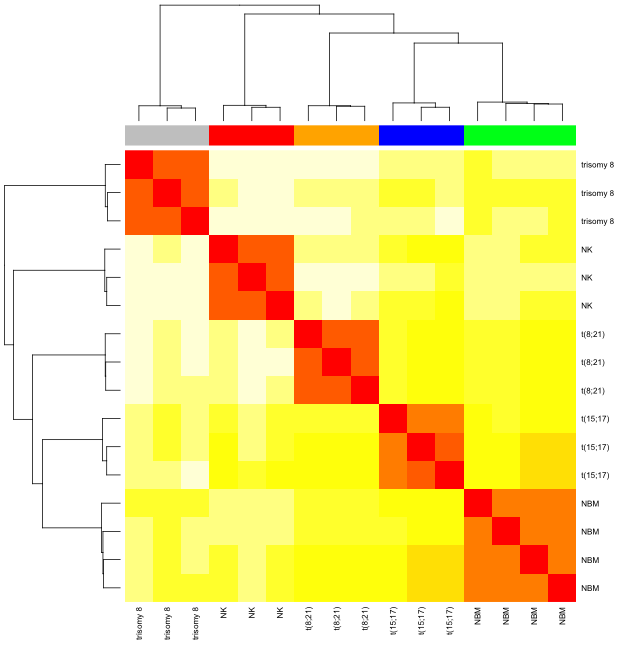


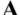

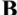


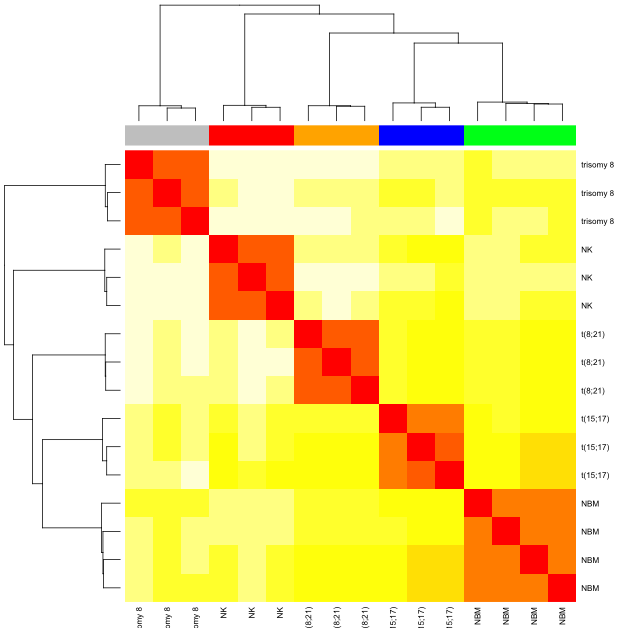


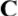


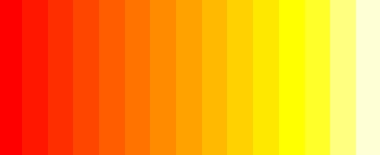


high low

Similarity
