# Supplementary material for: Genome Wide Analysis of Acute Myeloid Leukemia Reveal Leukemia Specific Methylome and Subtype Specific Hypomethylation of Repeats
Source: PLoS One. 2012 Mar 29;7(3):e33213. doi: 10.1371/journal.pone.0033213 (PMC3315563; doi:10.1371/journal.pone.0033213)
Supplement: Figure S9 — Direct bisulfite sequencing of significant differentially methylated genes/genomic regions in MeDIP-seq samples. (a, b, c, d, e) For all figures, the horizontal line represents the position of each CpG investigated and the vertical line is the percentage of the methylation at particular CpG site from 0–100%. The analysis was performed using QUMA. (DOC) [file pone.0033213.s010.doc]

**Direct bisulfite sequencing of significant differentially methylated genes/genomic regions in MeDIP-seq samples.** (a, b, c, d, e) For all figures, the horizontal line represents the position of each CpG investigated and the vertical line is the percentage of the methylation at particular CpG site from 0-100%. The analysis was performed using QUMA.

a. *DPP6* Chr 7: 153214701-153215064

| t(8;21)-1 | 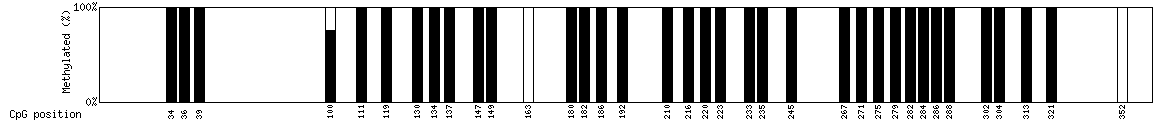 |
| --- | --- |
| t(15;17)-1 | 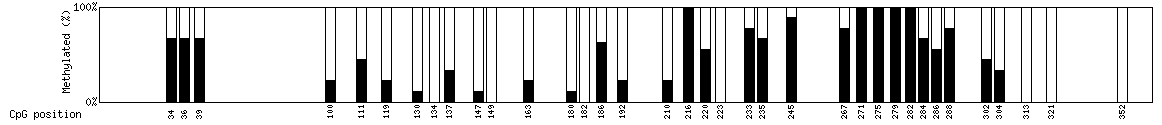 |
| t(8;21)-3 | 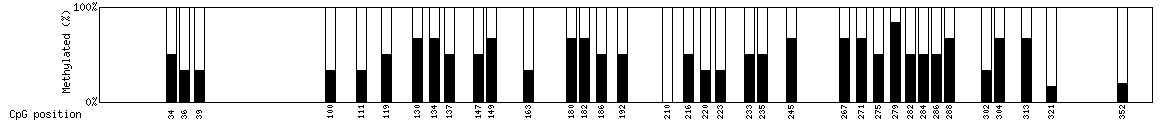 |
| t(15;17)-3 | 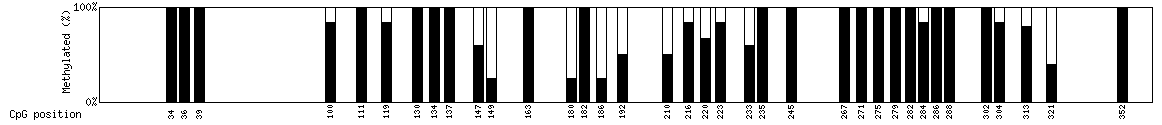 |
| NK-1 | 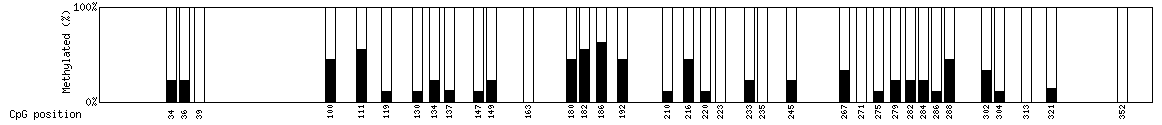 |
| NK-3 | 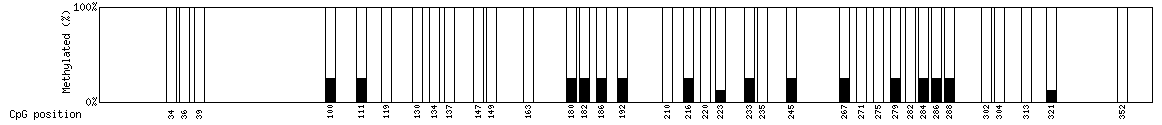 |
| NBM-1 | 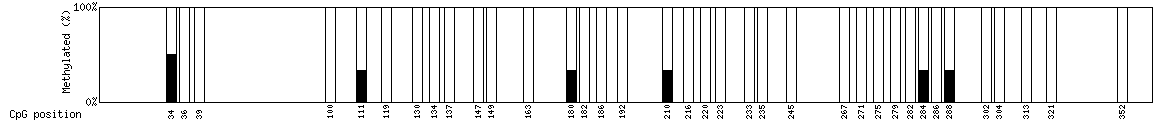 |
| NBM-2 | 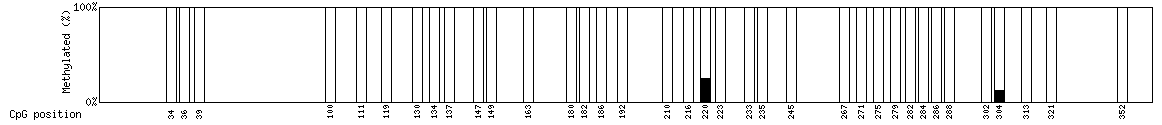 |
| NBM-3 | 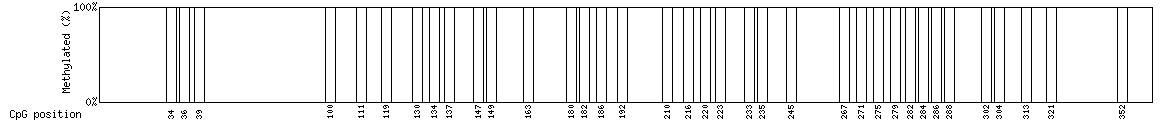 |

b. CGIs Chr20: 60319301-60319515

| t(8; 21)-1 | 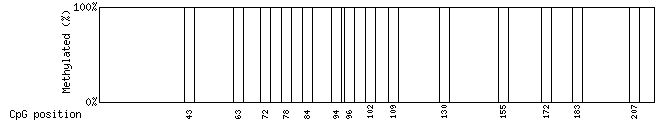 |
| --- | --- |
| t(15; 17)-3 | 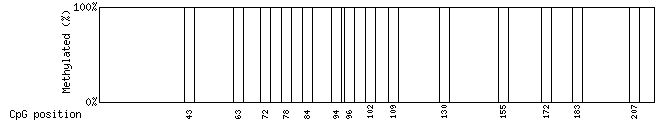 |
| t(8;21)-3 | 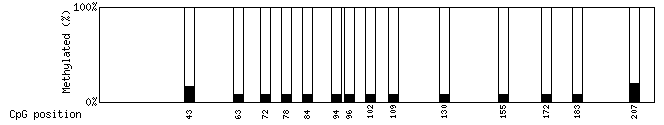 |
| t(15; 17)-2 | 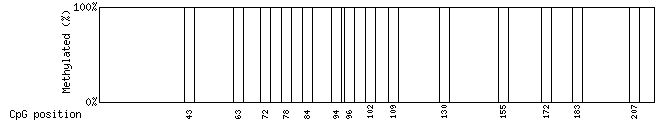 |
| NK-1 | 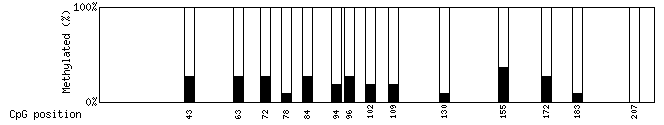 |
| NBM-1 | 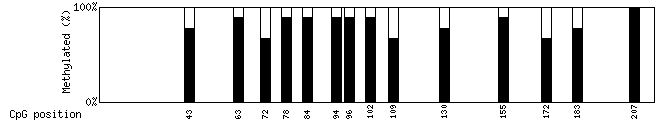 |
| NBM-2 | 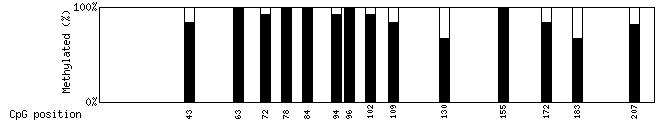 |
| NBM-3 | 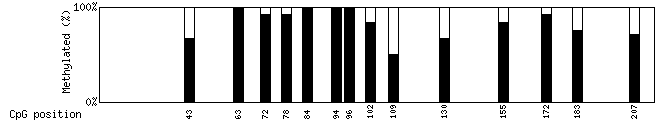 |

c. CpG shore (sequence a) Chr7: 8448450-8448728

| t(8; 21)-1 | 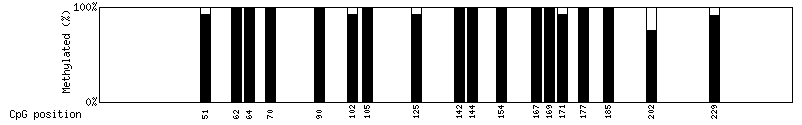 |
| --- | --- |
| t(15; 17)-3 | 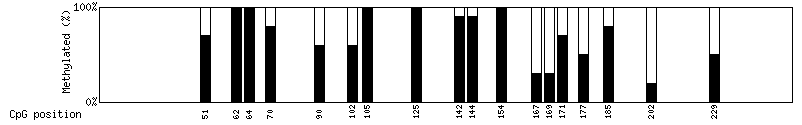 |
| t(8;21)-3 | 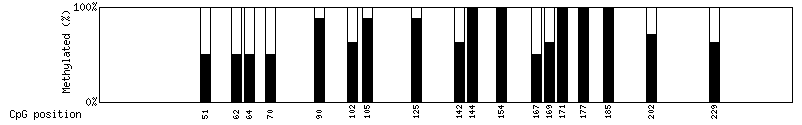 |
| t(15; 17)-2 | 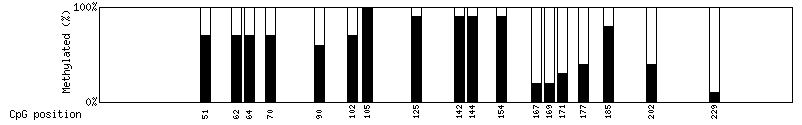 |
| t(15; 17)-1 | 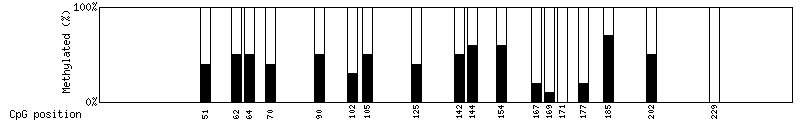 |
| NK-1 | 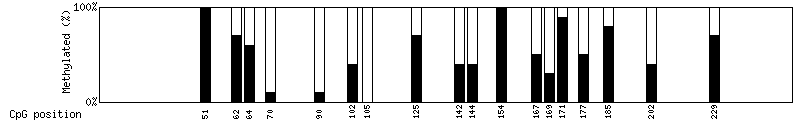 |
| NBM-2 | 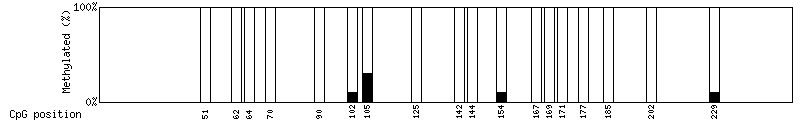 |
| NBM-3 | 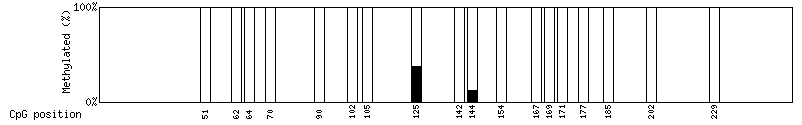 |

d. CpG shore (sequence b) Chr7: 8448727-8448894

| t(8; 21)-1 | 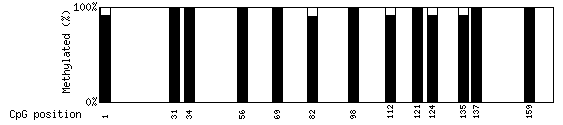 |
| --- | --- |
| t(15; 17)-1 | 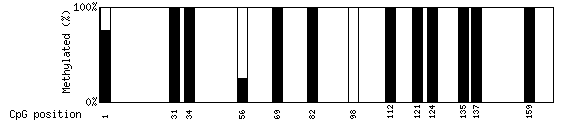 |
| t(15; 17)-3 | 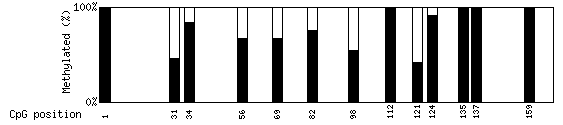 |
| NK-3 | 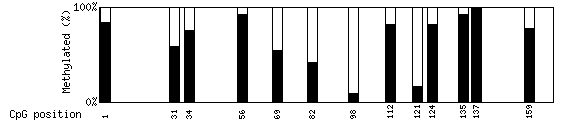 |
| NBM-1 | 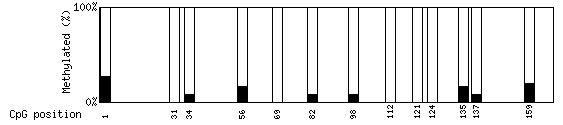 |
| NBM-2 | 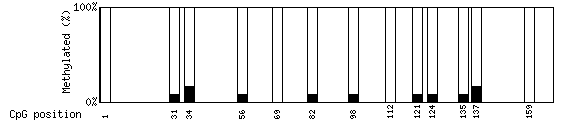 |
| NBM-3 | 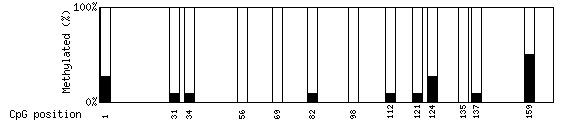 |
| NBM-4 | 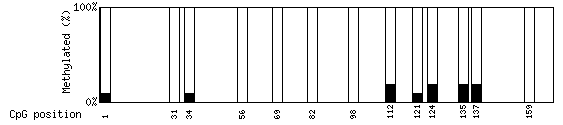 |

*e. SPHKAP* Chr2: 228754201-228754550

| t(8; 21)-1 | 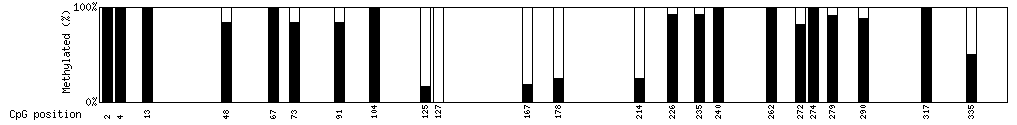 |
| --- | --- |
| t(8;21)-3 | 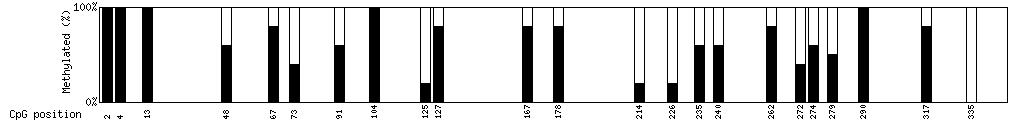 |
| t(15; 17)-1 | 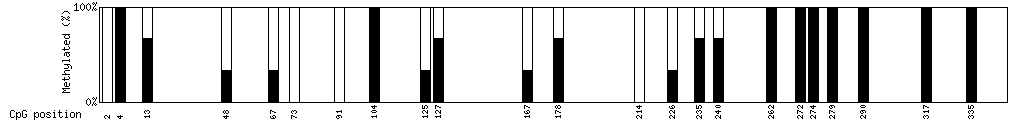 |
| t(15; 17)-2 | 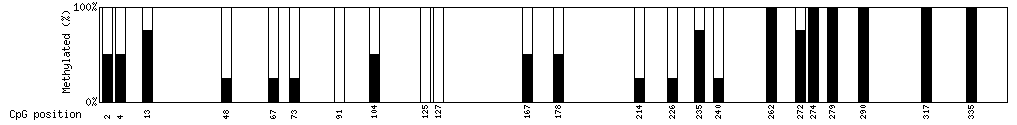 |
| t(15; 17)-3 | 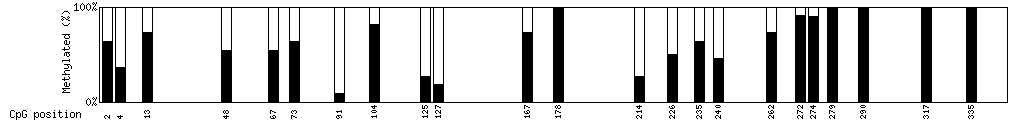 |
| NK-1 | 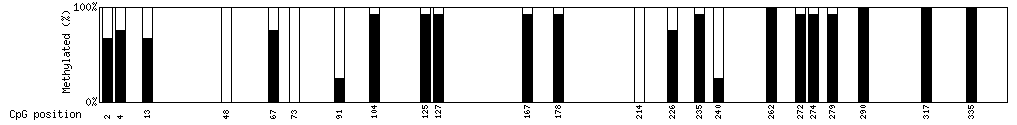 |
| NK-3 | 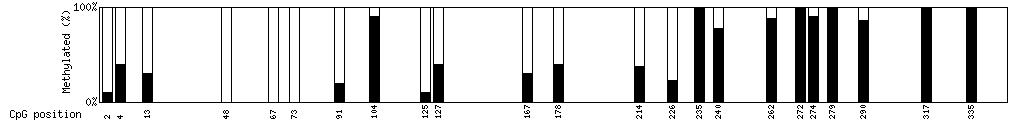 |
| NBM-1 | 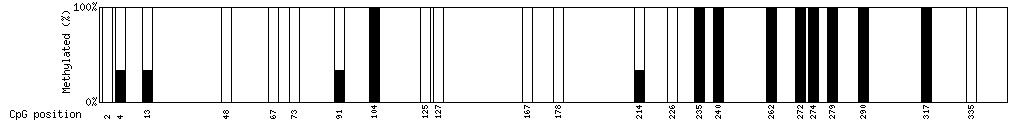 |
| NBM-2 | 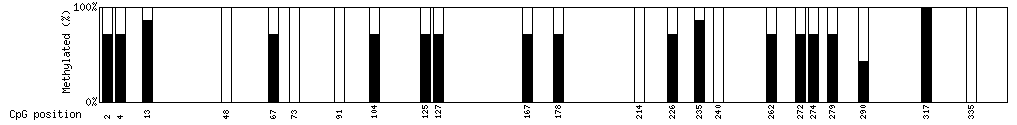 |
| NBM-3 | 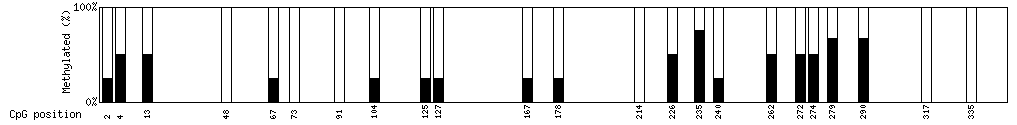 |
| NBM-4 | 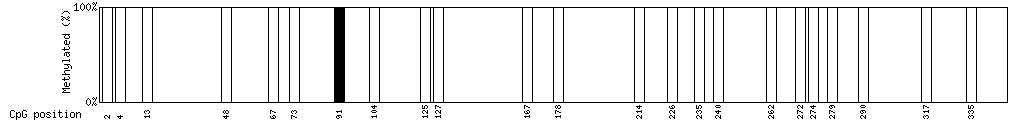 |
