# Supplementary material for: Genome Wide Analysis of Acute Myeloid Leukemia Reveal Leukemia Specific Methylome and Subtype Specific Hypomethylation of Repeats
Source: PLoS One. 2012 Mar 29;7(3):e33213. doi: 10.1371/journal.pone.0033213 (PMC3315563; doi:10.1371/journal.pone.0033213)
Supplement: Figure S10 — Pyrosequencing results of candidate genomic regions in AML patients, AML cell lines and NBMs. (a) SPHKAP, (b) DPP6, (c) ST6GAL2, (d) HHEX and (e) Alu repeat. N refers to the number of samples tested for each investigated genomic region. Kruskal-Wallis test showed significant methylation difference among the groups (P<0.0001) for all tested genes and repeat. Dunn's multiple comparison tests showed that there was significant methylation difference between AML patients and NBMs in SPHKAP and DPP6 (P<0.05). Also, there was significant methylation difference between AML samples and AML cell lines in all investigated genes (P<0.05) except in the repeat. (DOC) [file pone.0033213.s011.doc]

**Figure S10 Pyrosequencing results of candidate genomic regions in AML patients, AML cell lines and NBMs.** (a) *SPHKAP,* (b) *DPP6,* (c) *ST6GAL2,* (d) *HHEX* and (e) Alu repeat. N refers to the number of samples tested for each investigated genomic region. Kruskal-Wallis test showed significant methylation difference among the groups (P < 0.0001) for all tested genes and repeat. Dunn’s multiple comparison tests showed that there was significant methylation difference between AML patients and NBMs in *SPHKAP* and *DPP6* (P < 0.05). Also, there was significant methylation difference between AML samples and AML cell lines in all investigated genes (P < 0.05) except in the repeat.

a.

**
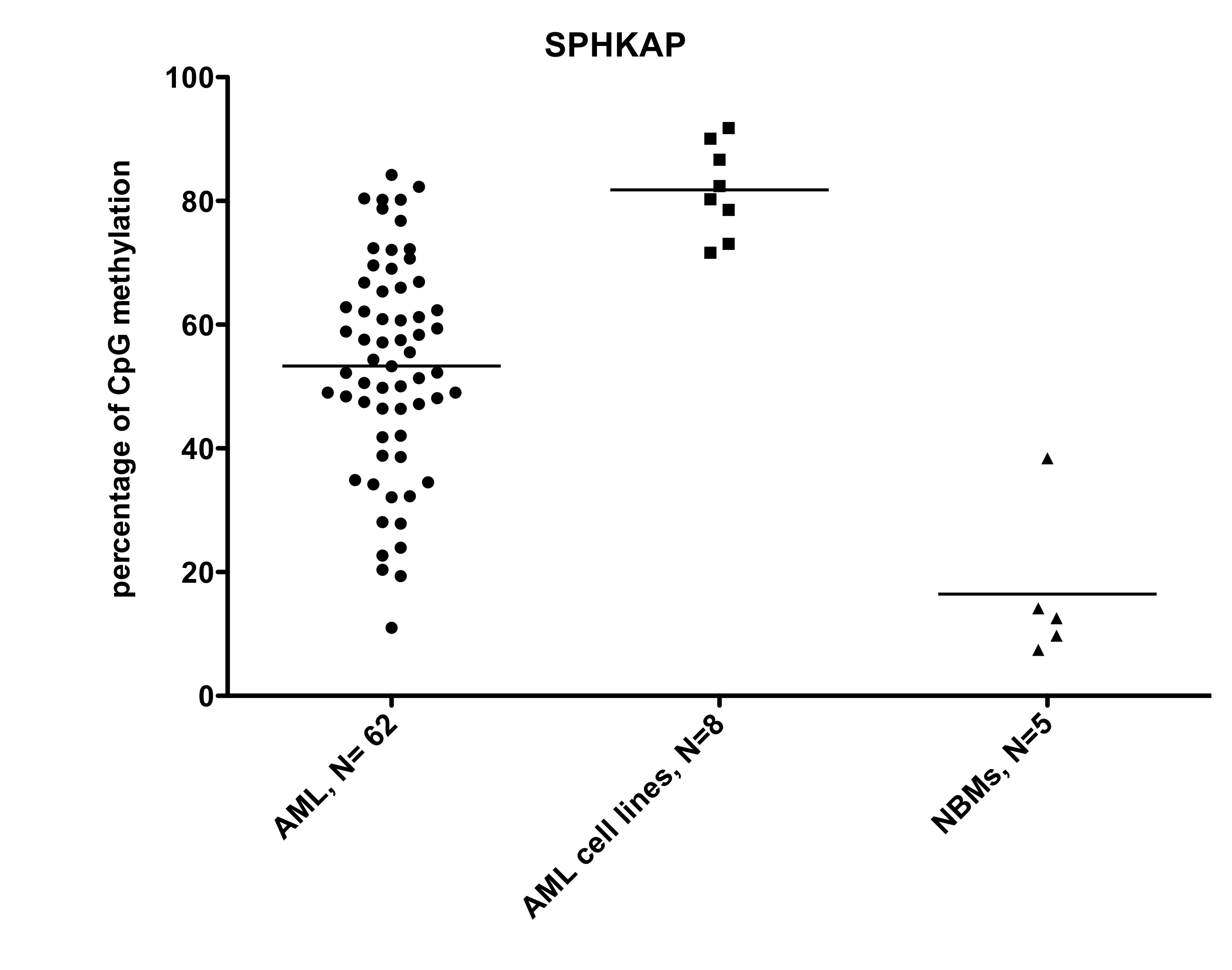
**

b.

**
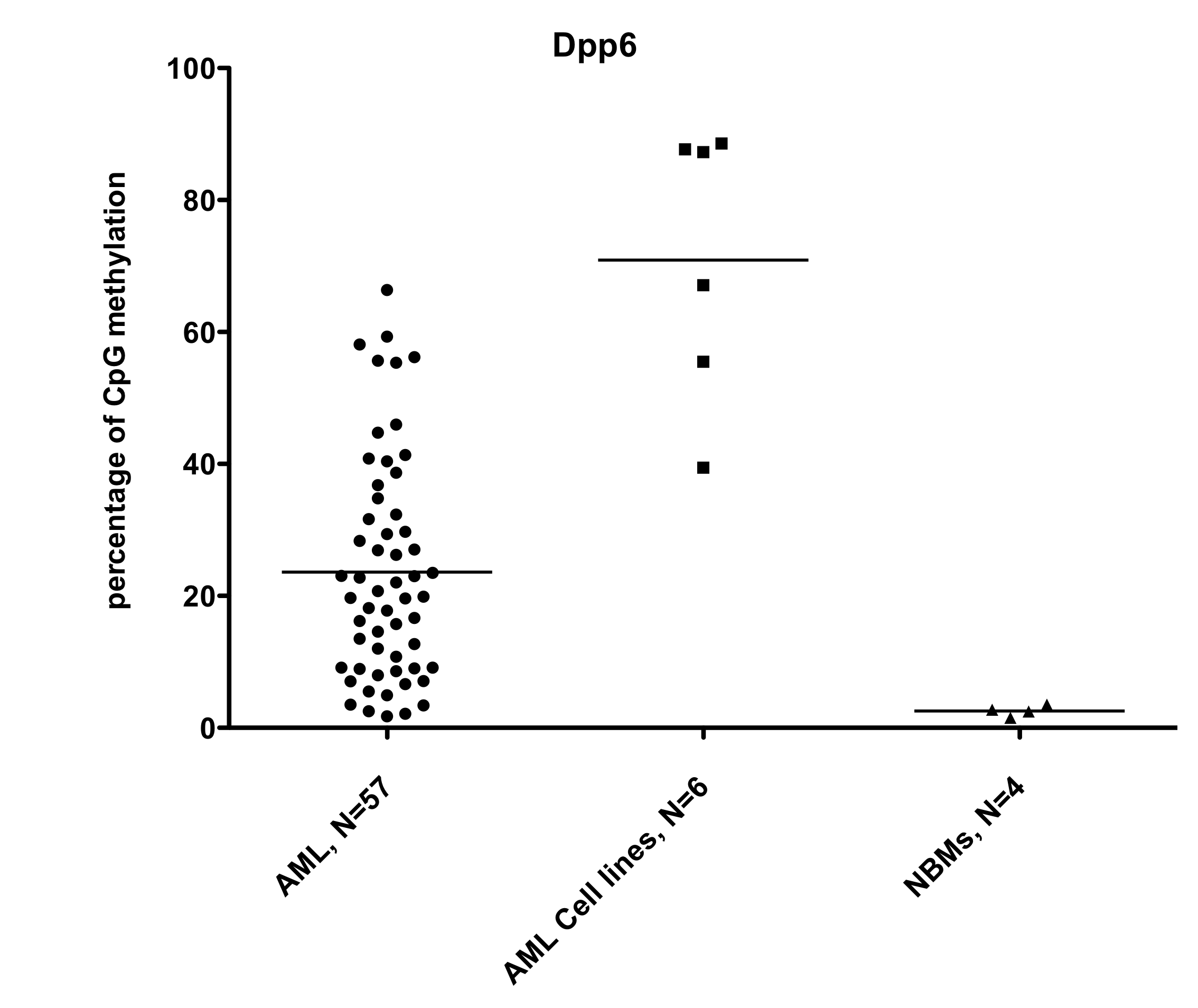
**

c.

**
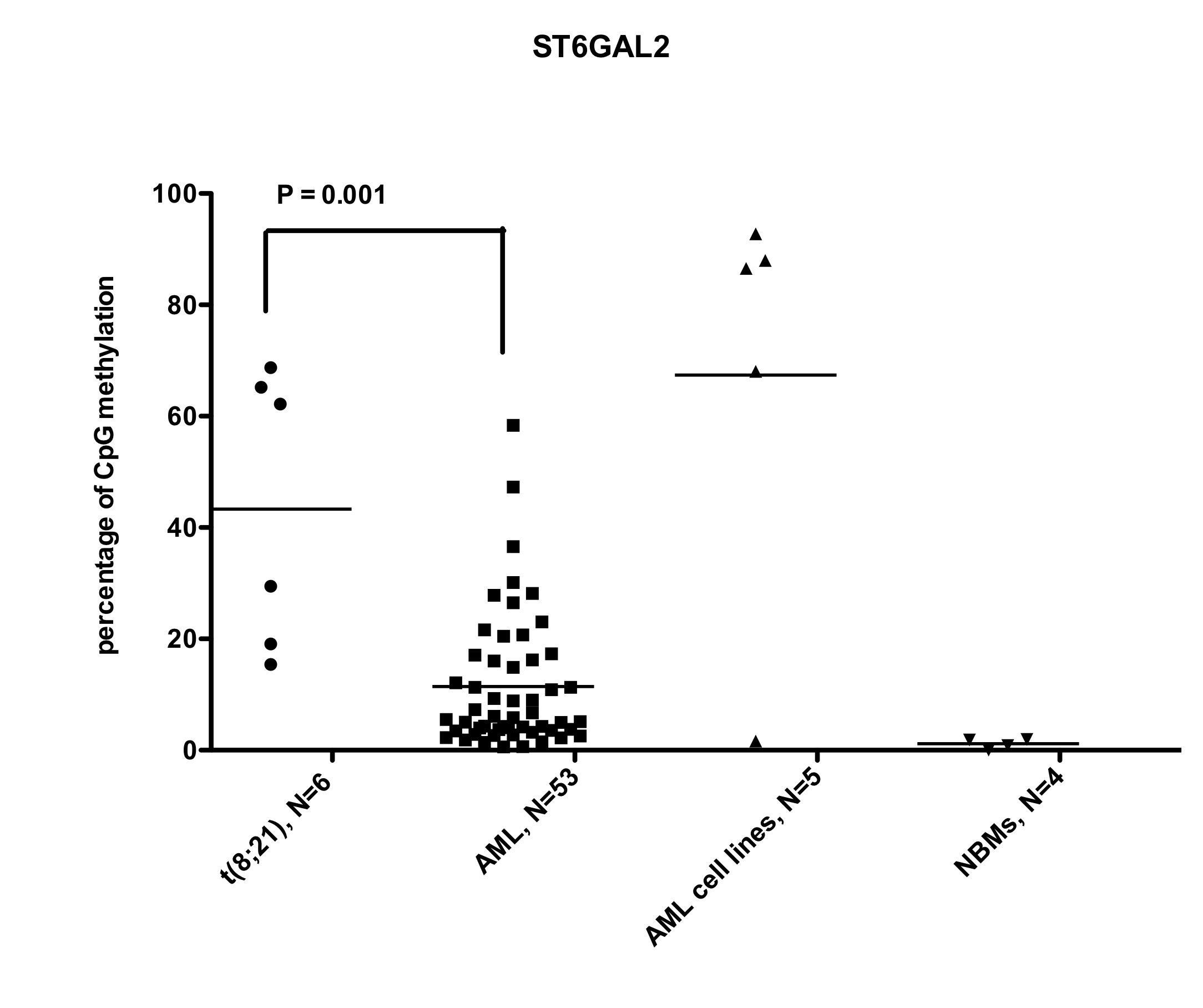
**

d.

**
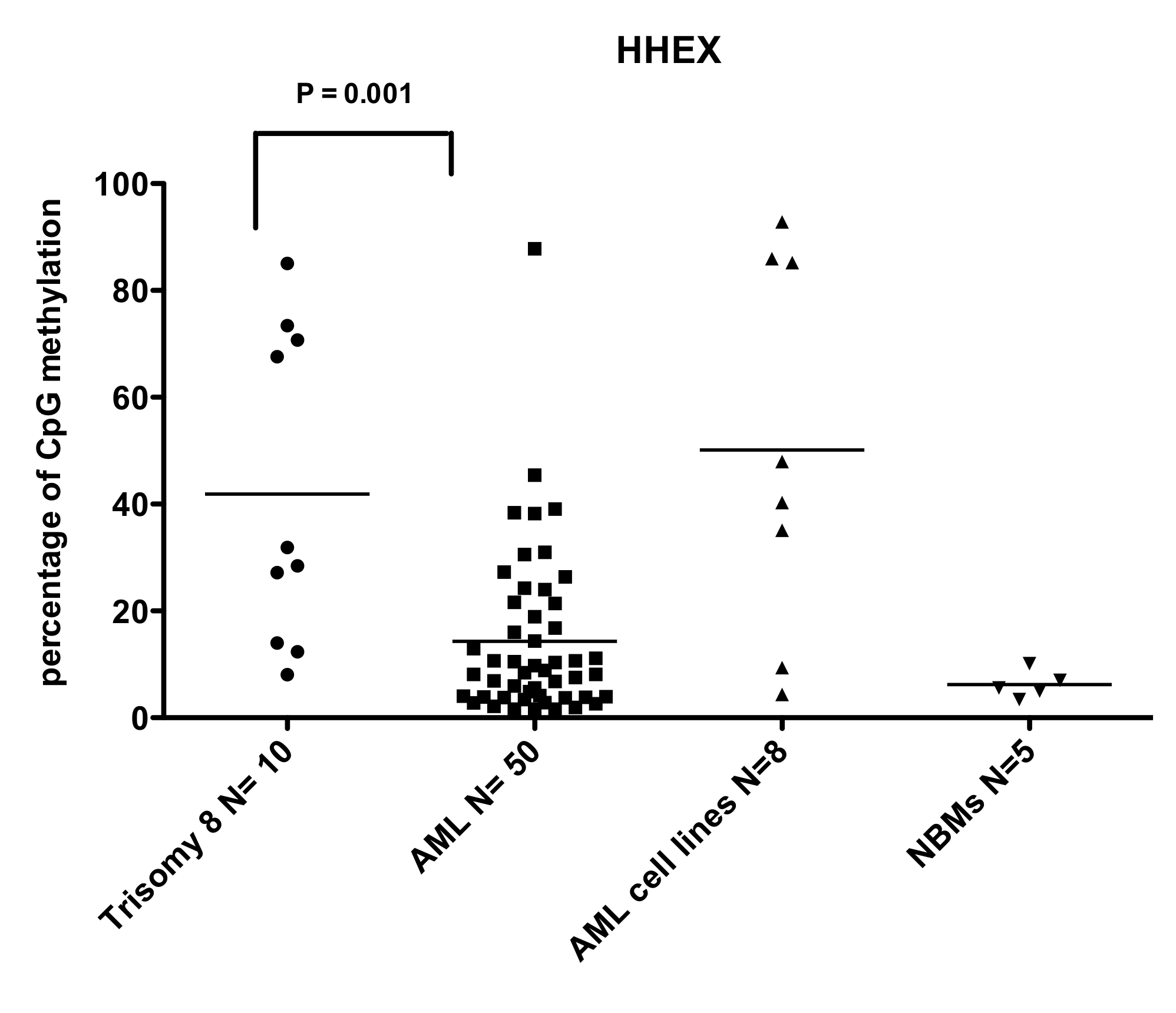
**

e.

**
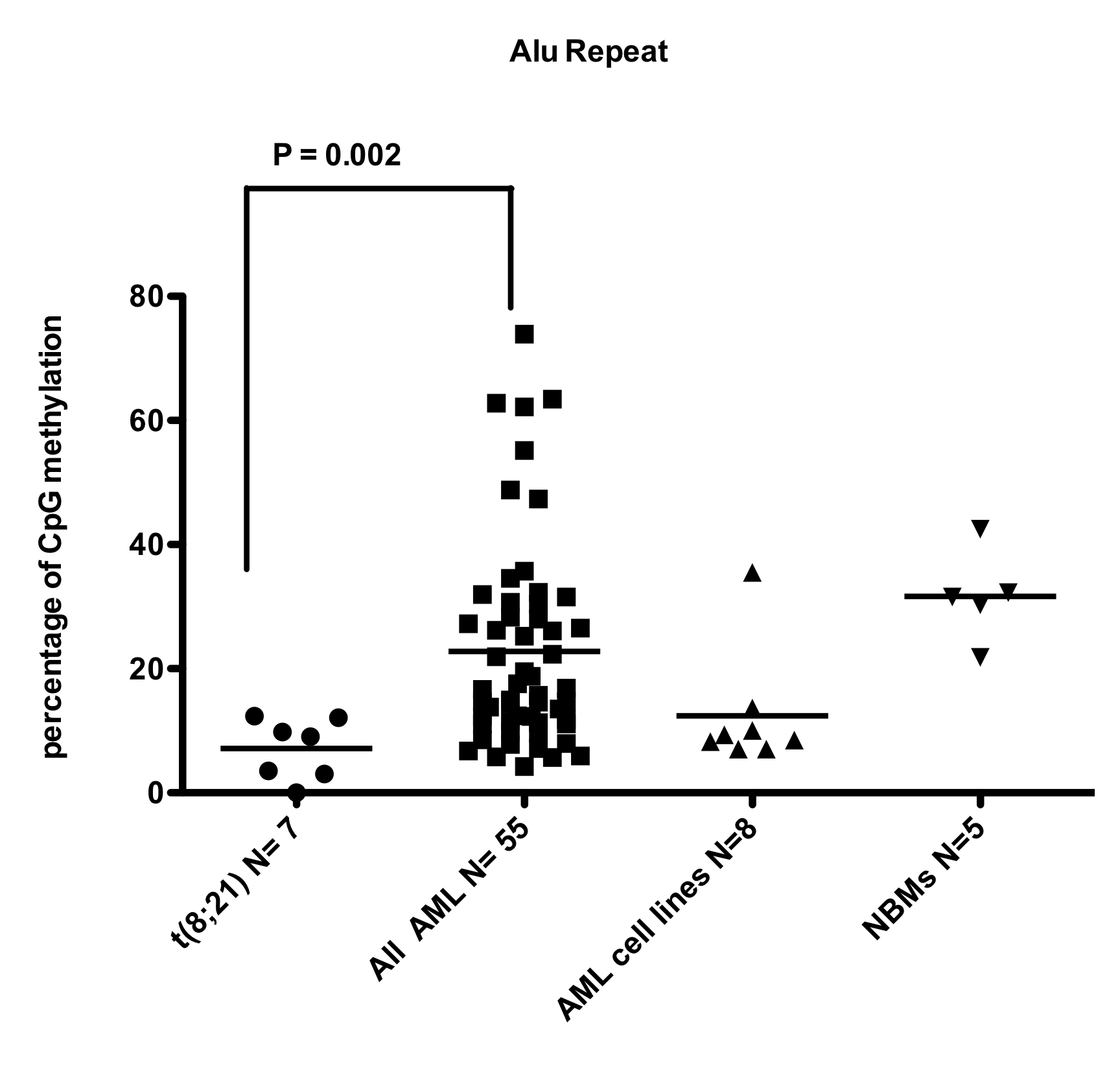
**
