# Supplementary material for: Genome Wide Analysis of Acute Myeloid Leukemia Reveal Leukemia Specific Methylome and Subtype Specific Hypomethylation of Repeats
Source: PLoS One. 2012 Mar 29;7(3):e33213. doi: 10.1371/journal.pone.0033213 (PMC3315563; doi:10.1371/journal.pone.0033213)
Supplement: Figure S11 — HHEX gene methylation and expression assay. (a) HHEX gene methylation (a significant methylated CGI located within the body of HHEX gene) among different AML patients. (b) HHEX gene expression among different AML patients. (c) Effect of DAC on HHEX expression in AML cell lines. HHEX gene expression was measured relative to NBM, PB = peripheral blood from healthy donors. (DOC) [file pone.0033213.s012.doc]

**Figure S11 *HHEX* gene methylation and expression assay.** (a) *HHEX* gene methylation (a significant methylated CGI located within the body of *HHEX* gene) among different AML patients. (b) *HHEX* gene expression among different AML patients. (c) Effect of DAC on *HHEX* expression in AML cell lines. *HHEX* gene expression was measured relative to NBM, PB = peripheral blood from healthy donors.

a.

b.

c.
