# Supplementary material for: Genome Wide Analysis of Acute Myeloid Leukemia Reveal Leukemia Specific Methylome and Subtype Specific Hypomethylation of Repeats
Source: PLoS One. 2012 Mar 29;7(3):e33213. doi: 10.1371/journal.pone.0033213 (PMC3315563; doi:10.1371/journal.pone.0033213)
Supplement: Figure S12 — Histograms of uncorrected P values after testing the equality of the methylation means between groups. (a) in 4 genomic regions and (b) in repeats. When investigating the data with equal means between groups, the P values were expected to be uniformly distributed across the unit interval (blue line). Comparing the distribution of uncorrected P values to the uniform distribution expected for random data revealed enrichment of P value<0.05 (red line) indicating differential methylation pattern. Satellites did not show a specific distribution of uncorrected P values across the samples. High frequencies of P values<0.05 across the samples were observed in the other tested repeats; SINEs, LINEs and LTRs. (DOC) [file pone.0033213.s013.doc]

**Figure S12. Histograms of uncorrected P values after testing the equality of the methylation means between groups.** (a) in 4 genomic regions and (b) in repeats. When investigating the data with equal means between groups, the P values were expected to be uniformly distributed across the unit interval (blue line). Comparing the distribution of uncorrected P values to the uniform distribution expected for random data revealed enrichment of P value < 0.05 (red line) indicating differential methylation pattern.

a.

**Promoters**


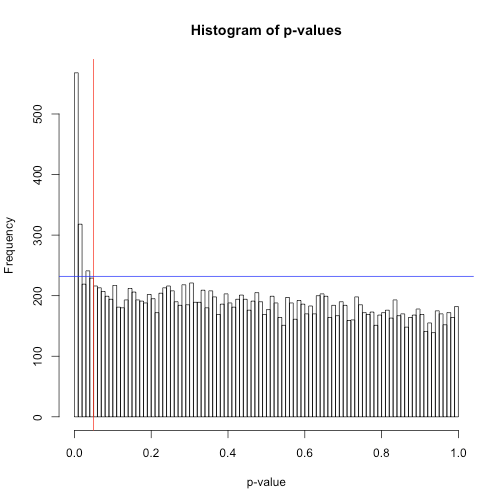

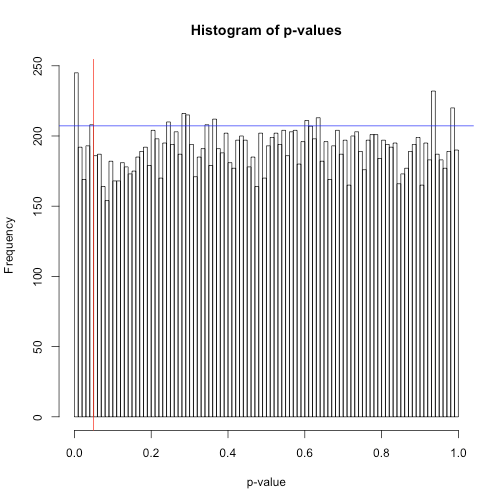

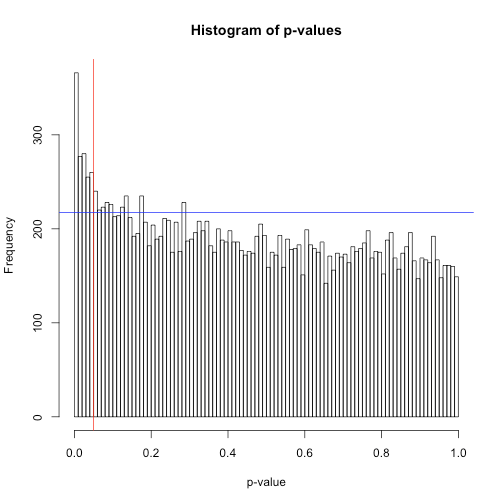

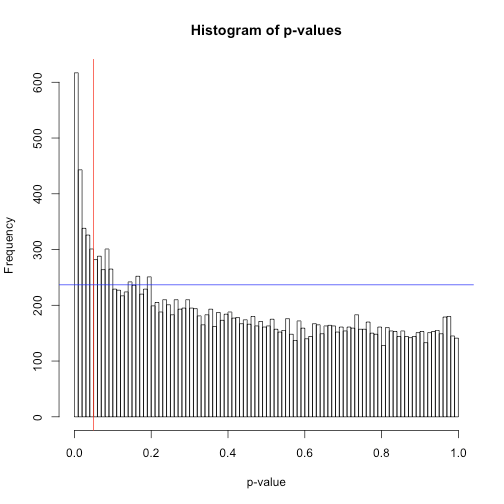

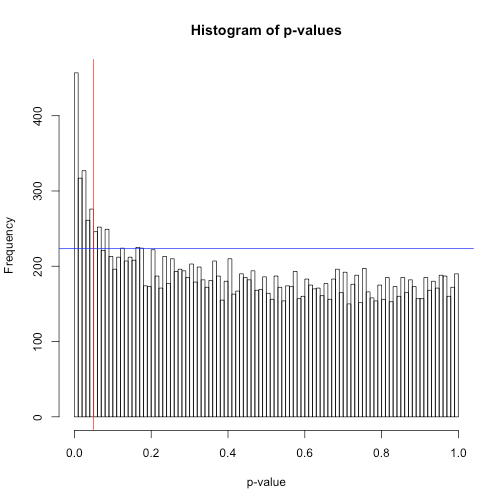


**Gene bodies**


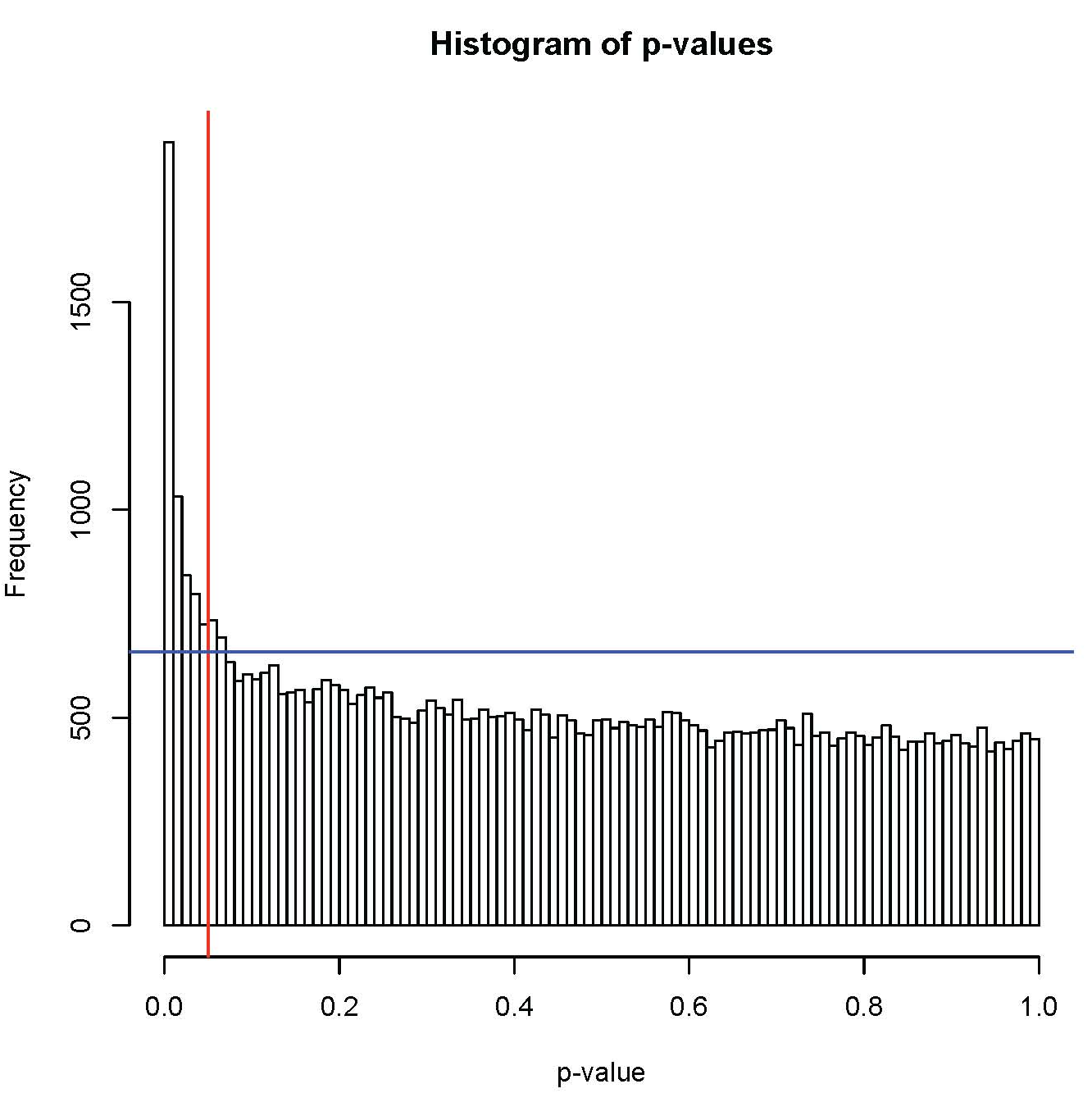

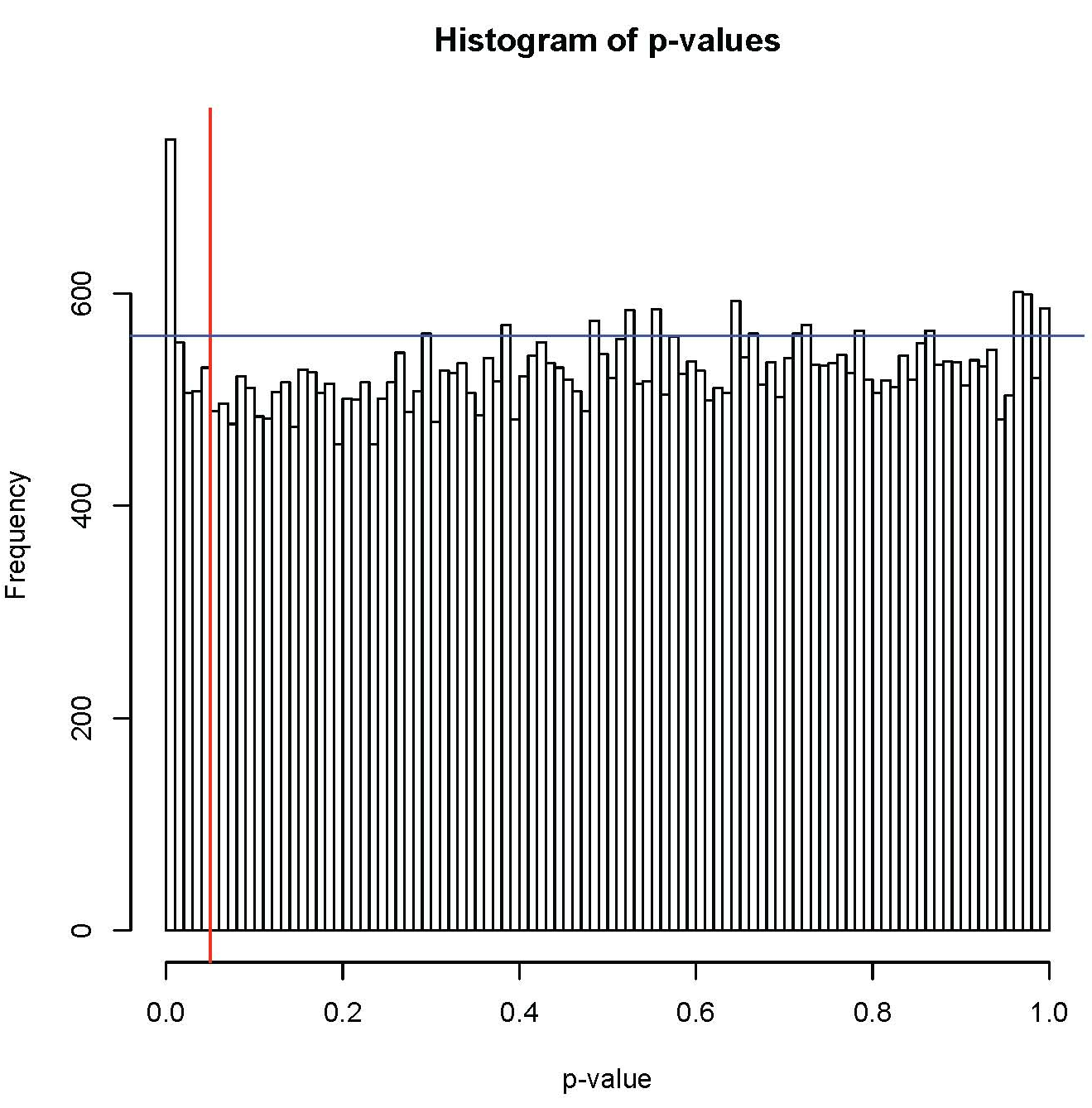

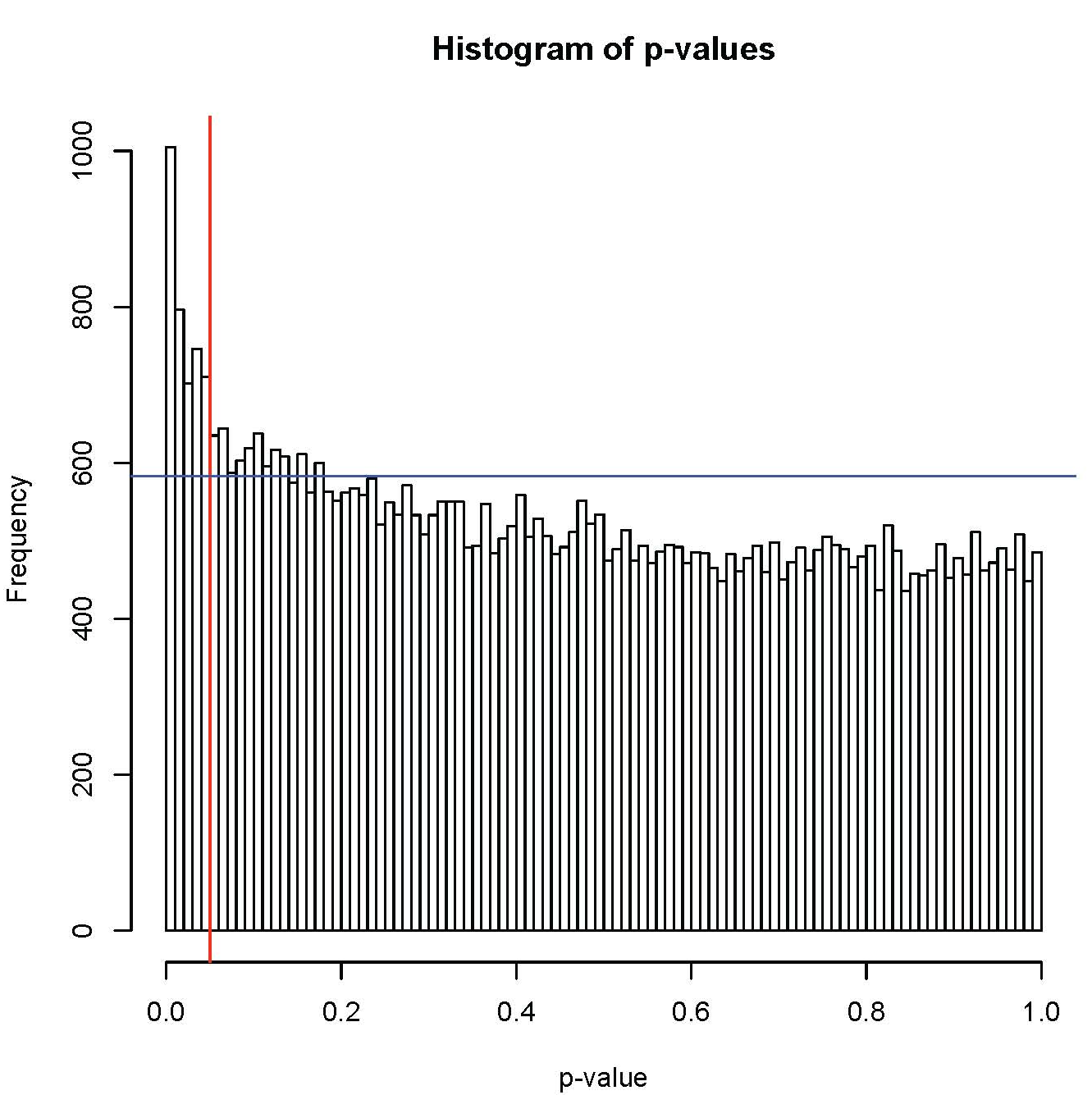

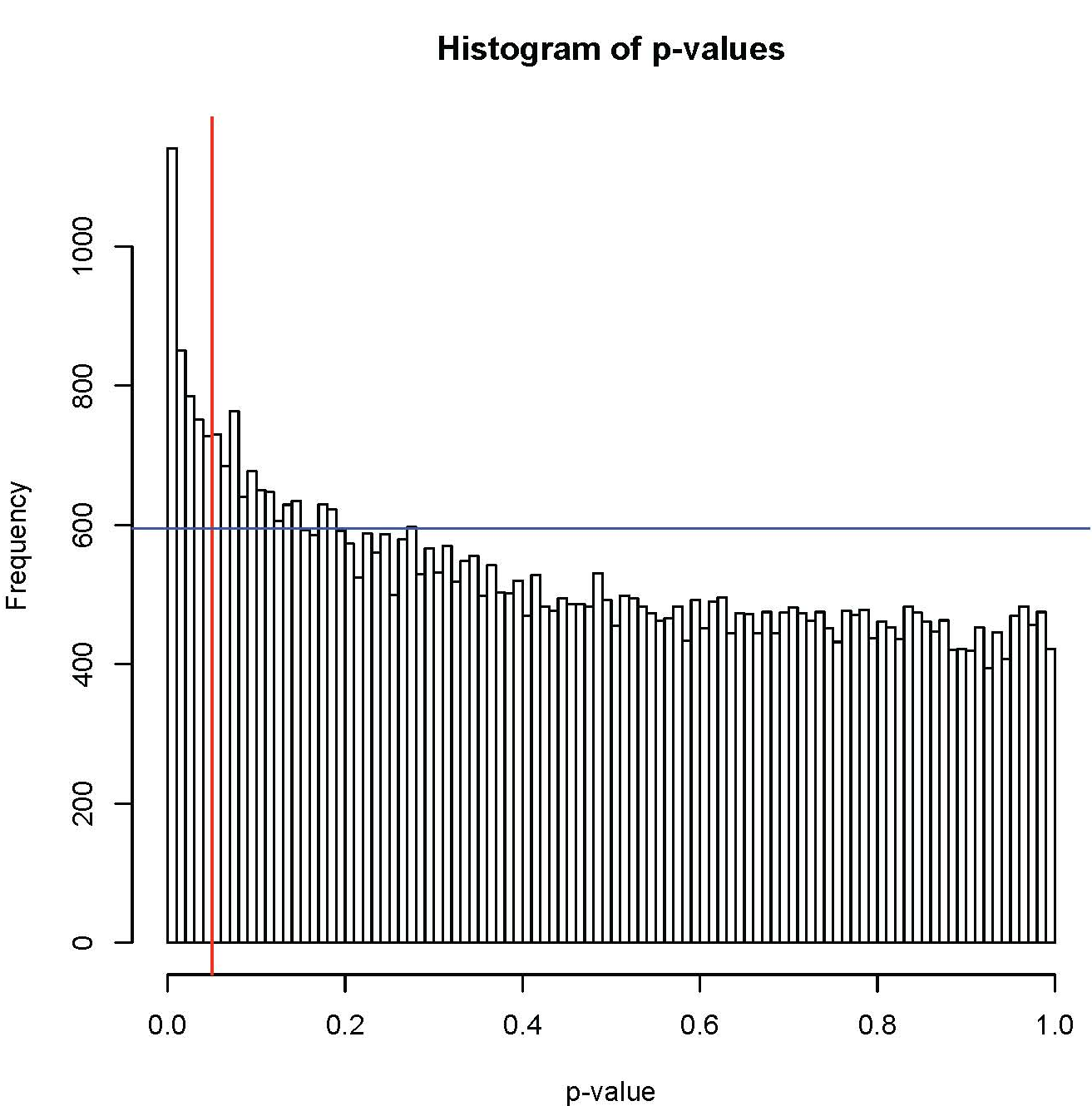

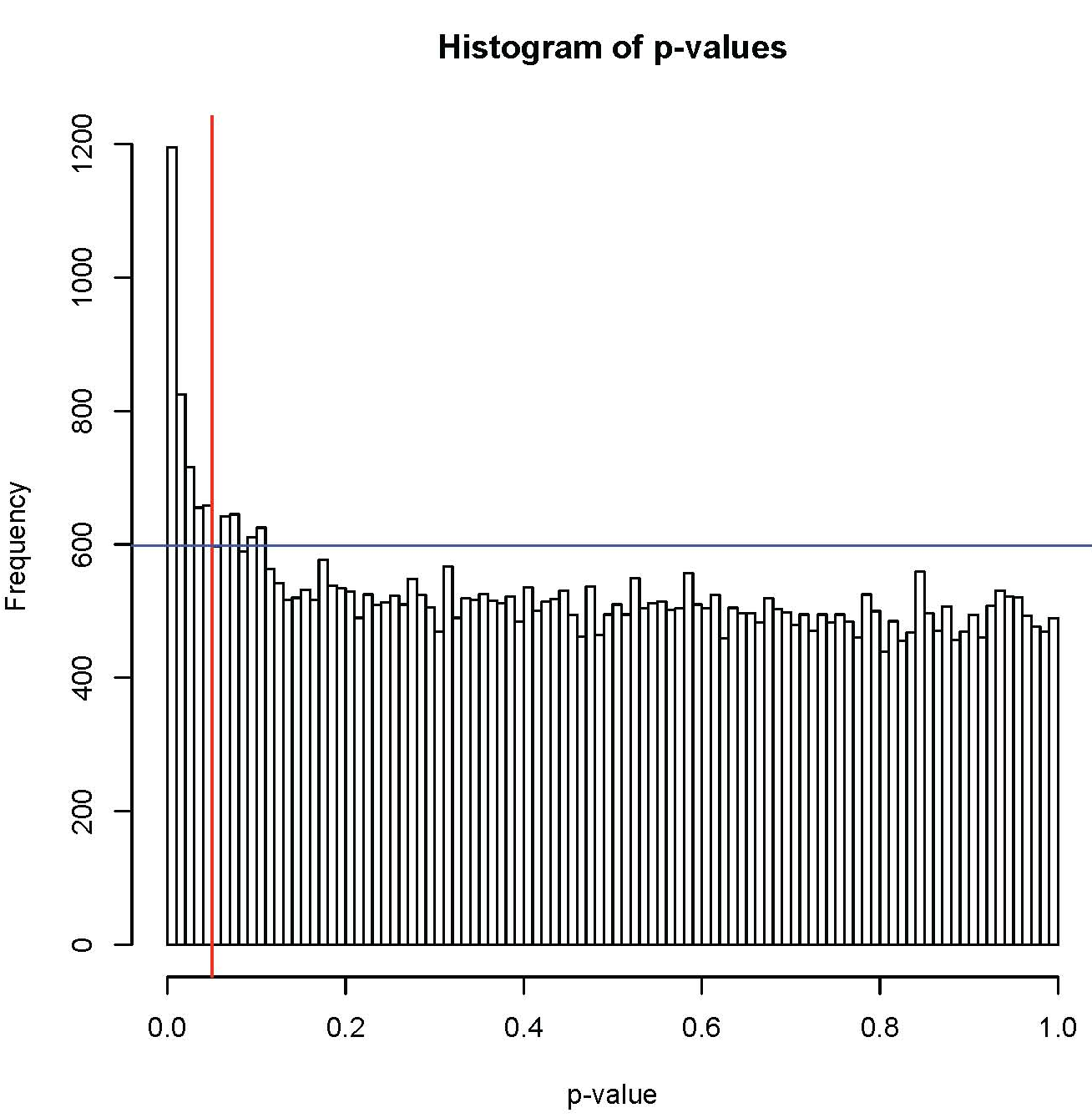


**CGIs**


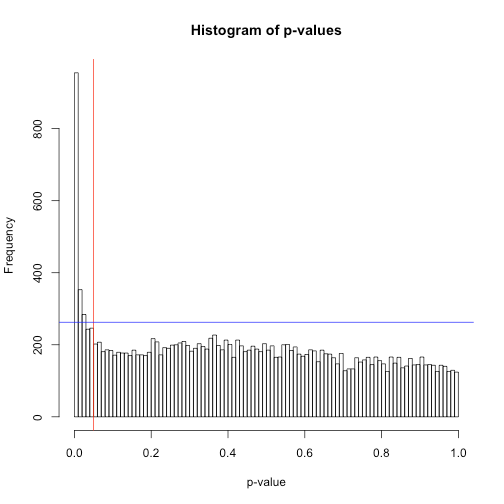

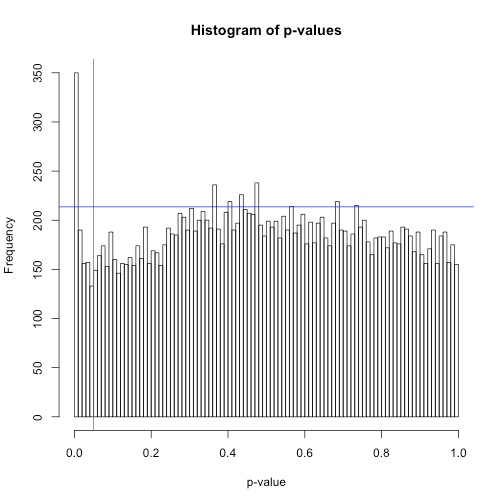

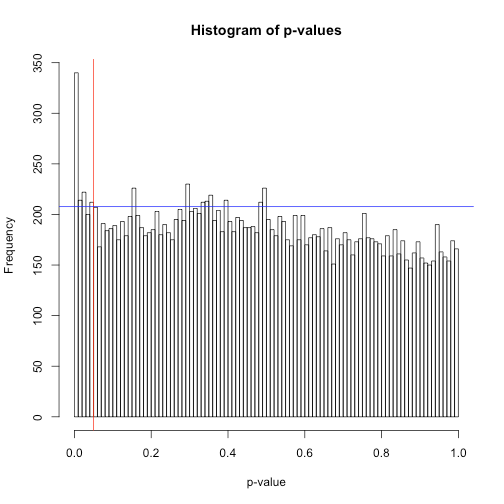

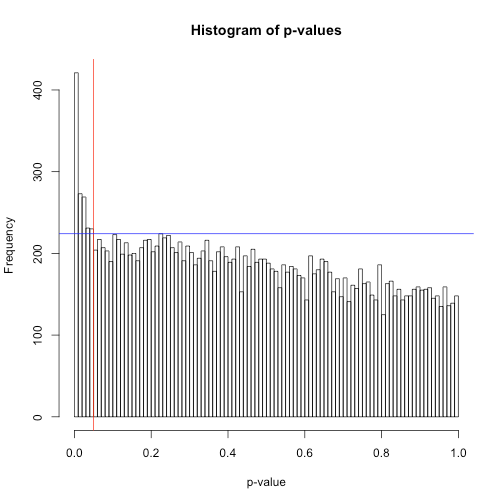

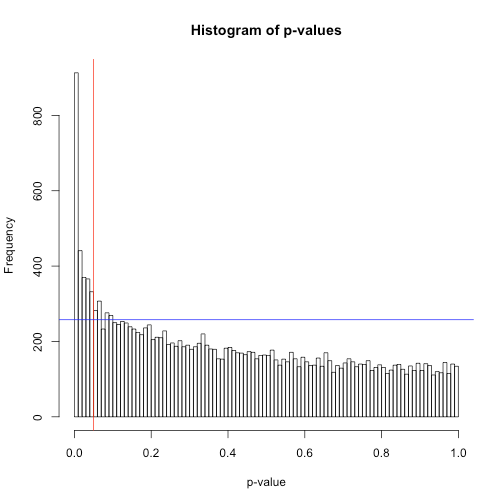


**CGI shores**


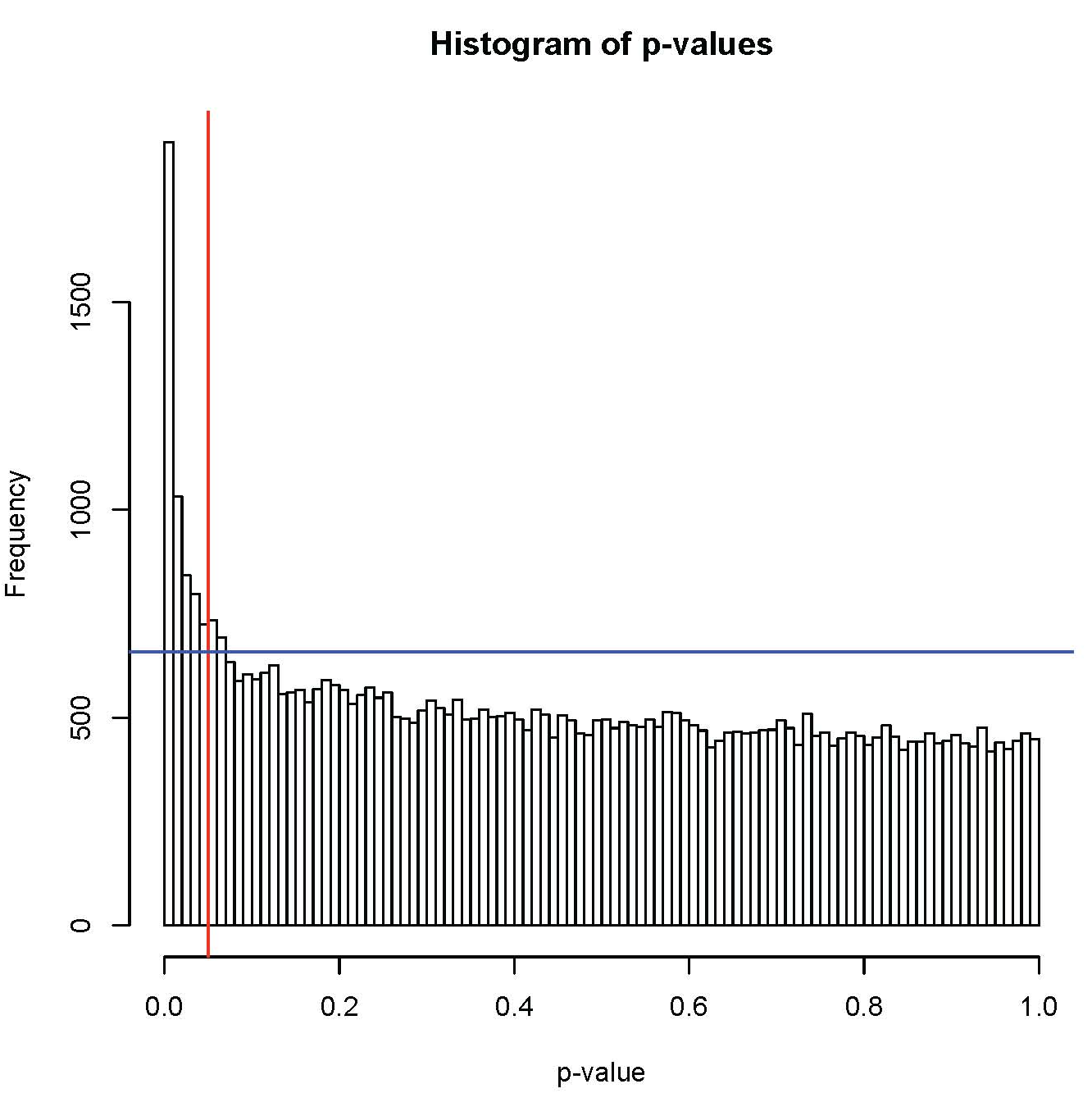

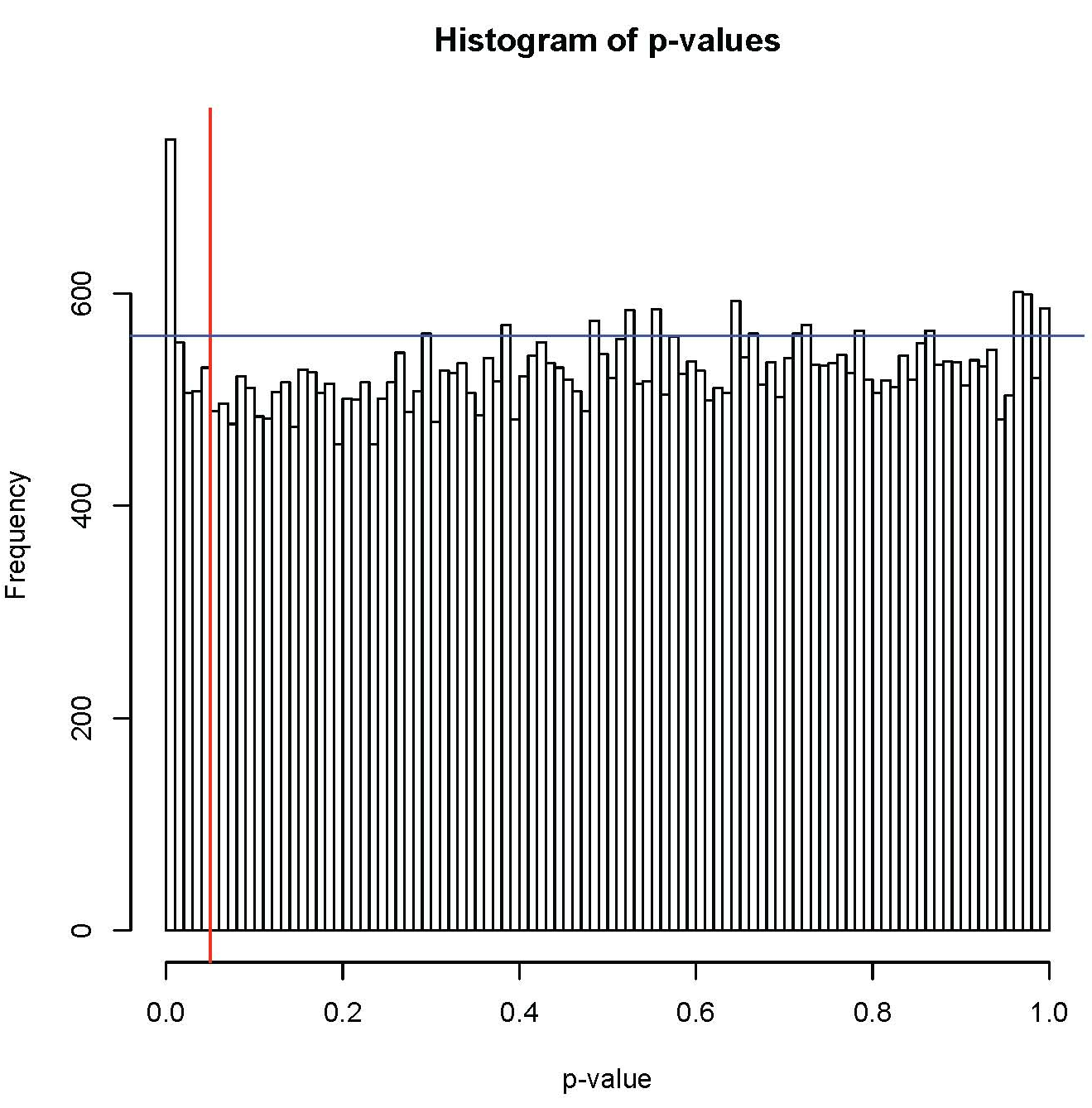

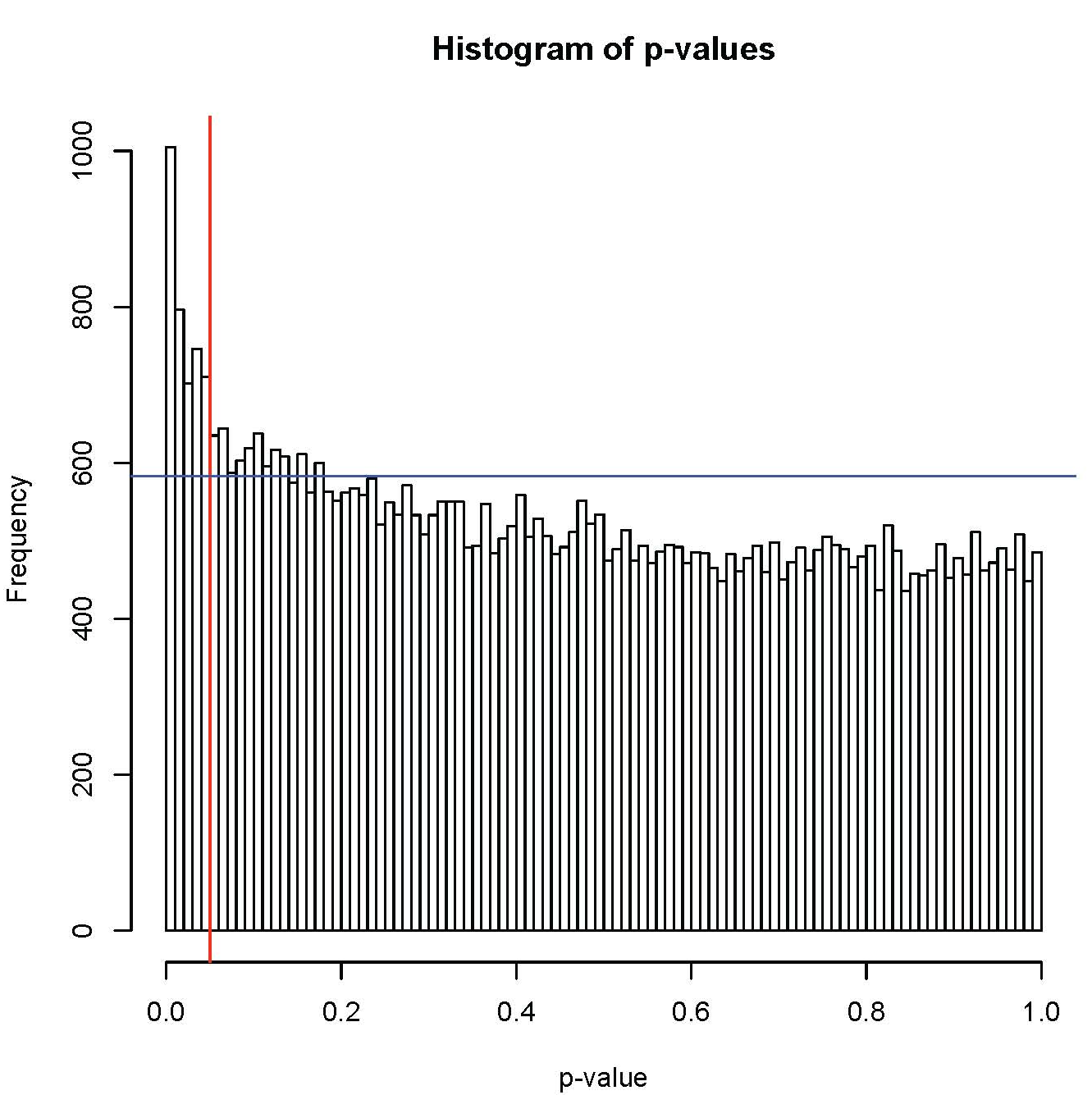

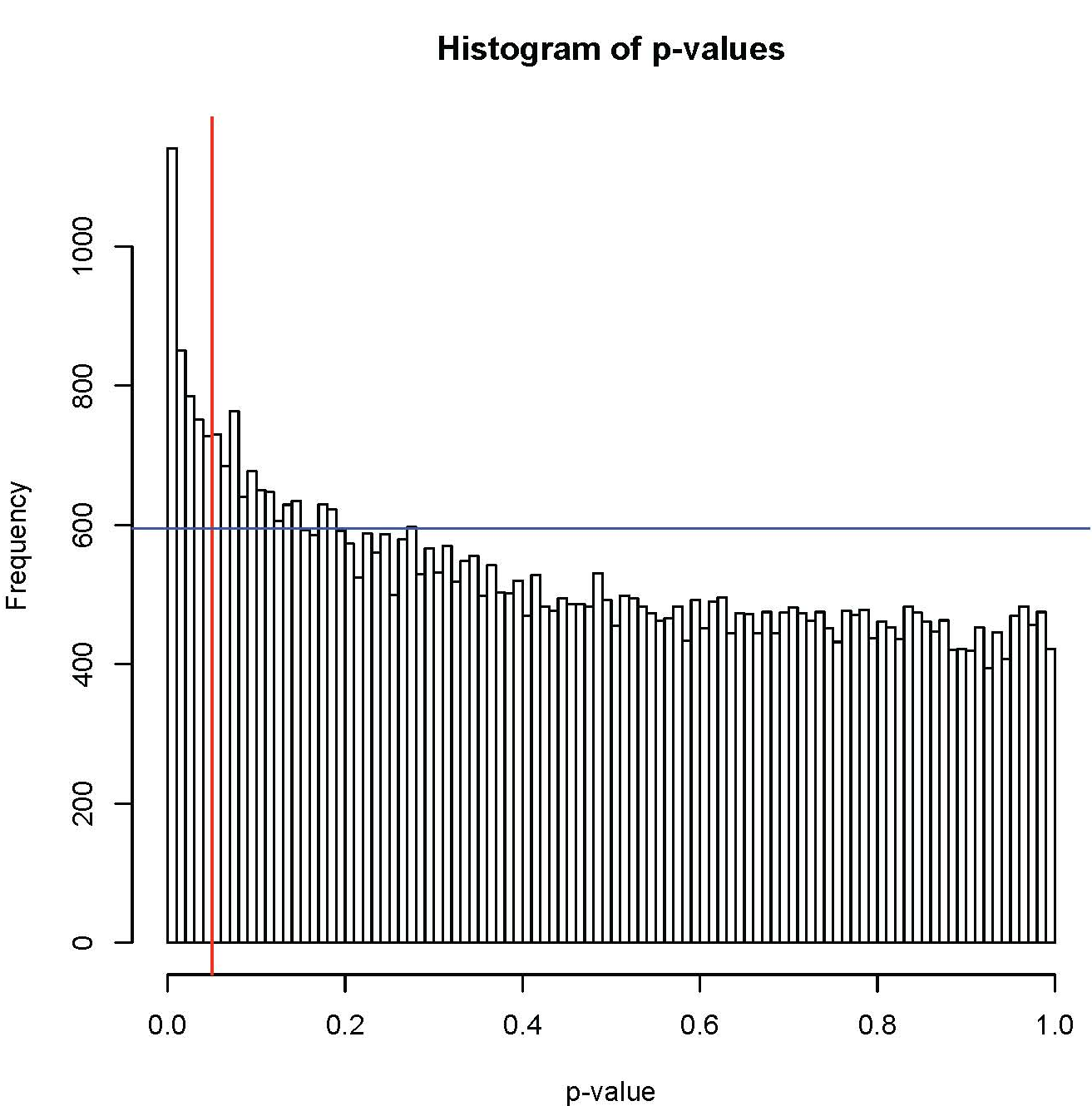

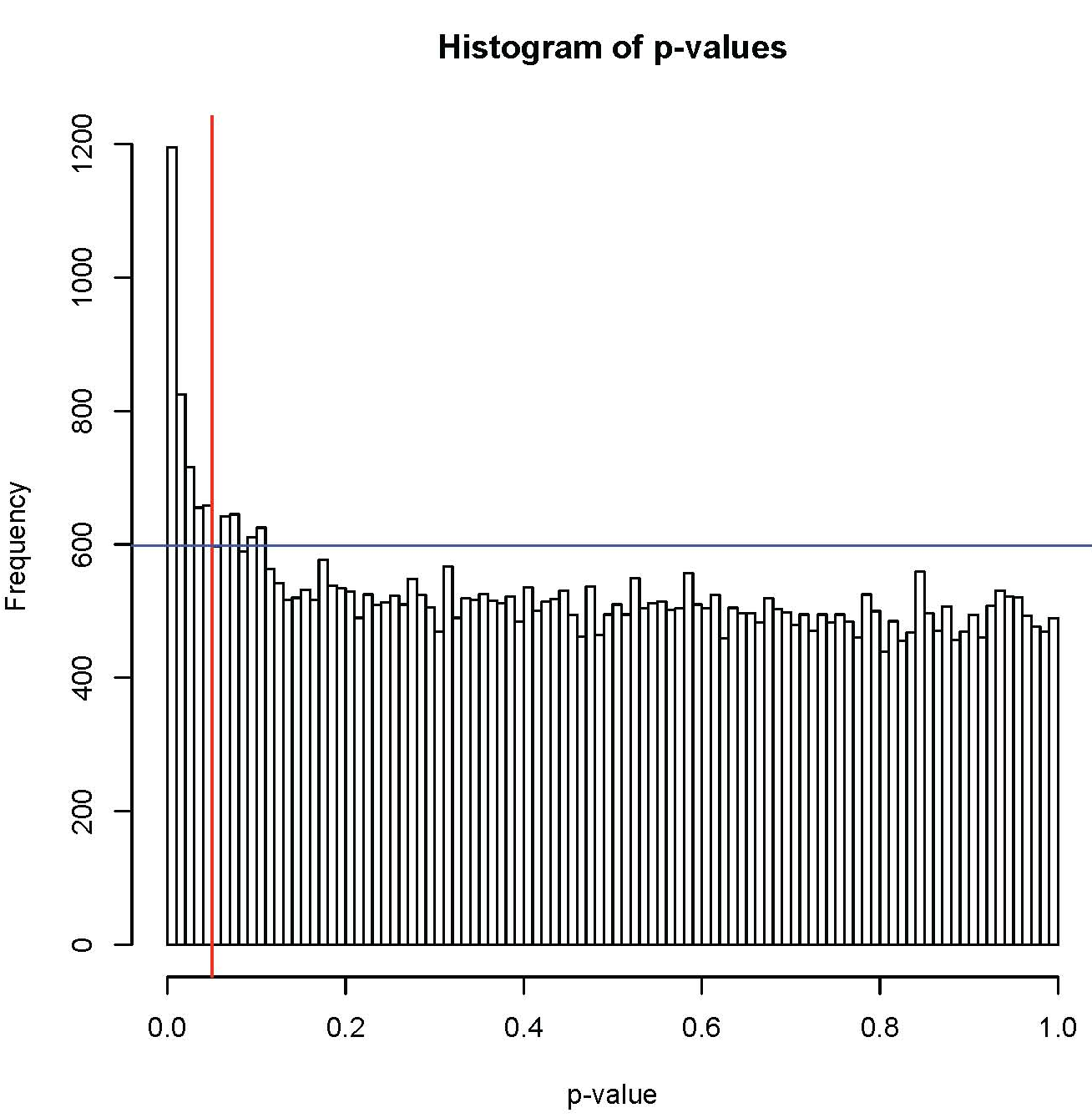


**t(8;21) versus t(15;17) versus NK versus Trisomy 8 versus All AMLs**

**all groups all groups all groups all groups versus all NBMs**

b. Satellites did not show a specific distribution of uncorrected P values across the samples. High frequencies of P values < 0.05 across the samples were observed in the other tested repeats; SINEs, LINEs and LTRs.

**Satellites**


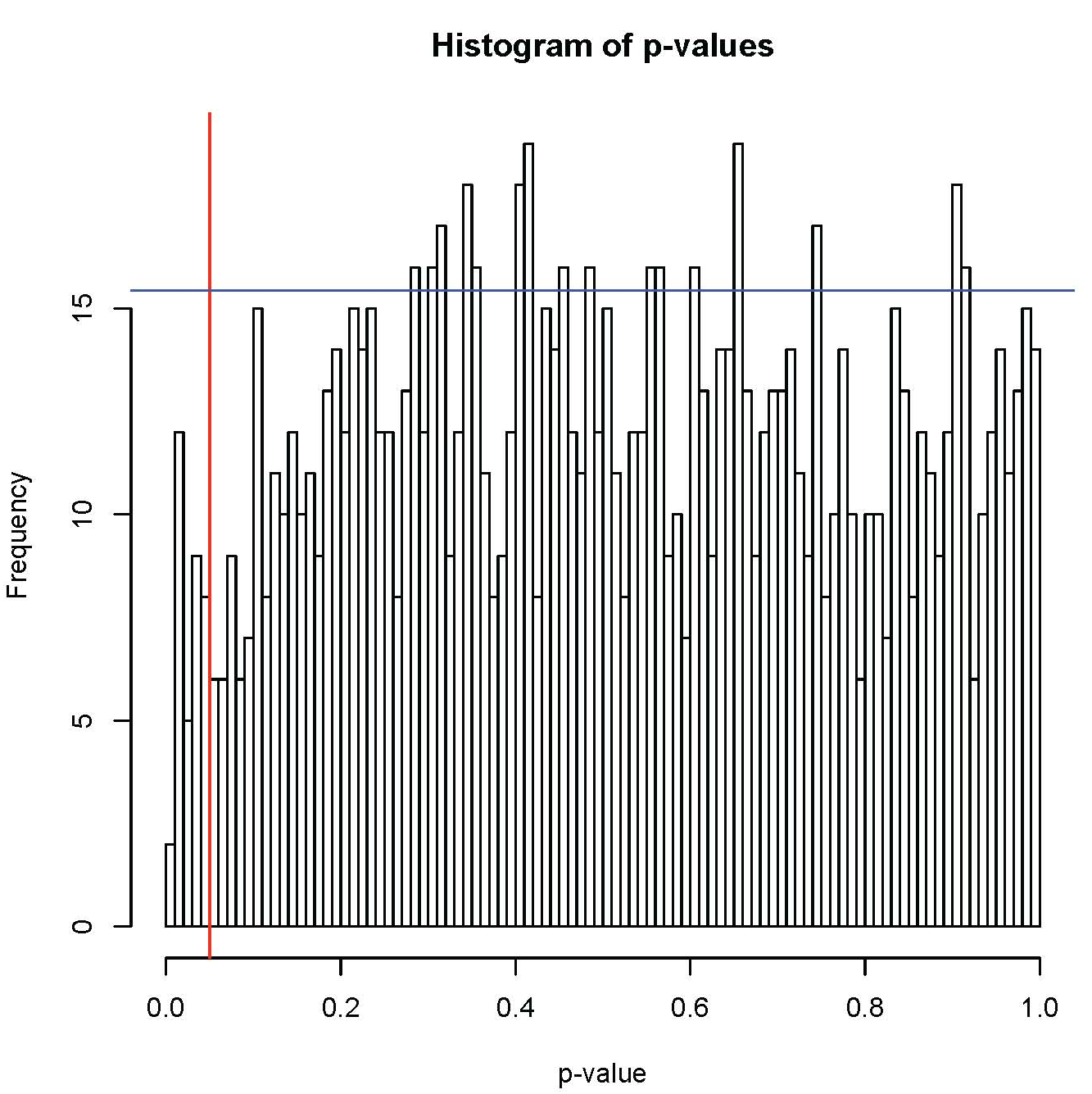

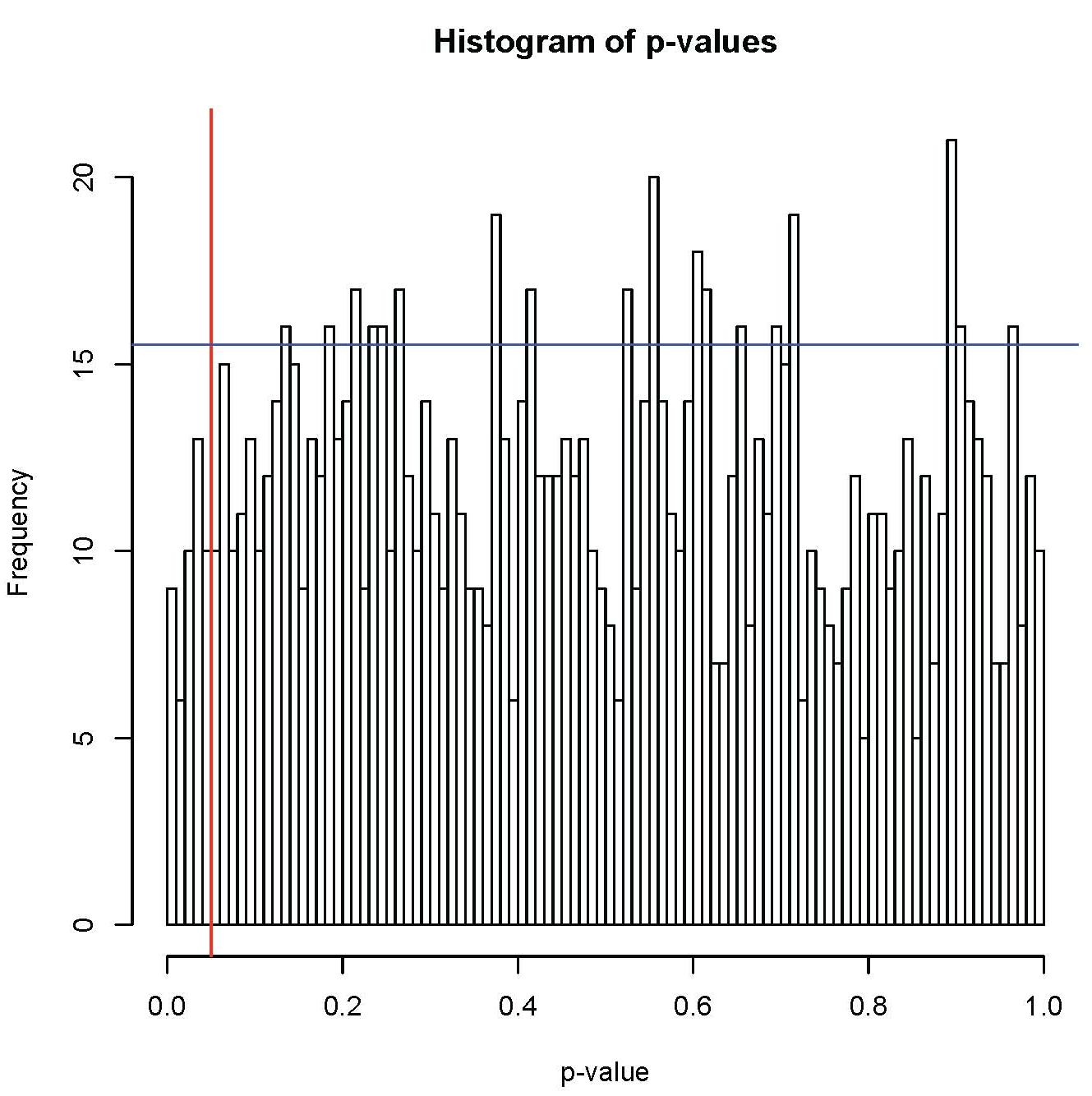

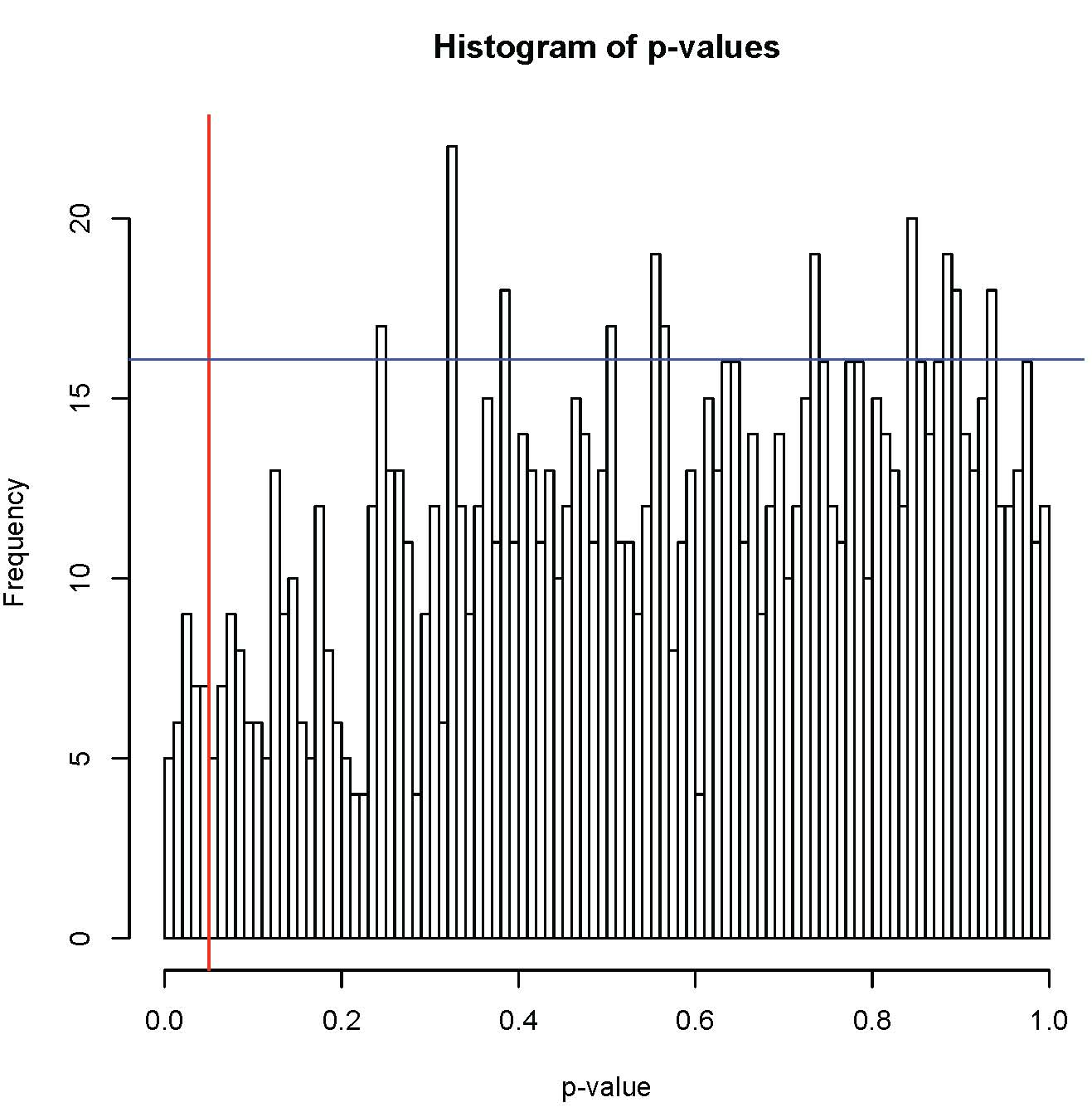

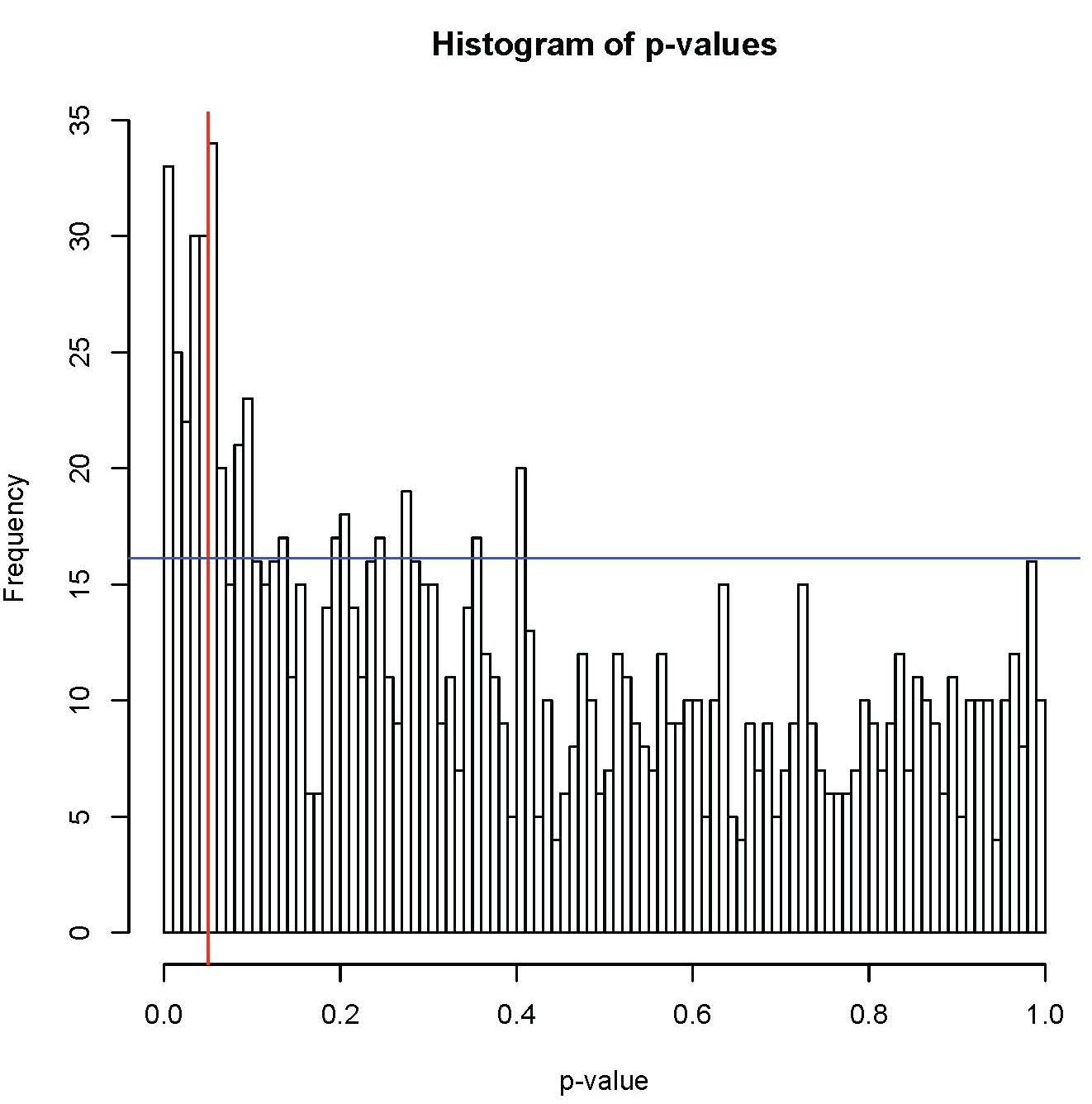

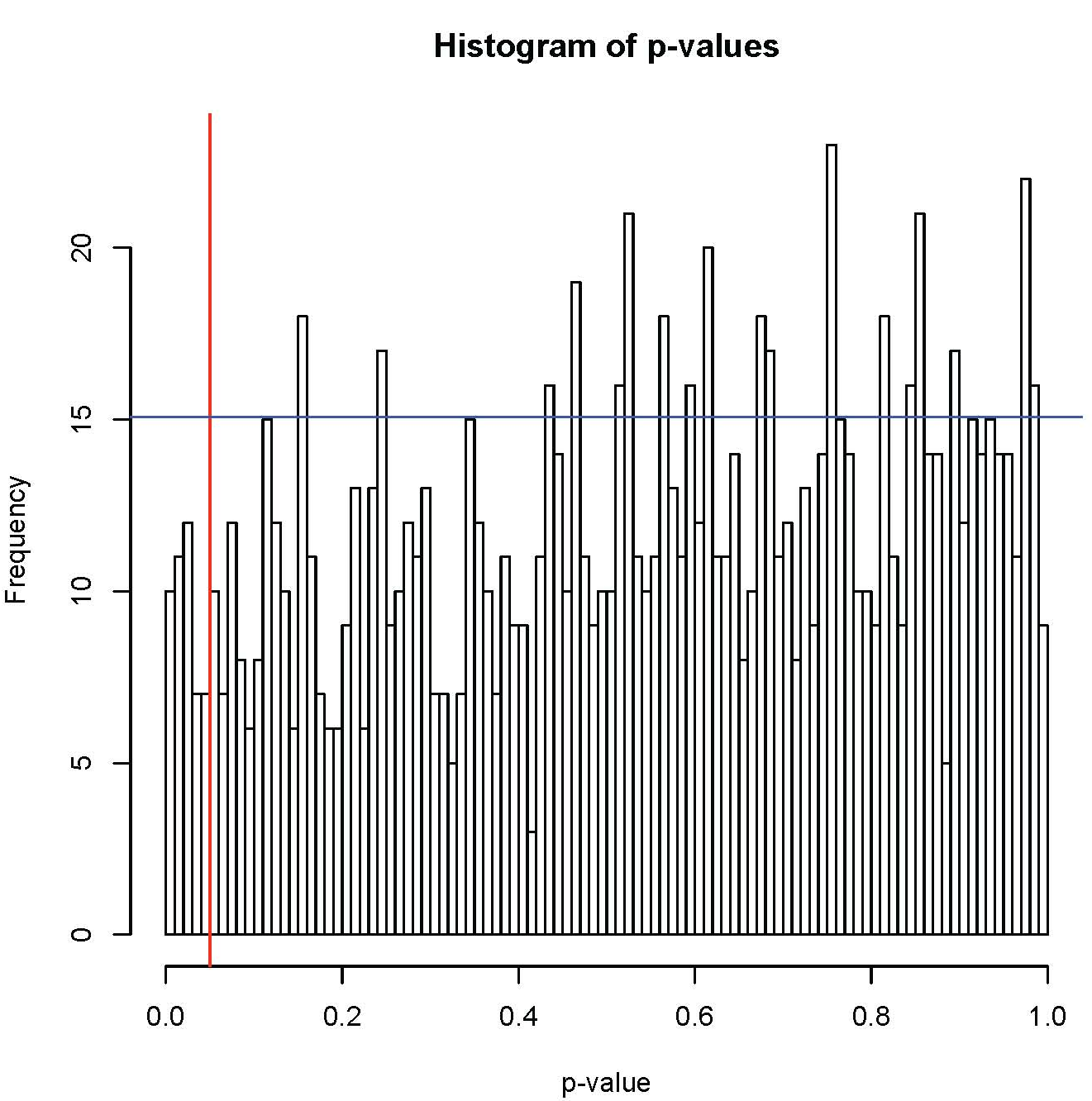


**SINEs**


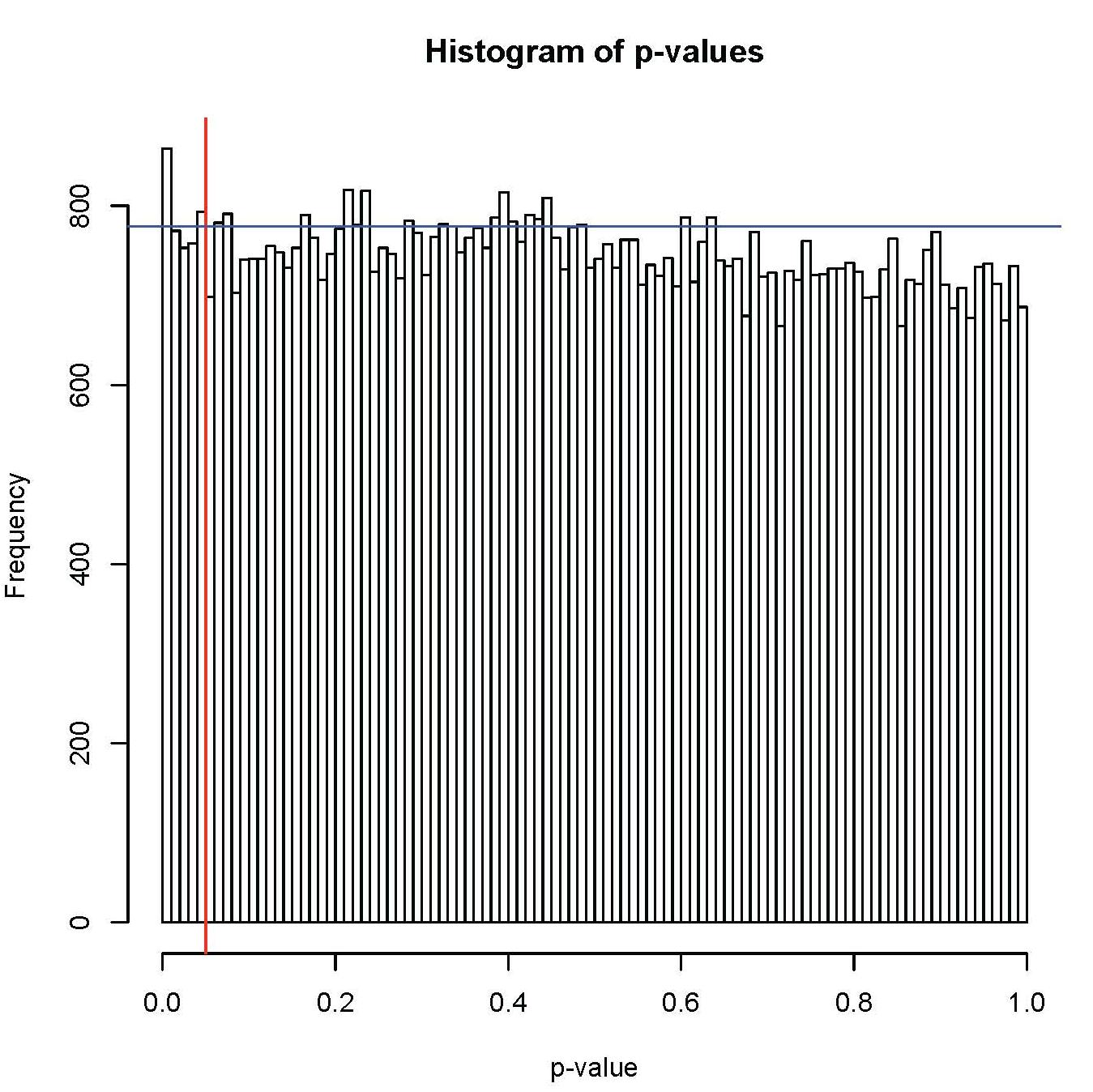

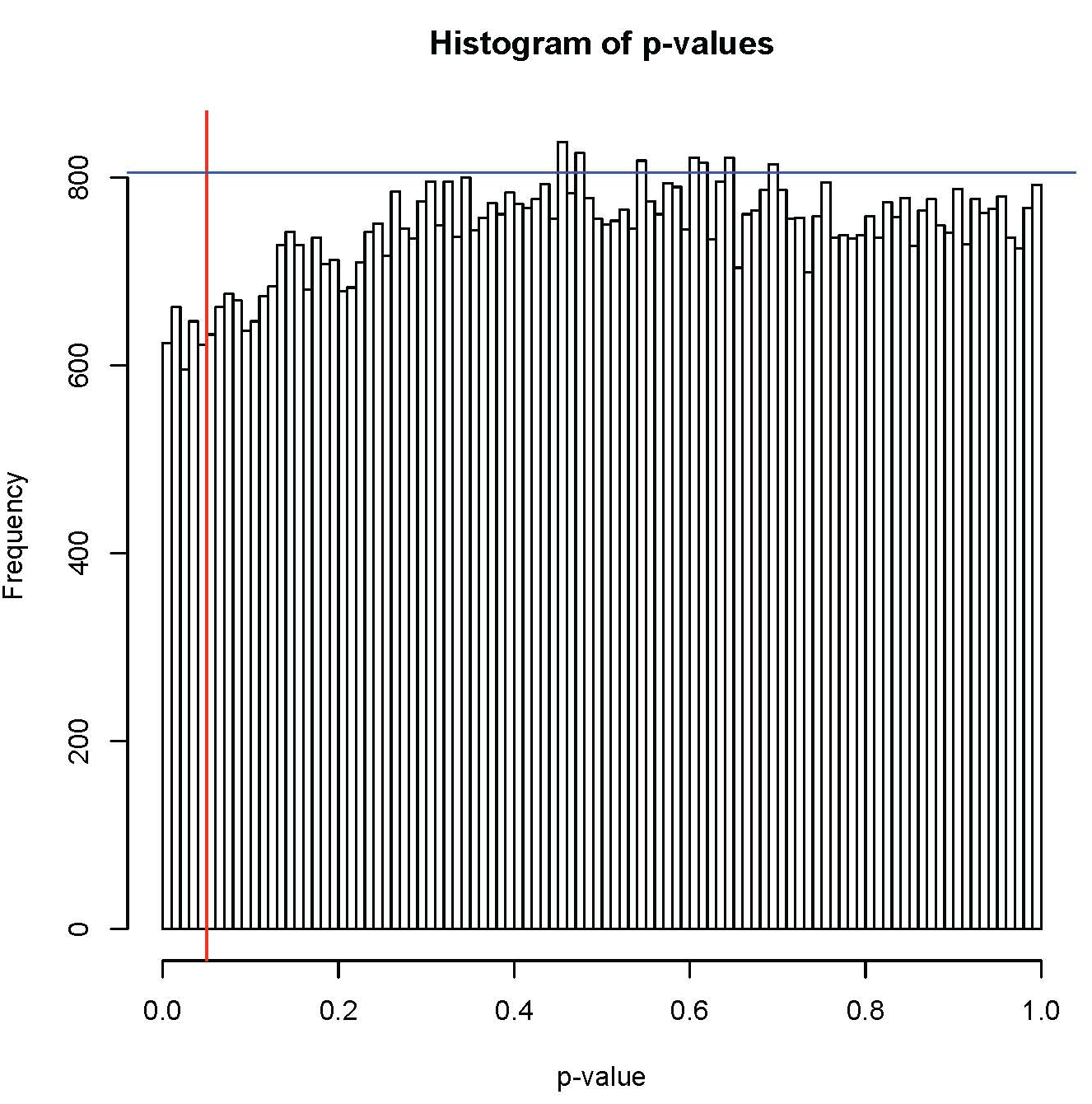

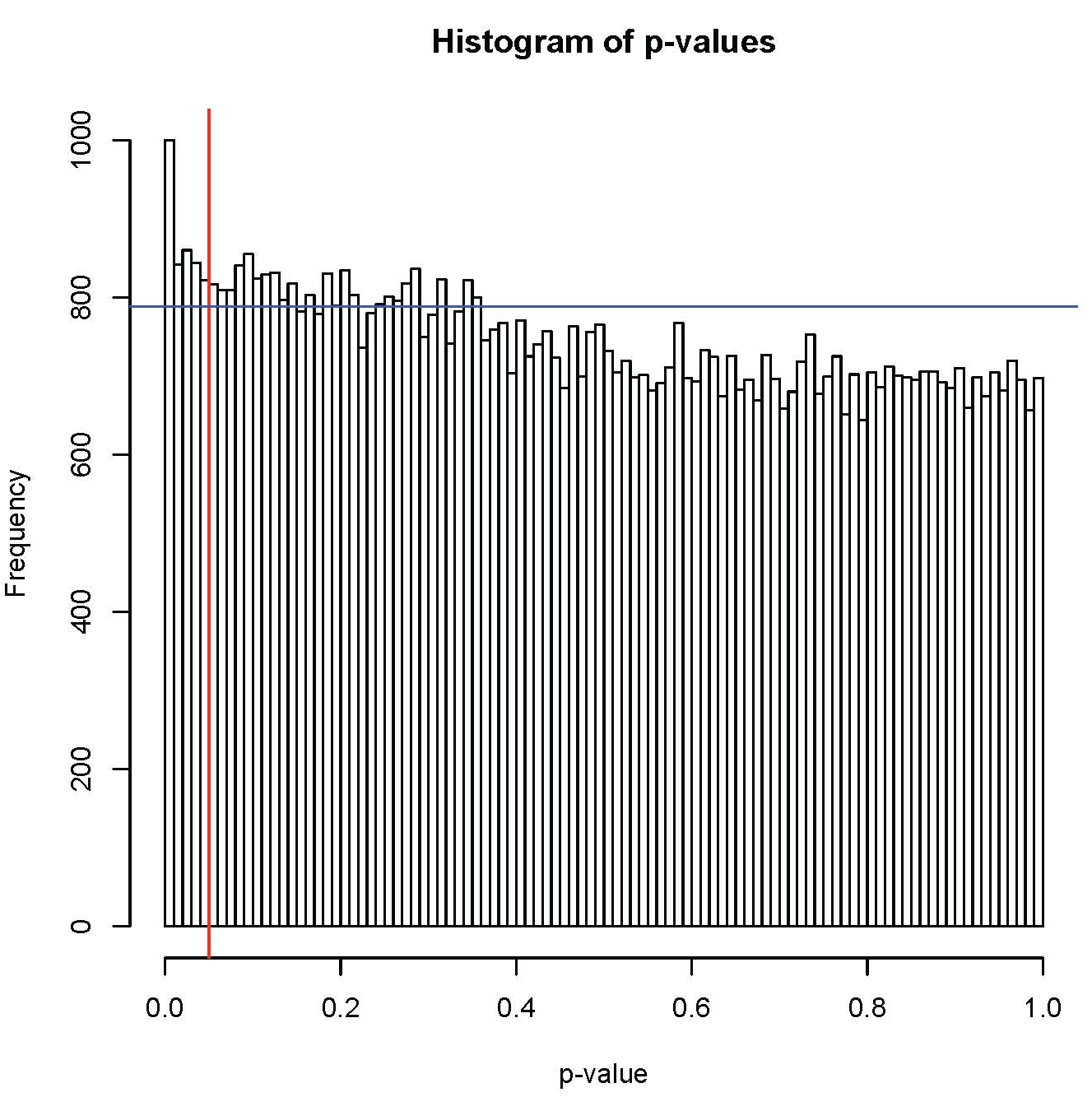

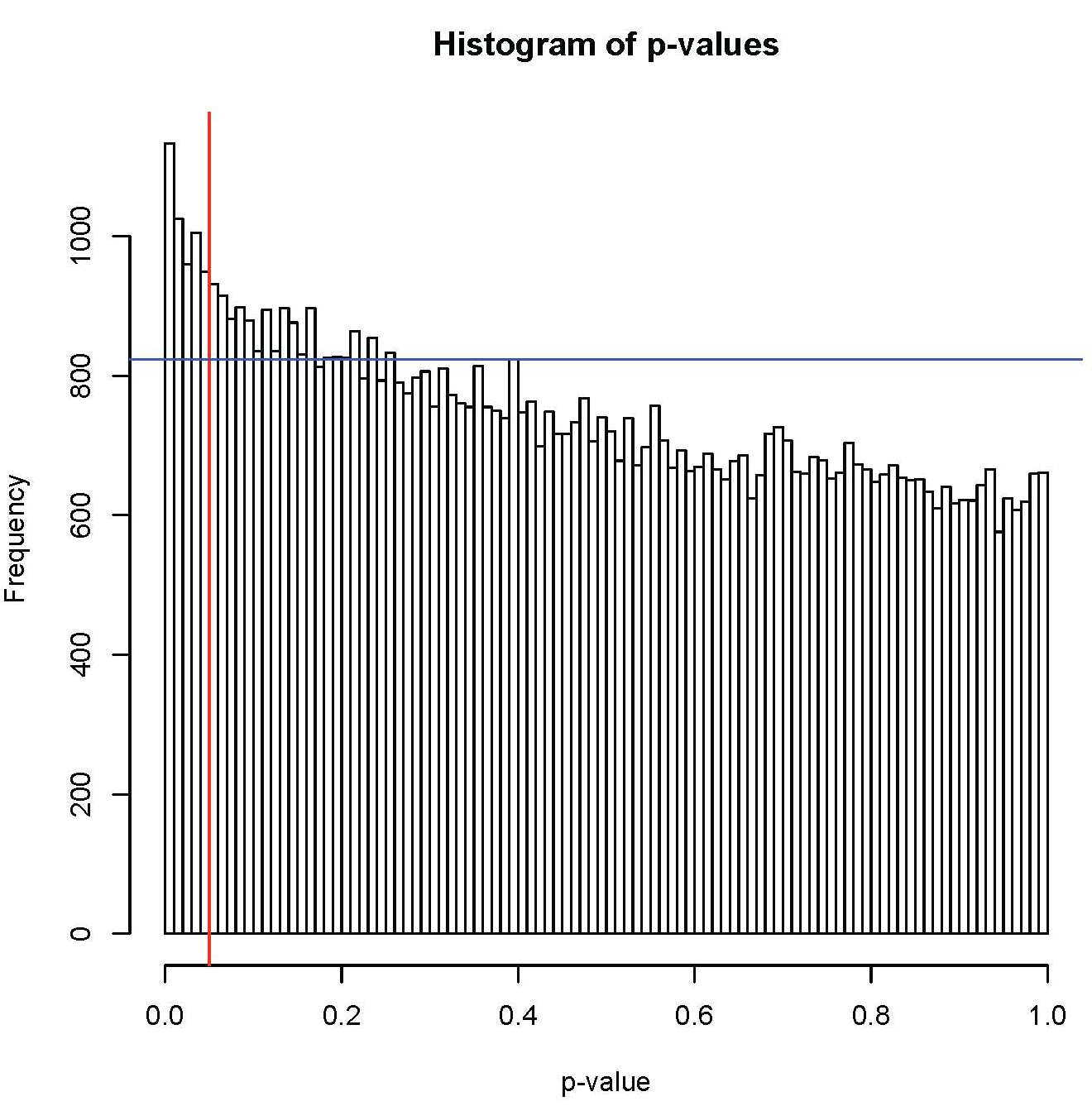

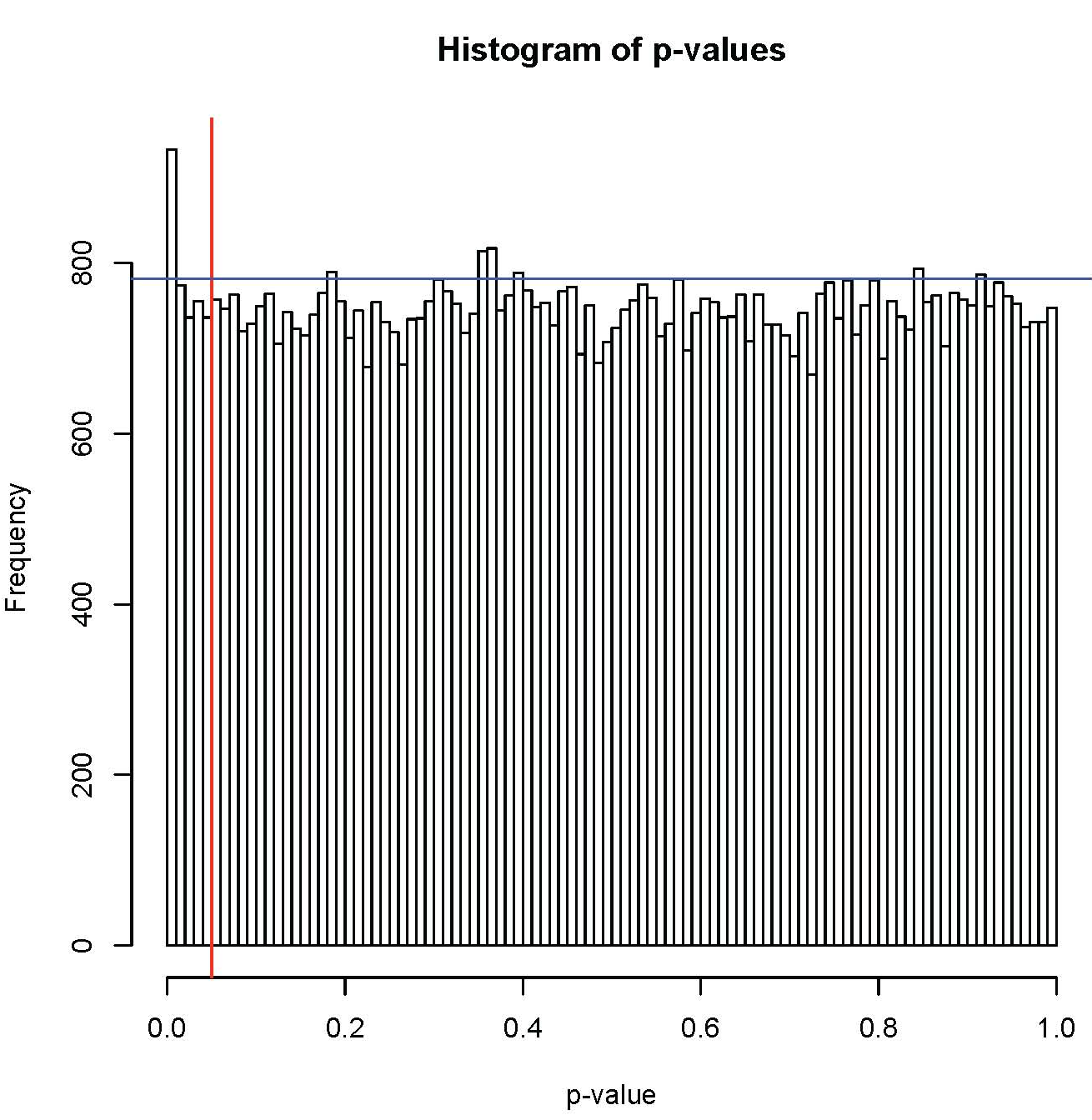


**LINES**


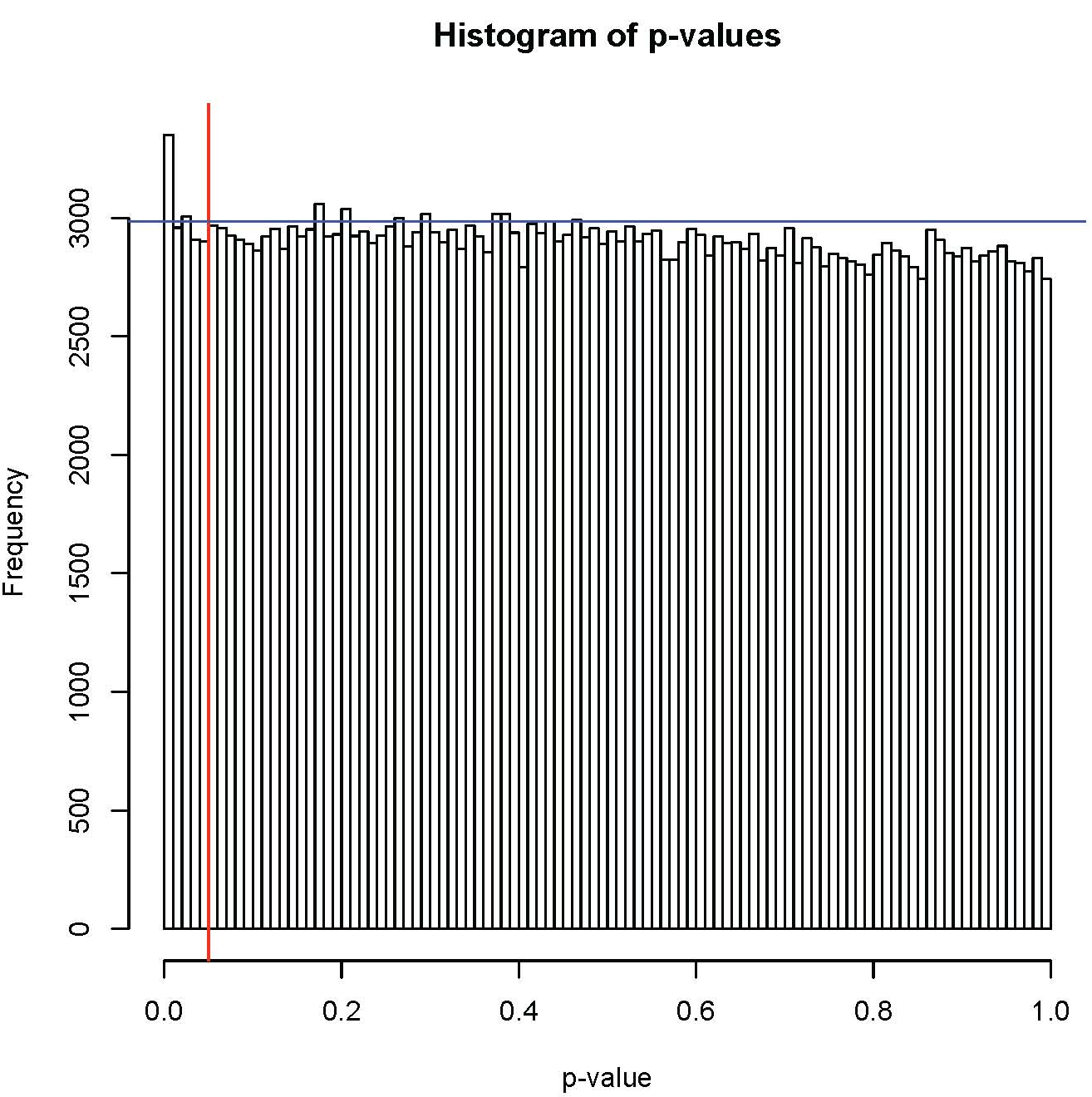

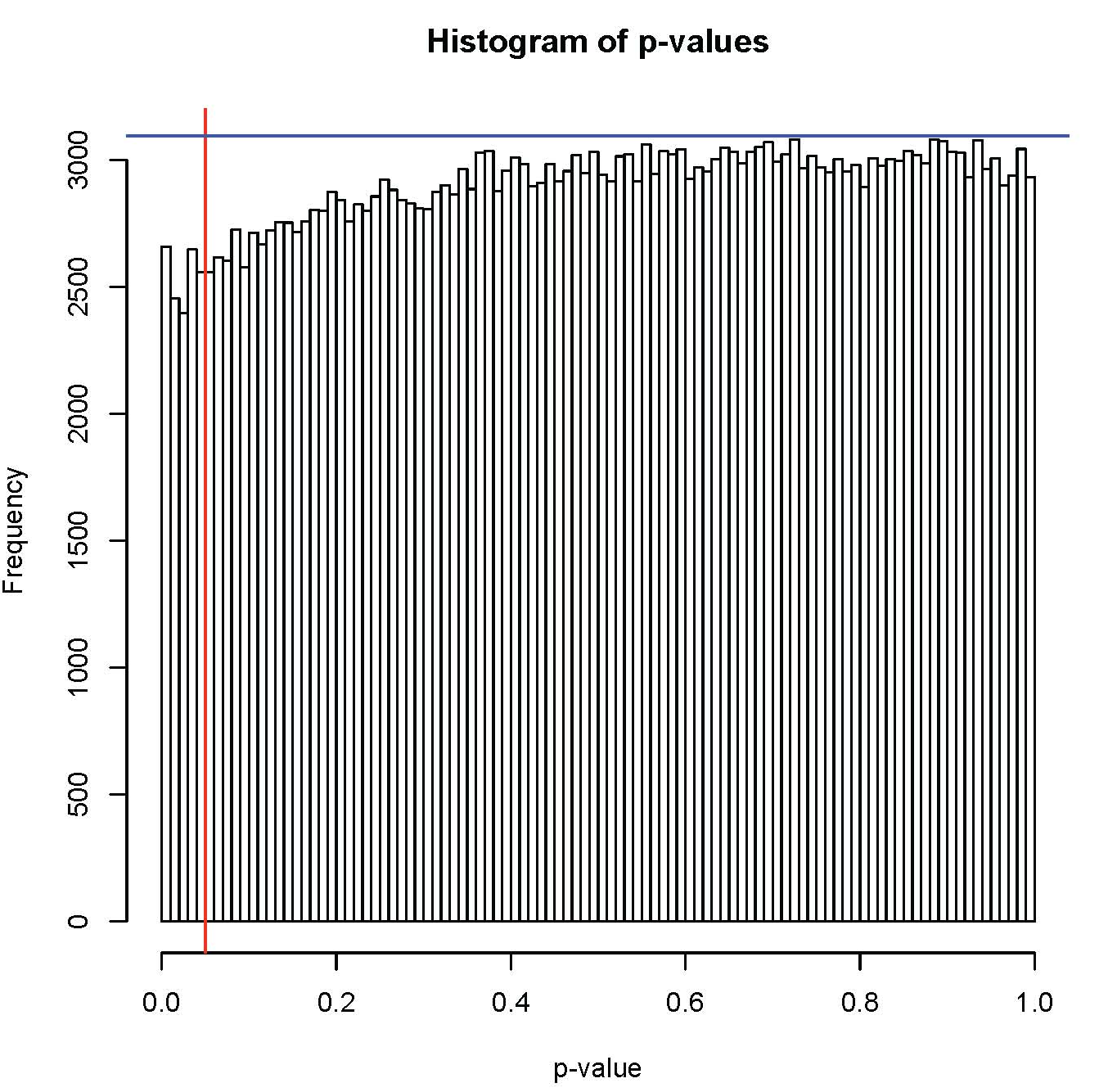

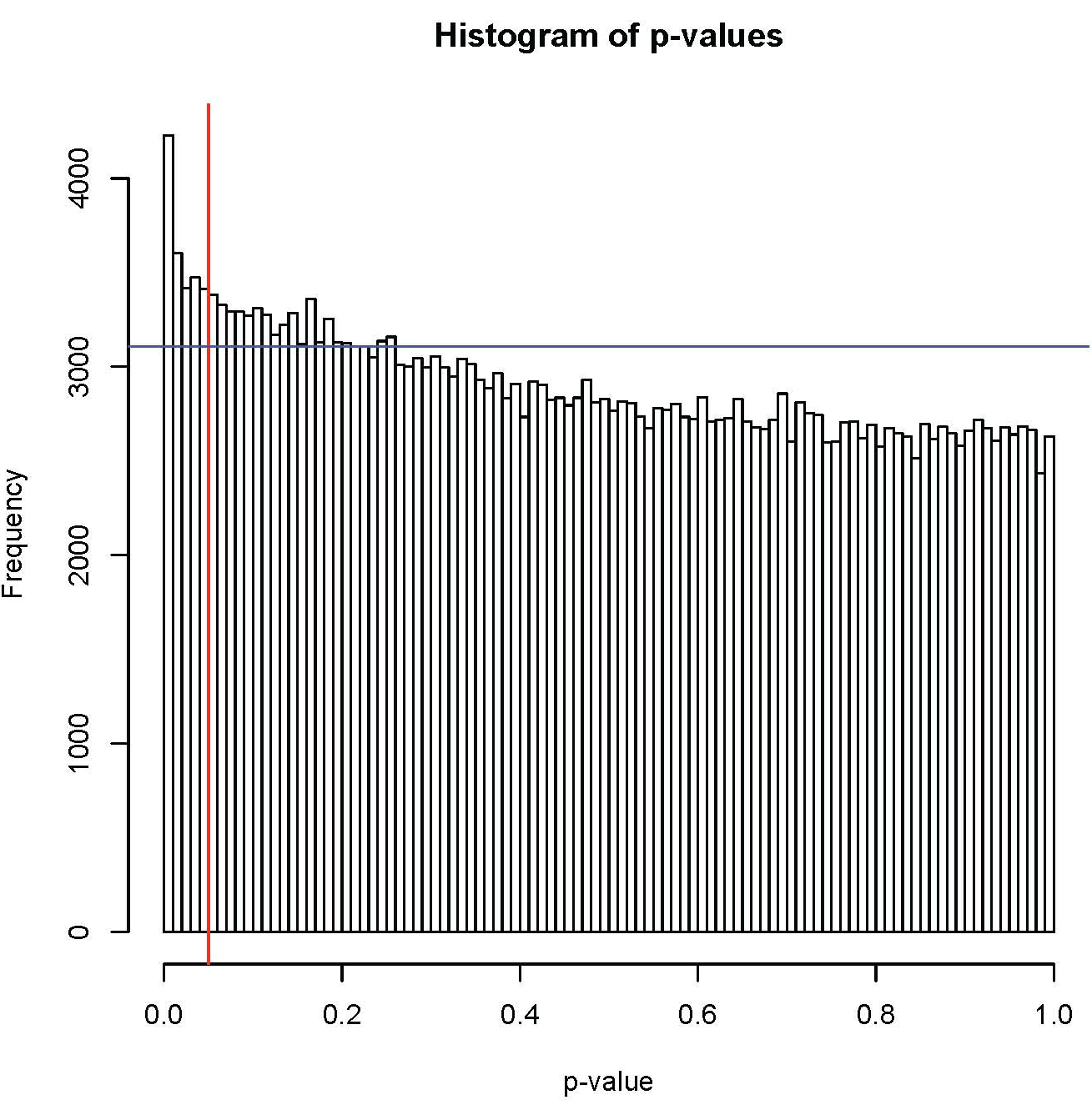

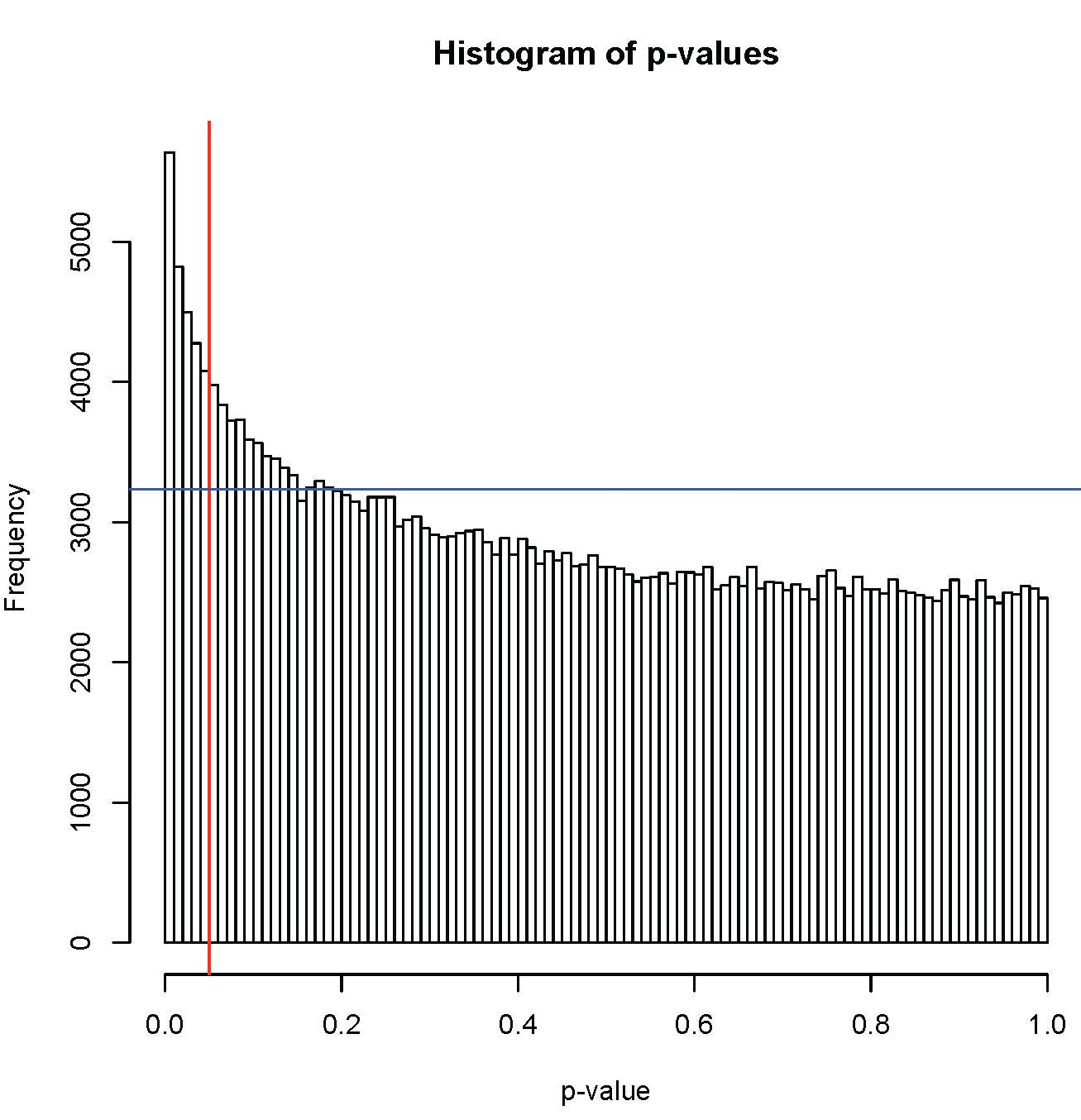

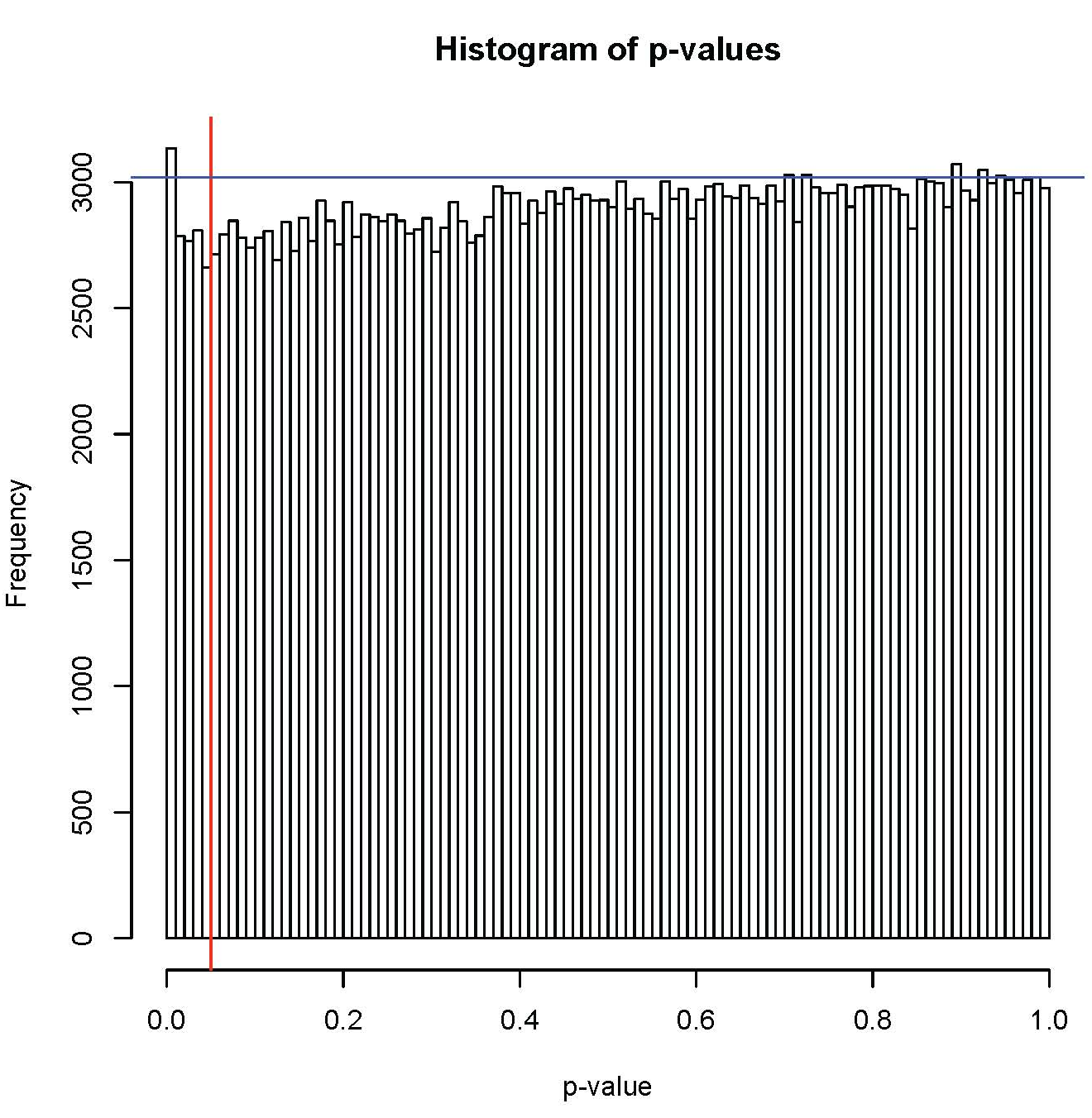


**LTRs**


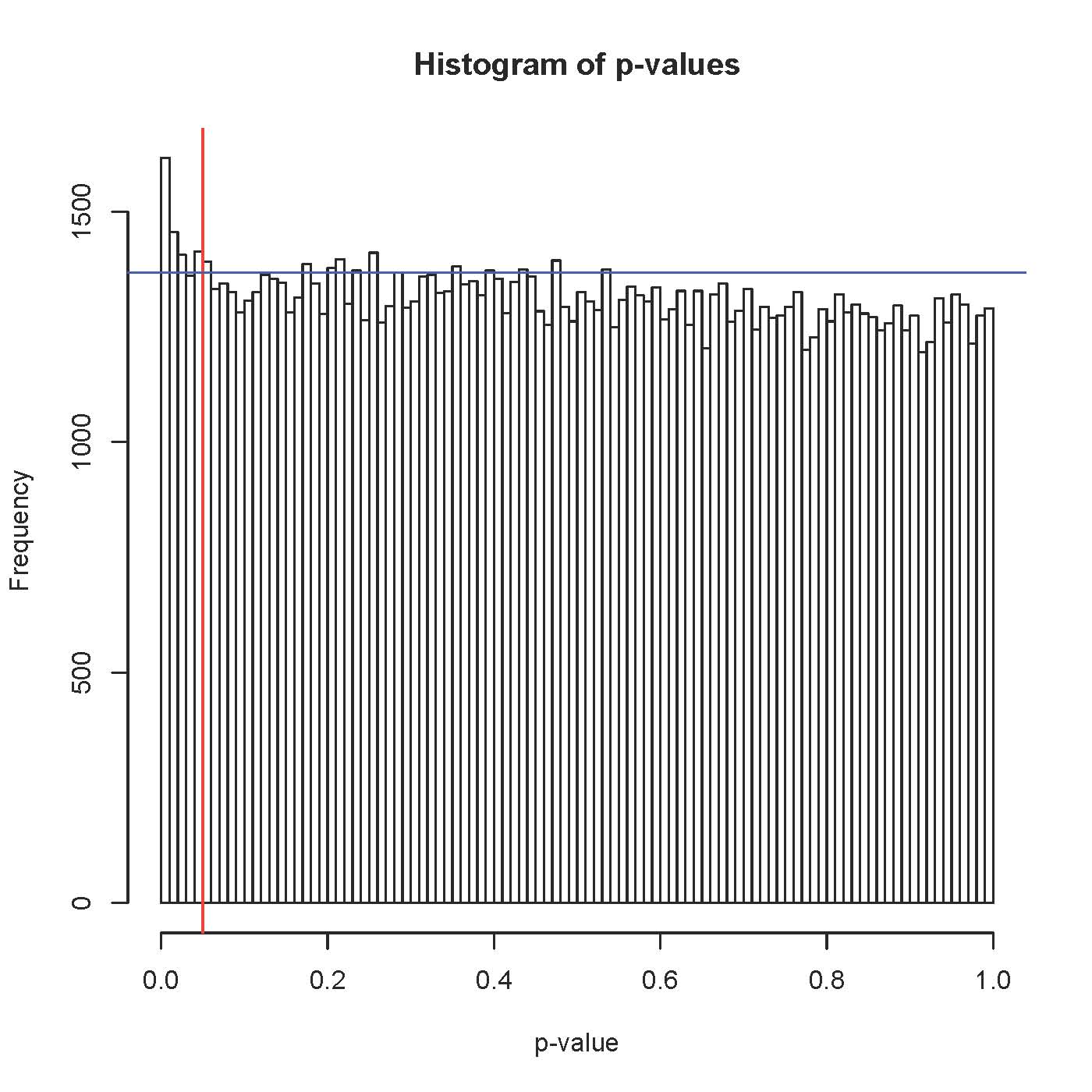

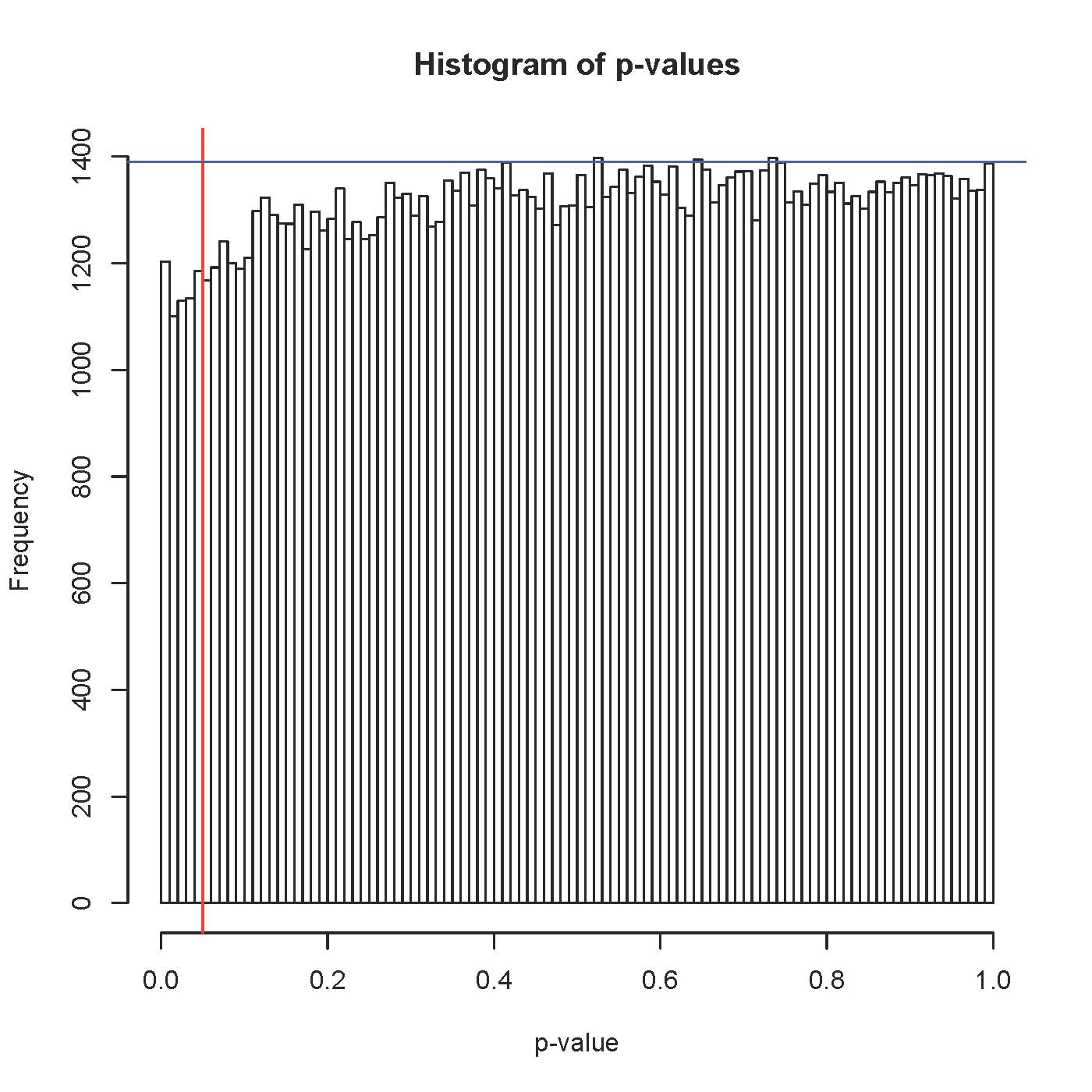

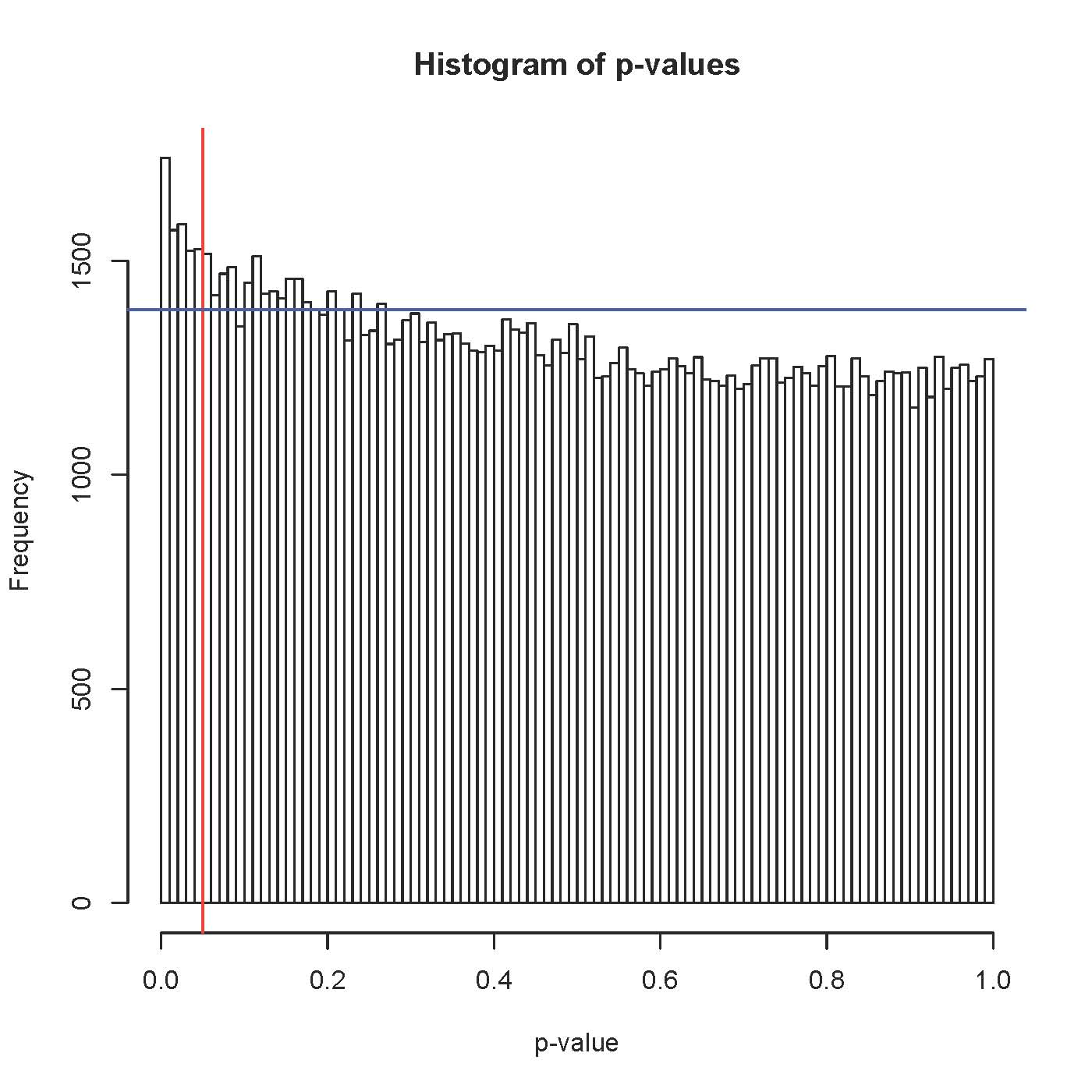

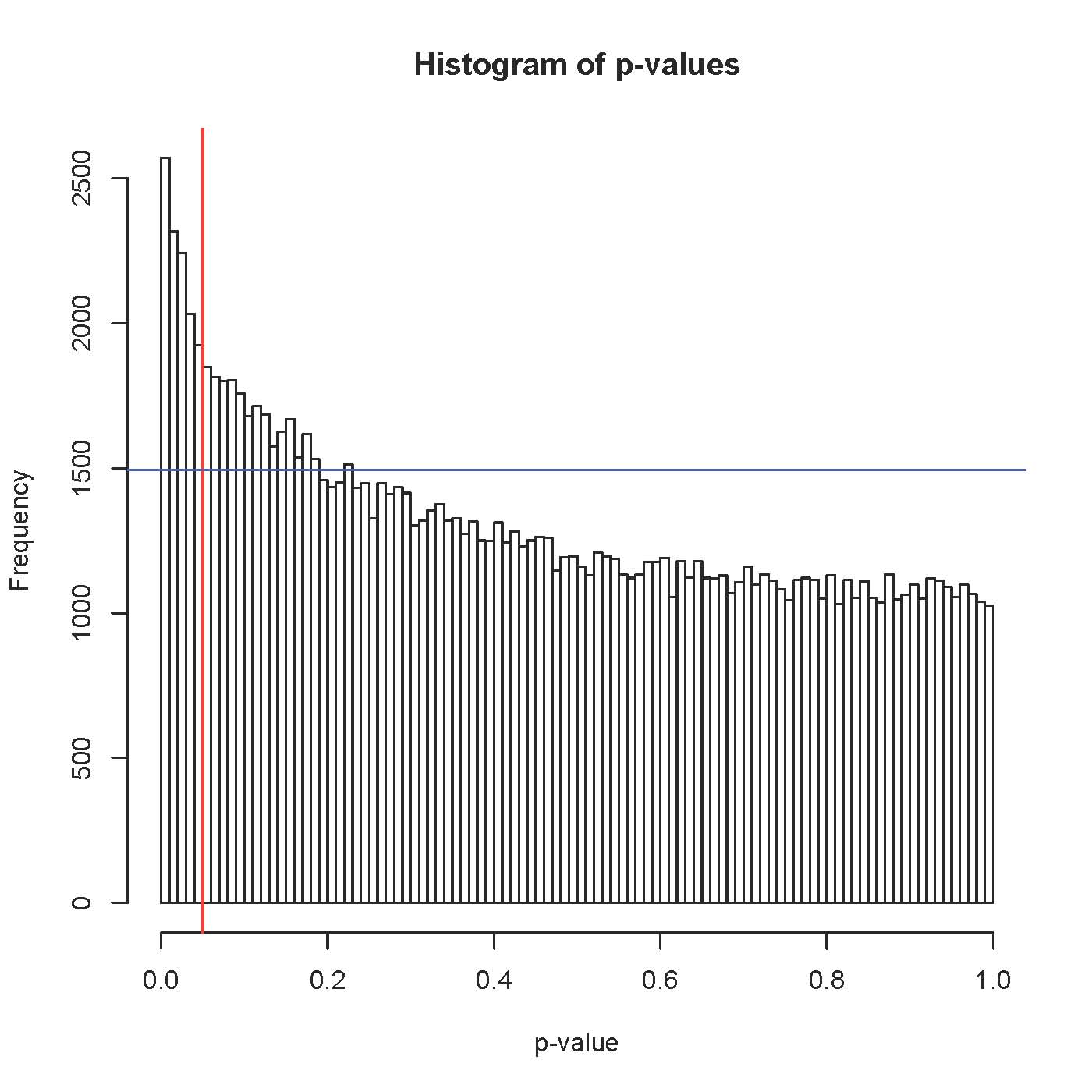

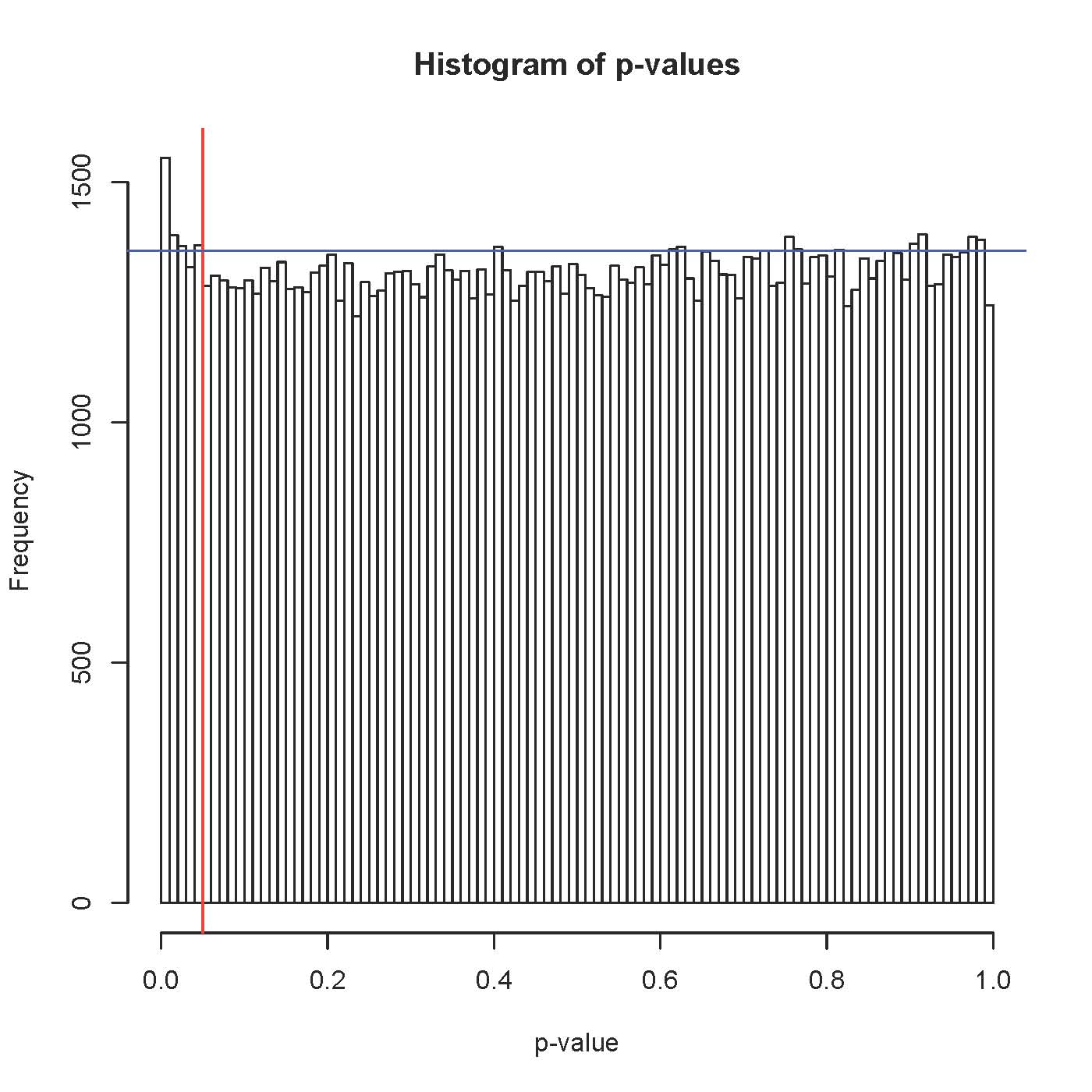


**t(8;21) versus t(15;17) versus NK versus Trisomy 8 versus All AMLs**

**all groups all groups all groups all groups versus all NBMs**
