# Supplementary material for: Genome Wide Analysis of Acute Myeloid Leukemia Reveal Leukemia Specific Methylome and Subtype Specific Hypomethylation of Repeats
Source: PLoS One. 2012 Mar 29;7(3):e33213. doi: 10.1371/journal.pone.0033213 (PMC3315563; doi:10.1371/journal.pone.0033213)
Supplement: Figure S13 — Histogram illustrating the distribution of uncorrected P values after testing equality of methylation between normal and leukemic samples for all genomic features and repeats. For random data the distribution is expected to be uniformly distributed across the unit interval (blue horizontal line). The frequency of P values<0.05 (red line) is higher than expected with particular enrichment of P values<0.001. (DOC) [file pone.0033213.s014.doc]

**Figure S13. Histogram illustrating the distribution of uncorrected P values after testing equality of methylation between normal and leukemic samples for all genomic features and repeats.** For random data the distribution is expected to be uniformly distributed across the unit interval (blue horizontal line). The frequency of P values < 0.05 (red line) is higher than expected with particular enrichment of P values < 0.001.

**
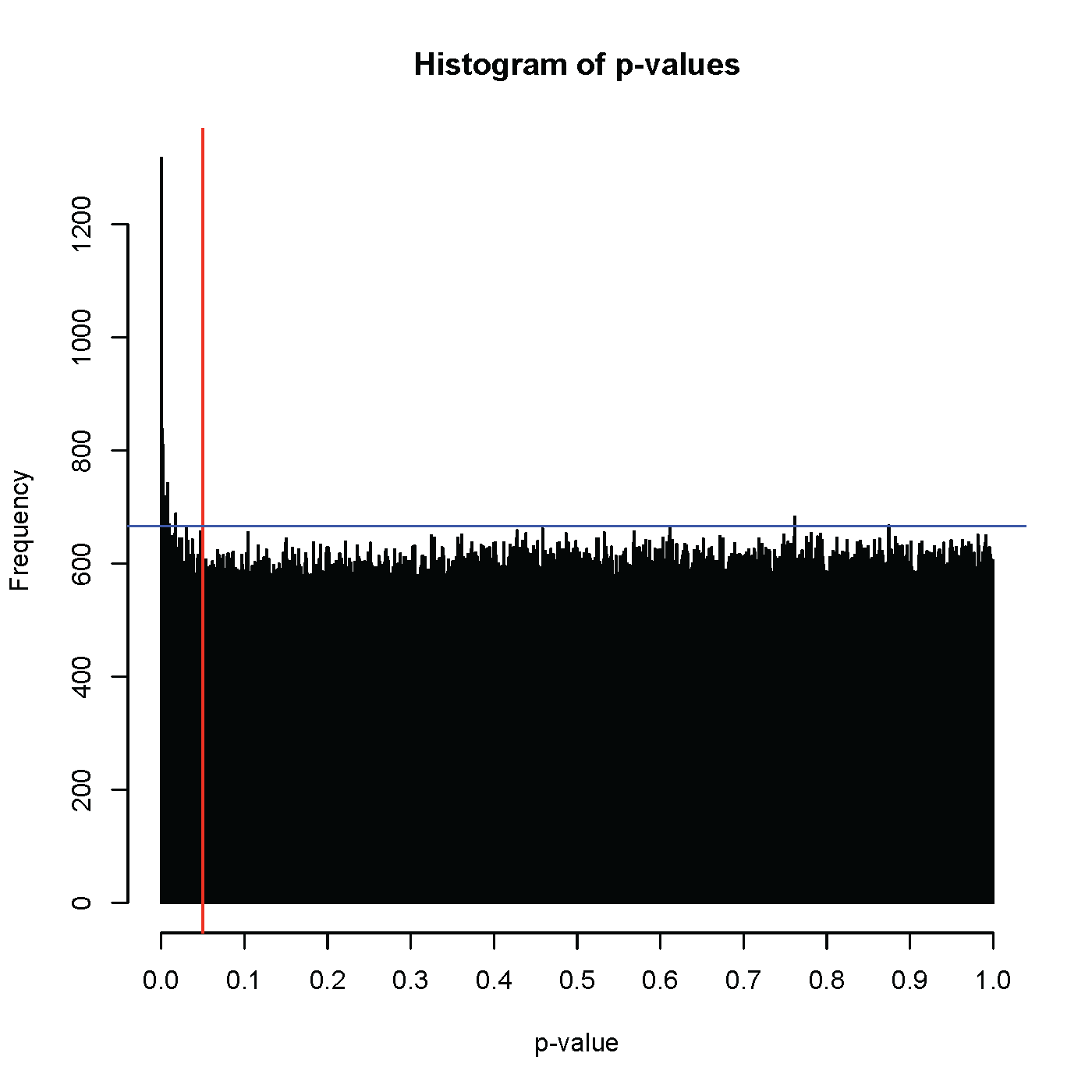
**
