# Supplementary material for: Genome Wide Analysis of Acute Myeloid Leukemia Reveal Leukemia Specific Methylome and Subtype Specific Hypomethylation of Repeats
Source: PLoS One. 2012 Mar 29;7(3):e33213. doi: 10.1371/journal.pone.0033213 (PMC3315563; doi:10.1371/journal.pone.0033213)
Supplement: Table S1 — Patients and control samples. (DOC) [file pone.0033213.s015.doc]

**Table S1. Patients and control samples.**

| **Study No.** | **Risk group** | **Sex** | **Age at presentation** | **FAB type** | **Blast %** | **Survival (years)** |
| --- | --- | --- | --- | --- | --- | --- |
| 1 | t(15;17) | M | 36 | M3 | 85 | 5.8 |
| 2 | t(15;17) | F | 69 | M3 | 80 | 15.8 |
| 3 | t(15;17) | M | 30 | M3 | 85 | 10.0 |
| 4 | NK | F | 64 | M2 | 60 | 0.82 |
| 5 | NK | M | 18 | M2 | 87 | 6.4 |
| 6 | NK | F | 56 | M1 | 71 | 0.53 |
| 7 | t(8;21) | F | 27 | M2 | 95 | 13.1 |
| 8 | t(8;21) | M | 34 | M2 | 88 | 9.2 |
| 9 | t(8;21) | M | 67 | M2 | 70 | 0.16 |
| 10 | +8 | M | 46 | M5 | 45 | 2.1 |
| 11 | +8 | M | 21 | M5A | 82 | 0.65 |
| 12 | +8 | M | 43 | NA | 73 | 0.51 |
| 13 | NBM | M | 23 | NA | < 5 | NA |
| 14 | NBM | M | 28 | NA | < 5 | NA |
| 15 | NBM | M | 22 | NA | < 5 | NA |
| 16 | NBM | F | 54 | NA | < 5 | NA |

M; male, F; female, PB; NA; not applicable.
