# Supplementary material for: Genome Wide Analysis of Acute Myeloid Leukemia Reveal Leukemia Specific Methylome and Subtype Specific Hypomethylation of Repeats
Source: PLoS One. 2012 Mar 29;7(3):e33213. doi: 10.1371/journal.pone.0033213 (PMC3315563; doi:10.1371/journal.pone.0033213)
Supplement: Table S2 — Criteria of reads generated from Illumina GAII, (DOC) [file pone.0033213.s016.doc]

**Table S2. Criteria of reads generated from Illumina GAII.**

| **Study No.** | **No. of total reads** | ***No. of uniquely mapped reads** | **% CpG Coverage** | **Pearson r True saturation** | **Pearson r**  **Estimated saturation** |
| --- | --- | --- | --- | --- | --- |
| 1 | 66 X 10 ^6^ | 26 X 10 ^6^ | 82 | 0.87 | 0.93 |
| 2 | 36 X 10 ^6^ | 21 X 10 ^6^ | 76 | 0.99 | 1 |
| 3 | 40 X 10 ^6^ | 24 X 10 ^6^ | 74 | 0.99 | 0.99 |
| 4 | 30 X 10 ^6^ | 12 X 10 ^6^ | 69 | 0.78 | 0.89 |
| 5 | 38 X 10 ^6^ | 22 X 10 ^6^ | 77 | 0.99 | 1 |
| 6 | 34 X 10 ^6^ | 20 X 10 ^6^ | 74 | 0.99 | 0.99 |
| 7 | 32 X 10 ^6^ | 13 X 10 ^6^ | 63 | 0.86 | 0.93 |
| 8 | 68 X 10 ^6^ | 24 X 10 ^6^ | 80 | 0.85 | 0.92 |
| 9 | 40 X 10 ^6^ | 23 X 10 ^6^ | 76 | 0.99 | 1 |
| 10 | 58 X 10 ^6^ | 24 X 10 ^6^ | 77 | 0.99 | 1 |
| 11 | 66 X 10 ^6^ | 38 X 10 ^6^ | 85 | 1 | 1 |
| 12 | 68 X 10 ^6^ | 41 X 10 ^6^ | 87 | 1 | 1 |
| 13 | 66 X 10 ^6^ | 29 X 10 ^6^ | 84 | 0.88 | 0.94 |
| 14 | 40 X 10 ^6^ | 24 X 10 ^6^ | 77 | 0.99 | 1 |
| 15 | 40 X 10 ^6^ | 24 X 10 ^6^ | 67 | 0.99 | 0.99 |
| 16 | 44 X 10 ^6^ | 26 X 10 ^6^ | 71 | 1 | 1 |

The uniquely mapped reads are reads that have unique chromosomal location with start and end sites. Pipeline software, which was used for filtering raw clusters (reads) generated from Illumina GAII, was upgraded from 1.4 (applied to studies No. 1- 9 & studies 13-16) to 1.6 (applied to studies No. 10-12).
