# Supplementary material for: Genome Wide Analysis of Acute Myeloid Leukemia Reveal Leukemia Specific Methylome and Subtype Specific Hypomethylation of Repeats
Source: PLoS One. 2012 Mar 29;7(3):e33213. doi: 10.1371/journal.pone.0033213 (PMC3315563; doi:10.1371/journal.pone.0033213)
Supplement: Table S3 — Description of the genomic regions from MeDIP-seq results. (DOC) [file pone.0033213.s017.doc]

**Table S3. Description of the genomic regions from MeDIP-seq results.**

| **Genomic feature** | **Description** | **^a^CpG density of aligned genomic feature** | **No. of regions detected by MeDIP-seq** |
| --- | --- | --- | --- |
| **Promoter** | ^b^TSS ± 1 Kb | **^d^**Bimodal (1.5%, 7.2%) | 19,571 |
| **Gene body** | All available exons and introns from 2^nd^ exon onward | 1.9% | 19,412 |
| **^c^CGI** | GC > 50%,  CpG _obs/exp_ ratio > 0.6 in a sequence length ≥ 200bp | 19.4% (CGIs inside/overlapped the promoters), 18.2% (CGIs ≥ 1 Kb outside the promoters). | 23,318 |
| **CGI shore** | 2 Kb from either side of a CGI | 2.3% upstream/ downstream. | 26,978 upstream shores, 26,977 downstream shores |

^a^CpG density % equals the observed number of CpGs in a specific sequence / the length of this sequence.

^b^TSS transcriptional start site.

^c^CGIs (CpG islands). **^d^**Bimodal CpG density; CpG density of promoters without CGIs ~ 1.5% , CpG density of promoters associated with CGIs ~ 7.2%.

All the genome annotation was based on (NCBI 36/hg18).

The methylation for each genomic region equals the average of all Batman scores arising from this genomic region.
