# Supplementary material for: Genome Wide Analysis of Acute Myeloid Leukemia Reveal Leukemia Specific Methylome and Subtype Specific Hypomethylation of Repeats
Source: PLoS One. 2012 Mar 29;7(3):e33213. doi: 10.1371/journal.pone.0033213 (PMC3315563; doi:10.1371/journal.pone.0033213)
Supplement: Table S4 — DMRs identified in AML versus NBM in 4 genomic regions. (DOC) [file pone.0033213.s018.doc]

**Table S4. DMRs identified in AML versus NBM in 4 genomic regions.**

| **Genomic feature** | **^a^DMRs between AML and NBM** | **Hypermethylated DMRs in AML** | **Hypomethylated DMRs in AML** |
| --- | --- | --- | --- |
| **Promoter** | 105 | 102 | 3 |
| **Gene body** | 72 | 57 | 15 |
| **CGI** | 704 | 470 | 234 |
| **CGI shore** | 310 | 258 | 52 |

^a^DMR differentially methylated regions.
