# Supplementary material for: Genome Wide Analysis of Acute Myeloid Leukemia Reveal Leukemia Specific Methylome and Subtype Specific Hypomethylation of Repeats
Source: PLoS One. 2012 Mar 29;7(3):e33213. doi: 10.1371/journal.pone.0033213 (PMC3315563; doi:10.1371/journal.pone.0033213)
Supplement: Table S5 — DMRs identified in AML subtypes in 4 genomic regions. (DOC) [file pone.0033213.s019.doc]

**Table S5 DMRs identified in AML subtypes in 4 genomic regions.**

**a.**

| **Genomic feature** | **t(8;21)**  **hypermethylated DMRs** | **t(8;21)**  **hypomethylated DMRs** | **t(15;17)**  **hypermethylated DMRs** | **t(15;17)**  **hypomethylated DMRs** |
| --- | --- | --- | --- | --- |
| **Promoters** | 191 | 32 | 29 | 4 |
| **Gene bodies** | 57 | 44 | 30 | 29 |
| **CGIs** | 710 | 358 | 255 | 118 |
| **CGI shores** | 331 | 314 | 88 | 70 |

**b.**

| **Genomic feature** | **NK hypermethylated DMRs** | **NK hypomethylated DMRs** | **Trisomy 8 hypermethylated DMRs** | **Trisomy 8 hypomethylated DMRs** |
| --- | --- | --- | --- | --- |
| **Promoters** | 54 | 11 | 12 | 61 |
| **Gene bodies** | 35 | 35 | 15 | 44 |
| **CGIs** | 240 | 174 | 177 | 151 |
| **CGI shores** | 109 | 60 | 31 | 110 |
