# Supplementary material for: Genome Wide Analysis of Acute Myeloid Leukemia Reveal Leukemia Specific Methylome and Subtype Specific Hypomethylation of Repeats
Source: PLoS One. 2012 Mar 29;7(3):e33213. doi: 10.1371/journal.pone.0033213 (PMC3315563; doi:10.1371/journal.pone.0033213)
Supplement: Table S6 — DMRs associated with repeats. (a, b) AML versus NBM, (c) between AML subtypes. (DOC) [file pone.0033213.s020.doc]

**Table S6 DMRs associated with repeats**. (a, b) AML versus NBM, (c) between AML subtypes.

**a.**

**b.**

| **Repeats** | **t(8;21)**  **hypermethylated DMRs** | **t(8;21)**  **hypomethylated DMRs** | **t(15;17)**  **hypermethylated DMRs** | **t(15;17)**  **hypomethylated DMRs** |
| --- | --- | --- | --- | --- |
| **Satellites** | - | - | - | 1 |
| **SINEs** | 421 | 764 | 273 | 567 |
| **LTRs** | 228 | 631 | 128 | 485 |
| **LINEs** | 445 | 902 | 231 | 757 |

**c.**

| **Repeats** | **NK hypermethylated DMRs** | **NK hypomethylated DMRs** | **Trisomy 8 hypermethylated DMRs** | **Trisomy 8 hypomethylated DMRs** |
| --- | --- | --- | --- | --- |
| **Satellites** | - | 3 | 9 | 4 |
| **SINEs** | 516 | 730 | 436 | 949 |
| **LTRs** | 366 | 567 | 399 | 612 |
| **LINEs** | 615 | 1008 | 626 | 1004 |

| **Repeats** | **No. of aligned repeats** | **DMRs between AML and NBM** | **Hypermethylated DMRs in AML** | **Hypomethylated DMRs in AML** |
| --- | --- | --- | --- | --- |
| **Satellites** | 1,213, 40% are centromeric | 1 | 0 | 1 |
| **SINEs** | 75,793, 53% are Alu repeats | 721 | 444 | 277 |
| **LTRs** | 132,581 | 455 | 325 | 130 |
| **LINEs** | 292,703 | 713 | 488 | 225 |
