# Supplementary material for: Genome Wide Analysis of Acute Myeloid Leukemia Reveal Leukemia Specific Methylome and Subtype Specific Hypomethylation of Repeats
Source: PLoS One. 2012 Mar 29;7(3):e33213. doi: 10.1371/journal.pone.0033213 (PMC3315563; doi:10.1371/journal.pone.0033213)
Supplement: Table S7 — Direct bisulfite sequencing validation of selected genomic regions. (DOC) [file pone.0033213.s021.doc]

**Table S7. Direct bisulfite sequencing validation of selected genomic regions.**

| ***Genomic regions** | **Location** | **No. of CGs** | **MeDIP-seq absolute methylation difference** | **P value (MeDIP versus direct bisulfite)** | **R^2^ (Pearson’s)** |
| --- | --- | --- | --- | --- | --- |
| ***DPP6* (Promoter)** | Chr7:153214701-153215064 | 36 | 0.45601 | 0.0005 | 0.84 |
| ***SPHKAP***  **(Promoter)** | Chr2:228754201-228754550 | 23 | 0.44987 | 0.02 | 0.5 |
| **CGIs (Present in gene body of LAMA5 gene)** | Chr20:60319301-60319515 | 14 | 0.62537 | 0.008 | 0.72 |
| **CGI shore (sequence a) (Intergenic CGI shore)** | Chr7:8448450-8448726 | 18 | 0.30308 | 0.002 | 0.82 |
| **CGI shore (sequence b) (Intergenic CGI shore)** | Chr7:8448727-8448894 | 13 | 0.30308 | <0.0001 | 0.96 |

*****The genomic regions were chosen from differentially methylated genes/genomic regions in AML versus NBM except CGI, which was differentially methylated in NBM versus AML.
