# Supplementary material for: Genome Wide Analysis of Acute Myeloid Leukemia Reveal Leukemia Specific Methylome and Subtype Specific Hypomethylation of Repeats
Source: PLoS One. 2012 Mar 29;7(3):e33213. doi: 10.1371/journal.pone.0033213 (PMC3315563; doi:10.1371/journal.pone.0033213)
Supplement: Table S8 — Pyrosequencing validation of selected genomic regions. (a) AML versus NBM, (b) in AML subtypes. (DOC) [file pone.0033213.s022.doc]

**Table S8. Pyrosequencing validation of selected genomic regions.**

(a) AML versus NBM, (b) in AML subtypes.

| **Gene** | **MeDIP-seq** | **AML patients** | **AML cell lines** | **NBMs** |
| --- | --- | --- | --- | --- |
| ***SPHKAP* chr2: 228753601-228753665** | AML > NBM (**promoter**) | *53% | 82% | 17% |
| ***DPP6* chr7: 153214816-153214843** | AML > NBM (**promoter**) | 23% | 61% | 3% |
| ***ST6GAL2* chr2: 106869095-106869136** | t(8;21) > AML & NBM (**promoter**) | t(8;21) mean= 43%  Other AMLs mean = 12% | 68% | 1% |
| ***HHEX* chr10: 94442251-94442377** | Trisomy 8 > AML & NBM (**CGI** **within gene body**) | Trisomy 8 mean=42%  Other AMLs mean = 14% | 50% | 6% |
| **AluJb chr7: 148474346-148474580** | t(8;21) < AML & NBM (**within gene body of ZNF398)** | t(8;21) mean = 7%, other AMLs mean = 23% | 12% | 29% |

*Percentage= average of methylation of all CpG sites sequenced as calculated by pyrosequencing.

For pyrosequencing validation we selected 2 genes that showed significant differential methylation between AML versus NBM: *SPHKAP* (promoter; 9 CpG sites) and *DPP6* (promoter; 4 CpG sites). Additionally, we chose 2 genes that showed differential methylation in a specific AML subtype; *ST6GAL2* in t(8;21) AML (promoter; 8 CpG sites) and *HHEX* in trisomy 8 AML (CGI; 9 CpG sites). To confirm the hypomethylated distinctive repeats associated with AML subtypes, we also selected an Alu repeat that was hypomethylated in t(8;21) versus the other AML subtypes and NBMs (3 CpG sites).

b.

| **Gene** | **t(8;21)** | **t(15;17)** | **Inv 16** | **+8** | **+21** | **t(9;22)** | **t(6;9)** | **t(v;11q23)** | **t(9;11)** | **Complex** | **NKs** | **AML cell lines** | **N** |
| --- | --- | --- | --- | --- | --- | --- | --- | --- | --- | --- | --- | --- | --- |
| ***SPHKAP*** | *65/6 | 63/10 | 63/3 | 45/5 | 49/3 | 42/1 | 50/1 | 48/2 | 20/2 | 60/5 | 50/24 | 82/8 | 17/5 |
| ***DPP6*** | 41/6 | 27/10 | 15/3 | 19/5 | 9/3 | 3/1 | 32/1 | 20/2 | 6/1 | 23/5 | 22/20 | 61/6 | 3/4 |
| ***ST6GAL2*** | 43/6 | 20/9 | 15/3 | 17/5 | 8/3 | 2/1 | 11/1 | 12/2 | 7/2 | 4/5 | 9/22 | 68/5 | 1/4 |
| ***HHEX*** | 3/5 | 22/4 | 3/6 | 50/10 | 0 | 0 | 0 | 0 | 0 | 35/5 | 8/30 | 50/8 | 6/5 |
| **Alu Repeat** | 7/7 | 25/4 | 12/6 | 15/10 | 0 | 0 | 0 | 0 | 0 | 24/5 | 15/30 | 12/8 | 29/5 |

*65/6; 65 is the average % of all CpG sites methylation that passed the statistic tests as calculated by pyrosequencing, 6 is the number of patients tested in this AML subgroup. N; NBMs.
