# Supplementary material for: Genome Wide Analysis of Acute Myeloid Leukemia Reveal Leukemia Specific Methylome and Subtype Specific Hypomethylation of Repeats
Source: PLoS One. 2012 Mar 29;7(3):e33213. doi: 10.1371/journal.pone.0033213 (PMC3315563; doi:10.1371/journal.pone.0033213)
Supplement: Table S9 — DNA methylation of over- and under expressed genes in t(8;21), t(15;17) & NK AML subgroups that were included in MeDIP-seq experiment. (DOC) [file pone.0033213.s023.doc]

**Table S9. DNA methylation of over- and under expressed genes in t(8;21), t(15;17) & NK AML subgroups that were included in MeDIP-seq experiment.**

| **Genes ID** | **Promoter location** | **Batman methylation scores** | | |
| --- | --- | --- | --- | --- |
|  |  | Patient 1 | Patient 2 | Patient 3 |
| **t(8;21) AML over expressed genes** |  | t(8;21)-patient 1 | t(8;21)-patient 2 | t(8;21)-patient 3 |
| TRH | Chr3:131175254:131177253 | 0.2388 | 0.30795 | 0.35145 |
| POU4F1 | Chr13:78074696:78076695 | 0.2488 | 0.17785 | 0.13375 |
| NEUROD1 | Chr 2:182252626:182254625 | 0.68575 | 0.3379 | 0.4336 |
| PRAME | Chr22:21230696:21232695 | 0.38615 | 0.5156 | 0.41565 |
| PGDS | Chr4:95482050:95484049 | 0.56635 | 0.6688 | 0.4108 |
| RGS10 | Chr10:121291212:121293211 | 0.0901 | 0.08475 | 0.06675 |
| GPM6B | Chr23:13865758:13867757 | 0.158 | 0.1776 | 0.2579 |
| **t(8;21) AML under expressed genes** |  |  |  |  |
| HOXA9 | Chr7:27174794:27176793 | 0.4054 | 0.4528 | 0.51105 |
| HOXA4 | Chr7:27135877-2713783653 | 0.227 | 0.6091 | 0.6251 |
| SPINK2 | Chr4:57381654:57383653 | 0.5383 | 0.43805 | 0.513 |
| CAPG | Chr2:85490187:85492186 | 0.4027 | 0.7297 | 0.6955 |
| **t(15;17) AML over expressed genes** |  | T(15;17) patient 1 | T(15;17) patient 2 | T(15;17) patient 3 |
| CPA3 | Chr3:150064772:150066771 | 0.76785 | 0.57075 | 0.58205 |
| SLPI | Chr20:43315620:43317619 | 0.83215 | 0.82165 | 0.8381 |
| FGF13 | Chr23:137893912:137895911 | 0.78655 | 0.69755 | 0.8783 |
| DLX2 | Chr2:172674724:172676723 | 0.14465 | 0.12035 | 0.18775 |
| CST7 | Chr20:24876867:24878866 | 0.56995 | 0.2691 | 0.4761 |
| RUNX3 | Chr1:25163088:25165087 | 0.57155 | 0.6533 | 0.73955 |
| FGFR1 | Chr8:38444509:38446508 | 0.3452 | 0.4579 | 0.47255 |
| PPARG | Chr3:12303071:12305070 | 0.2521 | 0.18545 | 0.31525 |
| **t(15;17) AML under expressed genes** |  |  |  |  |
| SLC7A7 | Chr14:22357840:22359839 | 0.7648 | 0.7801 | 0.8007 |
| HOXA9 | Chr7:27174794:27176793 | 0.3395 | 0.32935 | 0.4532 |
| ITPR1 | Chr3:4509035:4511034 | 0.17325 | 0.1999 | 0.15125 |
| MEF2C | Chr5:88213780:88215779 | 0.22205 | 0.21665 | 0.23685 |
| HOXA4 | Chr7:27135870:27137876 | 0.7661 | 0.5757 | 0.8948 |
| ARHGAP4 | Chr23:152843908:152845907 | 0.41815 | 0.4004 | 0.4464 |
| NCF2 | Chr1:181825634:181827633 | 0.7065 | 0.4899 | 0.725 |
| **NK AML over expressed genes** |  | NK patient 1 | NK patient 2 | NK patient 3 |
| HOXA9 | Chr7:27174794:27176793 | 0.30085 | 0.20595 | 0.19795 |
| HOXB2 | Chr17:43976392:43978391 | 0.2698 | 0.3119 | 0.2602 |
| HOXA4 | Chr7:27135870:27137876 | 0.35945 | 0.87205 | 0.7333 |
| PLXNC1 | Chr12:93065631:93067630 | 0.21075 | 0.1668 | 0.2004 |
| **NK AML under expressed genes** |  |  |  |  |
| MPO | Chr17:53712295:53714294 | 0.6347 | 0.83765 | 0.82945 |
| FGFR1 | Chr8:38444509:38446508 | 0.2855 | 0.3869 | 0.3457 |
