# Supplementary material for: Genome Wide Analysis of Acute Myeloid Leukemia Reveal Leukemia Specific Methylome and Subtype Specific Hypomethylation of Repeats
Source: PLoS One. 2012 Mar 29;7(3):e33213. doi: 10.1371/journal.pone.0033213 (PMC3315563; doi:10.1371/journal.pone.0033213)
Supplement: Table S10 — DNA methylation of over- and under expressed genes in trisomy 8 AML subgroup that was included in MeDIP-seq experiment. (DOC) [file pone.0033213.s024.doc]

**Table S10. DNA methylation of over- and under expressed genes in trisomy 8 AML subgroup that was included in MeDIP-seq experiment.**

| **Genes ID** | **Promoter location** | **Batman methylation scores** | | |
| --- | --- | --- | --- | --- |
|  |  | Patient 1 | Patient 2 | Patient 3 |
| **Trisomy 8 AML over expressed genes** |  | Trisomy 8-patient 1 | Trisomy 8-patient 2 | Trisomy 8-patient 3 |
| IGKV1D-8 | Chr 2: 89896080: 89898079 | 0.2683 | 0.4485 | 0.5362 |
| TAF2 | Chr 8: 120913255: 120915254 | 0.66775 | 0.5087 | 0.3461 |
| COPE | Chr 19: 18890199: 18892198 | 0.1918 | 0.1868 | 0.2176 |
| IGHA1 | Chr 14: 105245046: 105247045 | 0.49075 | 0.66435 | 0.4069 |
| NFIL3 | Chr 9: 93224965: 93226964 | 0.07985 | 0.09075 | 0.11435 |
| CCT8 | Chr 21: 29366989: 29368988 | 0.11265 | 0.14765 | 0.1582 |
| PSAP | Chr 10: 73280132: 73282131 | 0.24215 | 0.16925 | 0.4575 |
| IGBP1 | Chr 23: 69269044: 69271043 | 0.1912 | 0.2266 | 0.0922 |
| BTG1 | Chr 12: 91062751: 91064750 | 0.07145 | 0.0707 | 0.0611 |
| DBI | Chr 2: 119839975: 119841974 | 0.1754 | 0.3241 | 0.2013 |
| LYN | Chr 8: 56953949: 56955948 | 0.31375 | 0.21285 | 0.2048 |
| PTK2 | Chr 8: 142079514: 142081513 | 0.14975 | 0.1273 | 0.1273 |
| TCEB1 | Chr 8: 75045959: 75047958 | 0.1714 | 0.18275 | 0.15245 |
| FXR1 | Chr 3: 182112147: 182114146 | 0.23765 | 0.2642 | 0.2508 |
| **Trisomy 8 AML under expressed genes** |  | Trisomy 8-patient 1 | Trisomy 8-patient 2 | Trisomy 8-patient 3 |
| STAT1 | Chr 2: 191586181: 191588180 | 0.39205 | 0.35755 | 0.2842 |
| ELA1 | Chr 12: 50025730: 50027729 | 0.6701 | 0.78815 | 0.74805 |
| MPO | Chr 17: 53712295: 53714294 | 0.6427 | 0.7478 | 0.6086 |
| SLC7A5 | Chr 16: 86459615: 86461614 | 0.1012 | 0.06255 | 0.0547 |
| MCM5 | Chr 22: 34125117: 34127116 | 0.38965 | 0.3915 | 0.3254 |
| RALGDS | Chr 9: 135013542: 135015541 | 0.1313 | 0.13735 | 0.2413 |
| PRG2 | Chr 11: 56913688: 56915687 | 0.63585 | 0.8634 | 0.794 |
| ARAF | Chr 23: 47304523: 47306522 | 0.2604 | 0.21615 | 0.1918 |
| TPR | Chr 1: 184610383: 184612382 | 0.1768 | 0.1503 | 0.0684 |
| IL1B | Chr 2: 113309827: 113311826 | 0.4078 | 0.7727 | 0.7141 |
| MYC | Chr 8: 128815863: 128817862 | 0.1053 | 0.0887 | 0.20465 |
| FUS | Chr 16: 31097955: 31099954 | 0.10195 | 0.10305 | 0.07165 |
| APOC1 | Chr 19: 50108420: 50110419 | 0.6994 | 0.4572 | 0.23565 |
| NBL1 | Chr 1: 19841314: 19843313 | 0.4798 | 0.57355 | 0.548 |
| EPHB1 | Chr 3: 135995945: 135997944 | 0.4255 | 0.1898 | 0.20795 |
| CLC | Chr 19: 44920512: 44922511 | 0.7888 | 0.78925 | 0.6744 |
| MCM3 | Chr 6: 52256541: 52258540 | 0.1322 | 0.2834 | 0.2697 |
