# Supplementary material for: Genome Wide Analysis of Acute Myeloid Leukemia Reveal Leukemia Specific Methylome and Subtype Specific Hypomethylation of Repeats
Source: PLoS One. 2012 Mar 29;7(3):e33213. doi: 10.1371/journal.pone.0033213 (PMC3315563; doi:10.1371/journal.pone.0033213)
Supplement: Table S11 — The overlapped genes between the results of MeDIP-seq study and array-based study for t(15;17) AML. (DOC) [file pone.0033213.s025.doc]

**Table S11. The overlapped genes between the results of MeDIP-seq study and array-based study for t(15;17) AML.**

|  | **Gene ID** | **absolute methylation difference (MeDIP-seq)** | **P value (MeDIP-seq)** | **(HELP) REFSEQ** | **HELP status** |
| --- | --- | --- | --- | --- | --- |
| **t(15;17) AML gene body, P < 0.05** | CCDC96 | 0.166367539 | 0.008595658 | NM_153376 | Hypermethylated |
|  | EFNB3 | 0.11348914 | 0.002303019 | NM_001406 | Hypermethylated |
|  | CTDP1 | 0.070320972 | 0.006361778 | NM_048368 | Hypomethylated |
|  | PLD3 | 0.040474068 | 0.023572138 | NM_012268 | Hypomethylated |
|  | C21orf55 | 0.122434283 | 0.005500719 | NM_017833 | Hypomethylated |
| **t(15;17) AML promoters, P < 0.05** | CCDC96 | 0.102184606 | 0.011459895 | NM_153376 | Hypermethylated |
|  | CBLN4 | 0.203204604 | 0.00826159 | NM_080617 | Hypermethylated |
|  | TCP11 | 0.137933767 | 0.027689113 | NM_001093728 | Hypomethylated |
|  | CD3D | 0.11644395 | 0.016107599 | NM_000732 | Hypomethylated |
| **t(15;17) AML CGIs, P < 0.05** | TCP11 | 0.097680363 | 0.022919236 | NM_018679 | Hypomethylated |

Genes that have absolute methylation difference labeled red are hypermethylated in MeDIP-seq, genes have absolute methylation difference labeled green are hypomethylated in MeDIP-seq.
