# Supplementary material for: Genome Wide Analysis of Acute Myeloid Leukemia Reveal Leukemia Specific Methylome and Subtype Specific Hypomethylation of Repeats
Source: PLoS One. 2012 Mar 29;7(3):e33213. doi: 10.1371/journal.pone.0033213 (PMC3315563; doi:10.1371/journal.pone.0033213)
Supplement: Table S12 — The overlapped genes between the results of MeDIP-seq study and array-based study for t(8;21) AML. (DOC) [file pone.0033213.s026.doc]

**Table S12. The overlapped genes between the results of MeDIP-seq study and array-based study for t(8;21) AML.**

| **Cluster/cytogenetic subgroup** | **Gene ID** | **absolute methylation difference (MeDIP-seq)** | **P value (MeDIP-seq)** | **(HELP) REFSEQ** | **HELP status** |
| --- | --- | --- | --- | --- | --- |
| **t(8;21) AML gene body, P < 0.05** | YTHDF2 | 0.049138331 | 0.025445848 | NM_016258 | Hypermethylated |
|  | ZNF501 | 0.073802318 | 0.032618972 | NM_145044 | Hypermethylated |
|  | PCDHAC2 | 0.077956863 | 0.001810847 | NM_018902 | Hypermethylated |
|  | PCDHB16 | 0.157136955 | 0.006028352 | NM_020957 | Hypermethylated |
|  | PCDHB11 | 0.222558496 | 0.000521823 | NM_018931 | Hypermethylated |
|  | PCDHB14 | 0.204600434 | 0.004087562 | NM_018934 | Hypermethylated |
|  | PPARD | 0.042234943 | 0.012778396 | NM_006238 | Hypomethylated |
|  | C9orf79 | 0.120246549 | 0.019504062 | NM_178828 | Hypomethylated |
|  | C12orf10 | 0.072701608 | 0.011939341 | NM_021640 | Hypomethylated |
| **t(8;21) AML promoters, P < 0.05** | CCDC96 | 0.102184606 | 0.011459895 | NM_153376 | Hypermethylated |
|  | CBLN4 | 0.203204604 | 0.00826159 | NM_080617 | Hypermethylated |
|  | TCP11 | 0.137933767 | 0.027689113 | NM_001093728 | Hypomethylated |
|  | CD3D | 0.11644395 | 0.016107599 | NM_000732 | Hypomethylated |
|  | LHCGR | 0.152270891 | 0.016855042 | NM_000233 | Hypermethylated |
|  | ZNF501 | 0.173747967 | 0.033473437 | NM_145044 | Hypermethylated |
|  | FAM19A4 | 0.217803741 | 0.003091864 | NM_001005527 | Hypermethylated |
|  | GPR149 | 0.156230384 | 0.047399559 | NM_001038705 | Hypermethylated |
|  | EREG | 0.219778079 | 0.005244438 | NM_001432 | Hypermethylated |
|  | PCDHAC2 | 0.173317067 | 0.049385319 | NM_031861 | Hypermethylated |
|  | PCDHB16 | 0.201370413 | 0.01504728 | NM_020957 | Hypermethylated |
|  | PCDHB11 | 0.221397798 | 0.015676353 | NM_018931 | Hypermethylated |
|  | PCDHGB7 | 0.102454831 | 0.032863705 | NM_032101 | Hypermethylated |
|  | ELAVL2 | 0.301884632 | 4.77E-05 | NM_004432 | Hypermethylated |
|  | ZNF560 | 0.27700109 | 0.000177544 | NM_152476 | Hypermethylated |
|  | HKR1 | 0.173093667 | 0.016492647 | NM_181786 | Hypermethylated |
|  | NNAT | 0.123240265 | 0.047400739 | NM_005386 | Hypermethylated |
|  | DSCR6 | 0.132084428 | 0.042211139 | NM_018962 | Hypermethylated |
|  | ARF6 | 0.074397933 | 0.023431079 | NM_001663 | Hypomethylated |
| **t(8;21) AML CGIs, P < 0.05** | LHCGR | 0.435452379 | 0.021795084 | NM_000233 | Hypermethylated |
|  | FAM19A4 | 0.13631079 | 0.014309141 | NM_182522 | Hypermethylated |
|  | GPR149 | 0.397192766 | 0.019793536 | NM_001038705 | Hypermethylated |
|  | CRHBP | 0.368458302 | 0.019229988 | NM_001882 | Hypermethylated |
|  | PCDHAC2 | 0.413549617 | 0.029743223 | NM_018902 | Hypermethylated |
|  | PCDHB14 | 0.217743134 | 0.040831837 | NM_018934 | Hypermethylated |
|  | ELAVL2 | 0.356943532 | 0.038633918 | NM_004432 | Hypermethylated |
|  | ABLIM1 | 0.26685279 | 8.29E-05 | NM_001003408 | Hypermethylated |
|  | ZNF560 | 0.426668856 | 0.005016187 | NM_152476 | Hypermethylated |
|  | ZNF154 | 0.401566932 | 0.036694773 | NM_001085384 | Hypermethylated |

Genes that have absolute methylation difference labeled red are hypermethylated in MeDIP-seq, gene have absolute methylation difference labeled green are hypomethylated in MeDIP-seq.
