# Supplementary material for: Genome Wide Analysis of Acute Myeloid Leukemia Reveal Leukemia Specific Methylome and Subtype Specific Hypomethylation of Repeats
Source: PLoS One. 2012 Mar 29;7(3):e33213. doi: 10.1371/journal.pone.0033213 (PMC3315563; doi:10.1371/journal.pone.0033213)
Supplement: Table S13 — Direct bisulfite sequencing primers. (DOC) [file pone.0033213.s027.doc]

**Table S13. Direct bisulfite sequencing** primers

| **Genes** | **Primers** | **No of PCR cycles** |
| --- | --- | --- |
| ***SPHKAP*** | Forward: GTGTGAAATTTTTTTAAGTTGTGTT  Reverse: CAATAAATAACAACTCCCTACTCTC | 42 cycles |
| **CGIs** | Forward:TTGTTAGGTTTTGTTTATTATTTTTTTT  Reverse: CAAACCCAATCTCTCCATACAC | 42 cycles |
| **CGIs shores (a)** | Forward: AGTGTTTTTTTAGAAATTGGTTTG  Reverse:AAAAATTTCTTACTTCTTATAAAAAAC | 42 cycles |
| **CGIs shores (b)** | Forward: TTTAGTTGGGGTTGGTTGTTAATTA  Reverse: AAAACATATCAACACCTCCTTAAAC | 42 cycles |
| ***DPP6*** | Forward:GTTAATTTAGAGTGTAATTATGGAGA  Reverse: AAACTAAACTAAACTAAAAAACCTC | 40 cycles |
