# Supplementary material for: Genome Wide Analysis of Acute Myeloid Leukemia Reveal Leukemia Specific Methylome and Subtype Specific Hypomethylation of Repeats
Source: PLoS One. 2012 Mar 29;7(3):e33213. doi: 10.1371/journal.pone.0033213 (PMC3315563; doi:10.1371/journal.pone.0033213)
Supplement: Table S14 — Pyrosequencing primers. (DOC) [file pone.0033213.s028.doc]

**Table S14.** Pyrosequencing primers

| **Gene** | **Primers** |
| --- | --- |
| ***SPHKAP* chr2: 228753601-228753665** | F:GGTTTTATTTAGGGTAGAGTAGATT  *R:CCCCCTTCTTTCTATACCCAATACCATATC  S:ACCAACTTACCCCAA |
| ***DPP6* chr7: 153214816-153214843** | F:TGGGTAAAGAGGGTAGTTTTTTTTTAG  *R:TTCCCCACCTCTCCCTTCTCT  S:AGTTTTTTTTTAGATATTTT |
| ***ST6GAL2* chr2: 106869095-106869136** | F:GGAGGTTGTAGAGTTATTAAGAAAGG  *R:AAAACCCCCACCAAAATCCCA  S:AGGAGTGAGTGTTATATTTTATTTT |
| ***HHEX* chr10: 94442251-94442377** | F:GGAAGAGTTTGGTTAAGATGTTGTAGT  *R:ACCAAAAAACAAAATAACTCCT  S:GGTTAAGATGTTGTAGTTTAG |
| **AluJB chr7: 148474346-148474580** | F: AAGTTAGATTTTTGTTGTTTTTAGGAATTT  *R: TCCACCCCCCTTAACCTCCCAAAATA  S: GTTTGTAGTTTAAGATGT |

*Primer is biotinylated
